# Simultaneous Grafting of 3,4,5‐Trihydroxypiperidine Iminosugars Onto Multivalent Scaffolds via Double Reductive Amination Provides New GCase Inhibitors

**DOI:** 10.1002/chem.202502436

**Published:** 2025-12-08

**Authors:** Maria Giulia Davighi, Francesca Clemente, Alessio Morano, Francesca Mangiavacchi, Francesca Cardona, Andrea Goti, Paolo Paoli, Amelia Morrone, Ferran Nieto‐Fabregat, Roberta Marchetti, Camilla Matassini

**Affiliations:** ^1^ Department of Chemistry (DICUS) University of Florence Sesto F.no (FI) Italy; ^2^ Department of Experimental and Clinical Biomedical Sciences University of Florence Firenze Italy; ^3^ Laboratory of Molecular Genetics of Neurometabolic Diseases, Neuroscience and Human Genetics Department Meyer Children’s Hospital IRCCS Firenze Italy; ^4^ Department of Neurosciences, Psychology, Drug Research and Child Health University of Florence Firenze Italy; ^5^ Department of Chemical Sciences University of Naples Federico II Naples Italy

**Keywords:** double reductive amination, enzyme inhibition, GCase, molecular recognition, multivalent iminosugars

## Abstract

Low‐valency multivalent iminosugars have recently emerged as promising inhibitors of the therapeutically relevant enzyme β‐glucocerebrosidase (GCase). A new synthetic strategy has been developed to simultaneously build more than one trihydroxypiperidine iminosugar unit onto a polyamine scaffold via double reductive amination (DRA) of a d‐mannose derived dialdehyde. Five divalent derivatives, using both aliphatic and aromatic diamines and a trivalent compound based on an aromatic scaffold have been synthesized and evaluated as GCase inhibitors. Only oligomers with an aromatic core (**26**, **31**, and **37**) strongly inhibit GCase with IC_50_ values in the low micromolar range and activity enhancements, when compared to the monovalent counterpart, that confirm the occurrence of a positive multivalent effect. Kinetic analysis for divalent **31** and trivalent **37** revealed a mixed‐type inhibition. To rationalize the unexpected behavior of **37**, an integrated biophysical and computational approach based on STD‐NMR, docking, and MD simulations was employed, allowing to clarify the structural basis for its inhibitory profile and paving the way to the rational design of novel inhibitors able to bridge multiple interaction sites of the target enzyme.

## Graphical abstract

A small set of low‐valency multivalent 3,4,5‐trihydroxypiperidines was prepared through double reductive amination (DRA) of a sugar‐derived dialdehyde. Among them, trivalent derivative **37** emerged as a potent β‐glucocerebrosidase (GCase) inhibitor displaying an unexpected mixed‐type kinetic profile. The integration of kinetic analysis, NMR, docking, and MD simulations shed light on the molecular basis of the binding to the target enzyme.

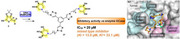

## 1 Introduction

**SCHEME 1:**
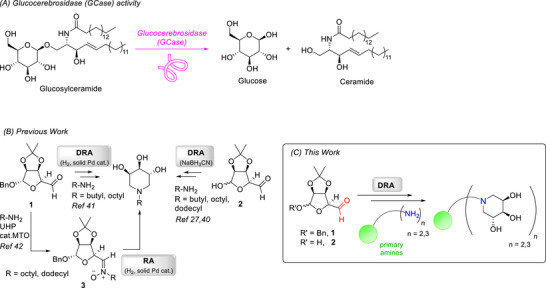
Hydrolysis of glucosylceramide catalyzed by lysosomal enzyme GCase (A). The double reductive amination (DRA) and the reductive amination (RA) approach for the synthesis of monovalent (B) and multivalent (C) trihydroxypiperidines.

The lysosomal enzyme β‐glucocerebrosidase (also known as GCase) cleaves the glycosidic bond of glucosylceramide to generate ceramide and release glucose (Scheme 1A) [1]. Defects in GCase activity result in accumulation of lipid substrates, which is associated to Gaucher's [2] and Parkinson's diseases [3, 4]. Therefore, compounds able to bind and rescue the activity of this enzyme have become attractive to address both pathologies [5, 6].

On the other hand, the inhibition of GCase activity has recently emerged to sensitize gynecologic cancer to chemotherapy [7, 8]. The role of GCase in cancer has not been completely understood, but significant increase of the enzyme level and the upregulation of its activity in several different cancer types (e.g. liver [9, 10], pancreatic [11], colon [12], gastric [13]) have been reported starting from 2016.

Most GCase inhibitors have been identified among iminosugars, glycomimetics with a nitrogen atom replacing the endocyclic oxygen of carbohydrates [14]. More recently, although first investigated in the field of carbohydrate‐lectin interactions [15, 16, 17, 18], multivalent ligands have emerged also as promising tools to modulate the inhibition of carbohydrate‐processing enzymes, such as glycosidases [19, 20, 21, 22, 23], by using iminosugars as inhitopes. In particular, the first report of a multivalent effect on an enzyme of therapeutic interest such as GCase dates back to 2012 and is due to the work of Compain and collaborators [24].

We recently found that 3,4,5‐trihydroxypiperidines featured with a lipophilic chain of at least eight carbon atoms [25, 26, 27] or an aromatic motif [28] have a great potential as inhibitors of GCase. In line with findings reported for other iminosugars [24, 29, 30, 31], our 3,4,5‐trihydroxypiperidine inhitope resulted particularly promising in its divalent [28, 32] or trivalent version [33].

To access piperidine iminosugars, the intramolecular reductive amination (RA) or the double reductive amination (DRA) reactions from carbohydrate precursors represent effective strategies. Indeed, carbohydrates possess the anomeric carbon, which can be seen as a “masked” aldehyde, and several primary and secondary hydroxyl groups which can be transformed into the appropriate nitrogen‐containing moiety to perform the intramolecular RA or selectively oxidized to access dicarbonyl compounds suitable for a DRA [34, 35].

This latter strategy, in particular, allowed the synthesis of a precursor of d‐isofagomine [36], of several DIX (1,5‐dideoxy‐1,5‐imino‐d‐xylitol) derivatives [37] and of 1‐deoxynojirimycin (DNJ) and 1‐deoxymannojirimycin (DMJ) [38, 39], which are well known piperidine iminosugars.

Following this strategy, our trihydroxypiperidine iminosugars were readily synthesized from d‐mannose‐derived “masked” dialdehydes **1** and **2** exploiting the DRA reaction in the presence of the appropriate lipophilic amine and reducing agent [27, 40, 41] or the intramolecular RA reaction *via* the nitrone intermediate **3**, in turn derived from **1** through condensation and subsequent *in situ* oxidation with urea hydrogen peroxide (UHP) and methyltrioxorhenium (MTO) catalyst (Scheme 1B) [42].

Multivalent iminosugars are commonly synthesized by multimerizing a preformed iminosugar with a terminal azido moiety onto a multivalent scaffold featured with several terminal alkynes, by exploiting the copper‐catalyzed azide‐alkyne cycloaddition (CuAAC) reaction [43, 44, 45]. However, the cytotoxicity of copper, especially if employed in the last step, can affect the evaluation of the biological activity of the final compounds, thus prompting for the development of alternative procedures [30, 31, 46, 47, 48, 49].

In this work, we propose for the first time the use of the DRA reaction as a strategy for the construction of multivalent trihydroxypiperidines using the aldehydes **1** or **2** with different divalent or trivalent amines in the presence of the appropriate reducing agent (Scheme 1C). Interestingly, this approach, besides avoiding possible drawbacks due to copper contamination, hooks to the scaffold the bioactive inhitope during its formation, thus simultaneously grafting 3,4,5‐trihydroxypiperidine iminosugars onto di‐ and trivalent scaffolds, which represents an absolute novelty in this field. These scaffolds constitute a significant variation with respect to those previously reported by some of us.

The inhibitory activity of the synthetic di‐ and tri‐valent compounds toward the target enzyme GCase was evaluated. Moreover, NMR spectroscopy and computational studies were employed to probe the interaction with GCase of selected compounds at the atomic level.

## 2 Results and Discussion

**SCHEME 2:**
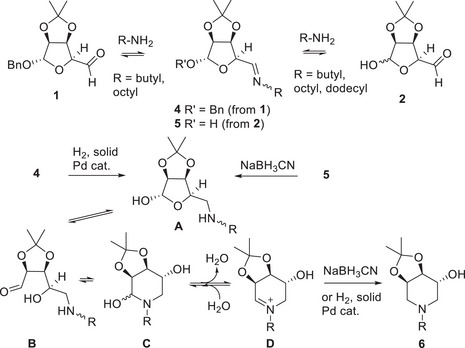
The steps involved in the double reductive amination (DRA) of aldehydes **1** and **2**.

The DRA on **1** or on **2** to afford **6** is a quite sluggish reaction which involves several steps in equilibrium: aldehyde/imine formation (**1**/**4** or **2**/**5**), sugar aldehyde/hemiacetal equilibrium (**B**/**A**), amine/hemiaminal equilibrium (**B**/**C**), hemiaminal/iminium ion formation (**C**/**D**) (Scheme 2). Thus, a careful tuning of the reaction conditions and monitoring the formation of the first imine intermediate is important to afford good yields. We therefore were aware that the achievement of this reaction in a multivalent fashion, multiplying the number of steps in dependence of the valency of the amine employed, would be a challenging task.

**SCHEME 3:**
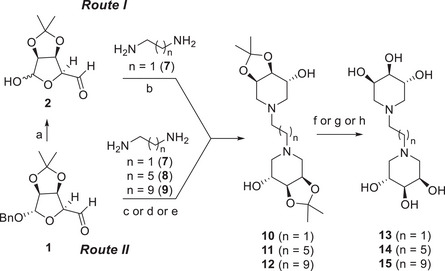
Reaction conditions: (a) H_2_, Pd(OH)_2_/C, rt, 18 h, quantitative; (b) 3Å MS, dry MeOH, NaBH_3_CN, AcOH, rt, 5 d, 16%; (c) reaction with ethylenediamine (n = 1, **7**), 3Å MS, dry MeOH, rt, 2 h, then H_2_, Pd(OH)_2_/C, rt, 6 d, 69%; (d) reaction with hexamethylenediamine (n = 5, **8**), MeOH, rt, 2 h, then H_2_, Pd(OH)_2_/C, rt, 18 h, 79%; (e) reaction with decamethylenediamine (n = 9, **9**), MeOH, rt, 1 h, then H_2_, Pd(OH)_2_/C, rt, 64 h, 79%; (f) from **10**, reaction with 12 M HCl, MeOH, rt, 20 h, then Ambersep 900‐OH, rt, 1 h, 86%; (g) from **11**, reaction with 12 M HCl, MeOH, rt, 18 h, then Ambersep 900‐OH, MeOH, rt, 16 h, 98%; (h) from **12**, reaction with 12 M HCl, MeOH, rt, 18 h, then Ambersep 900‐OH, MeOH, rt, 3 h, 95%.

As a first attempt, ethylenediamine (**7**) was used in the DRA reaction with the aldehyde **2**, obtained in turn from the catalytic hydrogenation of **1** [40] (Scheme 3). The reaction was performed with NaBH_3_CN and AcOH in dry MeOH in the presence of 3Å MS and afforded the divalent **10** in poor 16% yield (Scheme 3, *Route* *I*). We then tried to directly perform the DRA on the benzylated aldehyde **1** with ethylenediamine (**7**) using H_2_ (balloon) as the reducing agent and Pd(OH)_2_/C as the catalyst in dry MeOH as the solvent. In these conditions, the divalent **10** was obtained in a much higher 69% yield (Scheme 3, *Route II*). Acidic deprotection of **10** with HCl in MeOH, followed by the treatment with the strongly basic resin Ambersep 900‐OH, furnished the divalent trihydroxypiperidine **13** in 86% yield (Scheme 3).

Due to the low inhibitory activity observed for **13** (see biological evaluation), we skipped further optimization of this reaction and we decided to elongate the linker between the two trihydroxypiperidines employing hexamethylenediamine (**8**) as the nitrogen source (instead of the two‐carbon atom linker of ethylenediamine). According to the higher efficacy of *Route II*, the DRA was performed on the masked dialdehyde **1** with hexamethylenediamine (**8**), employing H_2_ as the reducing agent in the presence of Pd(OH)_2_/C as the catalyst. Nevertheless, several slight modifications in dialdehyde amount, reaction time and anhydrous conditions were tested to optimize the DRA reaction. The results of such optimization are shown in Table 1.

**TABLE 1:** The DRA on the masked aldehyde **1** with hexamethylenediamine (**8**).

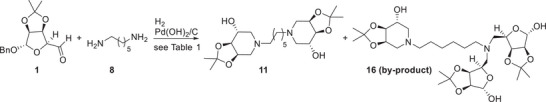					
Entry	1 ∶ 8 molar ratio	3Å Molecular sieves	Solvent	Time	Yield of 11 (%)^[^ ^a^ ^]^
1	2.4	yes	dry EtOH	11 d	^_^
2	2.0	no	dry MeOH	2 d	54
3	2.0	no	MeOH	18 h	79
4	1.8	no	dry MeOH	6 d	34

^[a]^ The yields of **11** were calculated based on the mmols of the limiting reagent.

In the first experiment a slight excess (1.2 equiv.) of aldehyde **1** per amine moiety of hexamethylenediamine (**8**) has been added (Entry 1, Table 1) in dry EtOH in the presence of 3Å MS and, after the formation of the imine intermediate (as attested by ^1^H‐NMR control), Pd(OH)_2_/C was added and the reaction was maintained under H_2_ atmosphere for 11 days. The crude revealed the presence of **11** in mixture with a by‐product showing the same *R_f_* of **11** and in agreement with the structure of the tertiary amine **16** (as attested by ESI‐MS of crude mixture and after its acetylation with excess acetic anhydride in pyridine, see the Supporting Information), which was formed in the reaction of a third molecule of **1** onto a secondary amine intermediate and resulting in the failure of the second piperidine ring‐closure reaction (see the Supporting Information for the whole mechanism proposed for the formation of **16**).

In the second experiment, (Entry 2, Table 1) stochiometric amount of the aldehyde **1** (1 equiv. of **1** for each amine moiety) reacted with hexamethylenediamine (**8**) in MeOH, without 3Å MS, and after catalytic hydrogenation, the desired **11** was obtained in a quite satisfying 54% yield (Scheme 3, Table 1). Surprisingly, the lack of 3Å MS appeared beneficial to the reaction in this case. This hypothesis was confirmed by performing the reaction using nondry MeOH, which led to an increase in the yield of **11** from 54% to 79% in shorter reaction time (Entry 3, Table 1). When we tried to further reduce the equivalents of aldehyde **1** in the DRA reaction (0.9 equiv. of **1** for each amine moiety, Entry 4, Table 1), compound **11** was obtained in a worse 34% yield although prolonging the reaction time. Deprotection of the acetonide groups of **11** under acidic conditions (HCl in MeOH) gave, after treatment with Ambersep 900‐OH resin, the dimer **14** in 98% yield (Scheme 3). Finally, the DRA reaction with decamethylenediamine (**9**) as the nitrogen source led to the 10‐carbon atom spaced divalent trihydroxipiperidine **15**, after deprotection of the precursor **12**, in 75% yield over two steps (Scheme 3).

**SCHEME 4:**
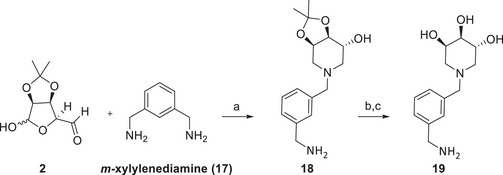
Reaction conditions: (a) dry MeOH, rt, 18 h, then NaBH_3_CN, AcOH, rt, 4 d, 24%; (b) 12 M HCl, MeOH, rt, 18 h; (c) Ambersep 900‐OH, MeOH, rt, 2 h, 57%.

Since in our experience the multimerization around an aromatic ring scaffold afforded good inhibitors of GCase [33], we next investigated the feasibility of the DRA reaction between *m*‐xylylenediamine (**17**) and the aldehyde **2** in order to set up the best conditions for the synthesis of dimers with a central rigid aromatic scaffold. However, the reaction of **2** with *m*‐xylylenediamine (**17**) (**2**  : **17** molar ratio = 2.1) in the presence of NaBH_3_CN and AcOH yielded only the monovalent **18** in low 24% yield (Scheme 4), and no formation of the desired divalent derivative was observed, probably due to steric hindrance of the two trihydroxypiperidine that should form around *m*‐xylylenediamine (**17**) in this reaction. The lower nucleophilicity of the benzylic nitrogen atoms of **17** with respect to alkylamines **7‐9** may slow down the imine formation until preventing the second piperidine formation. The catalytic hydrogenation conditions were not viable with this scaffold since the cleavage of the benzylic N‐C bond was envisaged. Acidic deprotection of **18** with HCl in MeOH followed by the treatment with Ambersep 900‐OH resin gave **19** in 57% yield (Scheme 4).

**SCHEME 5:**
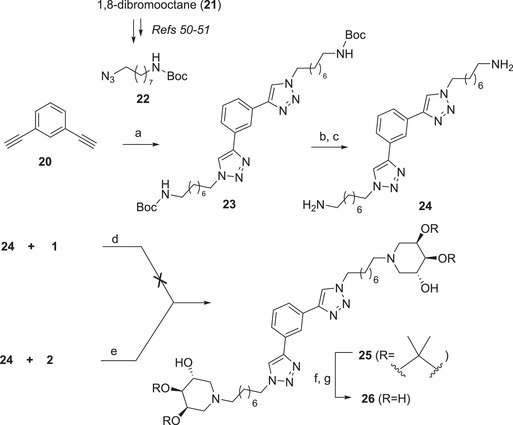
Reaction conditions: (a) **22**, CuSO_4_, sodium ascorbate, THF/H_2_O 2:1, MW 80 °C, 45 min, 75%; (b) TFA, dry DCM, rt, 4 h; (c) Ambersep 900‐OH, MeOH, rt, 2 h, quantitative; (d) dry EtOH, rt, 18 h, then H_2_, Pd(OH) _2_/C, rt, 14 d; (e) MeOH, CHCl_3_, reflux, 2 h, then NaBH_3_CN, AcOH, reflux, 43 h, 37%; (f) 12 M HCl, MeOH, rt, 18 h; (g) Ambersep 900‐OH, MeOH, rt, 2 h, 77%.

With the aim of distancing the 3,4,5‐trihydroxypiperidine inhitope from the aromatic scaffold to reduce the steric hindrance and increase affinity with the GCase enzyme with the introduction of an alkyl linker, we took in consideration di‐ and triamines derived from the functionalization of aromatic di‐ and trialkynyl compounds. Since good results were previously obtained with aliphatic chains of eight carbon atoms [25, 26], we first prepared the amino azide **22** (Scheme 5), with the Boc‐protected amino moiety, starting from 1,8‐dibromooctane (**21**) and following previously reported procedures [50, 51]. The CuAAC reaction between **22** and the commercially available *meta*‐dialkynyl benzene (**20**) with a catalytic amount of CuSO_4_ and sodium ascorbate in a 2:1 THF/H_2_O mixture under MW irradiation afforded the expected divalent derivative **23** in 75% yield (Scheme 5). The acidic treatment of **23** with trifluoroacetic acid (TFA) in dry CH_2_Cl_2_ for 4 h followed by the treatment with Ambersep 900‐OH resin afforded quantitatively the free diamine **24**, pure enough to be employed in the next step. The first DRA reaction to synthesize **25** was performed with the benzylated aldehyde **1** and the diamine **24** using H_2_ as reducing agent and Pd(OH)_2_/C as the catalyst in dry EtOH (Scheme 5). Unfortunately, in these conditions, the reaction did not reach completion even after 2 weeks, the corresponding crude resulted very complex to purify and we failed in isolating pure **25**.

We therefore turned back to NaBH_3_CN as the reducing agent starting from aldehyde **2** and employing slightly different reaction conditions with respect to the above mentioned ones (in particular, addition of CHCl_3_, and reflux temperature), which in our hands had previously proven satisfactory with similar piperidine iminosugars bearing an aromatic substituent [52, 53]. Refluxing a mixture of **24** and **2** with NaBH_3_CN and AcOH in MeOH and CHCl_3_ at afforded pure **25** in 37% yield, after purification by flash column chromatography (FCC). Despite the apparently low overall yield, it must be considered that the occurring reductive amination is a cascade process comprising several steps which, when taken individually, proceed with a high yield. Deprotection of **25** with HCl in MeOH followed by the treatment with Ambersep 900‐OH furnished the corresponding deprotected divalent **26** in 77% yield (Scheme 5).

**SCHEME 6:**
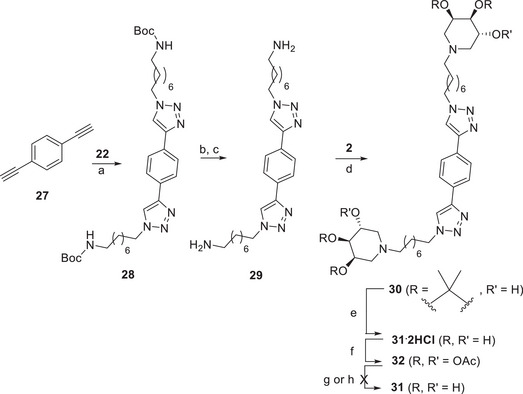
Reaction conditions: (a) **22**, CuSO_4_, sodium ascorbate, THF/H_2_O 2:1, MW 80 °C, 1 h 15 min, 61%; (b) TFA, dry DCM, rt, 4 h; (c) 32% NH_4_OH, MeOH, rt, 20 min, quantitative; (d) NaBH_3_CN, AcOH, MeOH, CHCl_3_, reflux, 2 d, 43%; (e) HCl 12 M, MeOH, rt, 18 h; (f) Ac_2_O, dry piridine, rt, 18 h, 47% over two steps; (g) Na_2_CO_3_, MeOH, rt, 18 h; (h) Ammonia solution (4 M ammonia in methanol; MercK), rt, 42 h.

A similar synthetic strategy was carried out to obtain the analogous *para* divalent derivative **31**. The CuAAC reaction of *para*‐dialkynyl benzene **27** with the amino azide **22** with catalytic CuSO_4_ and sodium ascorbate under MW irradiation at 80 °C in THF/H_2_O yielded, after purification by FCC, the protected amine **28** in 61% yield (Scheme 6). In this case, the obtainment of the free diamine **29** was not trivial. In fact, after the acid deprotection with TFA (attested by the ^1^HNMR spectrum of the crude), the treatment with strongly basic resin Ambersep 900‐OH resin allowed to isolate the free diamine **29** only in traces.

We therefore succeeded in obtaining quantitatively the free diamine **29** by performing the Boc group deprotection with TFA in dry CH_2_Cl_2_ followed by the treatment with an excess of ammonia solution (32% NH_4_OH) in MeOH (Scheme 6). The DRA procedure performed by refluxing **29** and the aldehyde **2** with NaBH_3_CN and AcOH in a mixture of MeOH and CHCl_3_ gave the divalent **30** in 43% yield (Scheme 6). Treatment of **30** with HCl in MeOH at room temperature for 18 h gave the hydrochloride salt **31·2HCl**. Attempts to obtain pure **31** as free diamine failed even after protection to **32** and subsequent deprotection (see Supporting Information). We therefore decided to employ **31** as hydrochloride salt in the biological screening, as already reported for similar multivalent compounds for which purification problems were encountered for the corresponding free amines [54].

**SCHEME 7:**
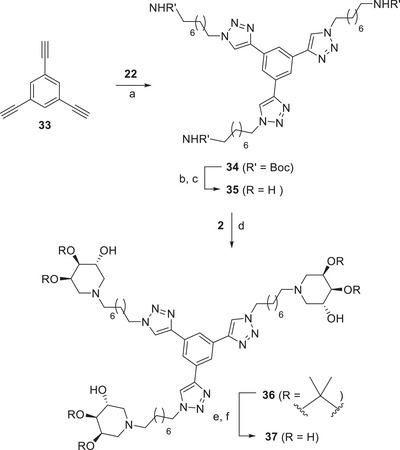
Reaction conditions: (a) CuSO_4_, sodium ascorbate, THF/H_2_O 2:1, MW 80 °C, 1 h, 82%; (b) TFA, dry DCM, rt, 4 h; (c) Ambersep 900‐OH, MeOH, rt, 5 h, quantitative; (d) from **35**, NaBH_3_CN, AcOH, MeOH, CHCl_3_, reflux, 40 h, 35%; (e) HCl 12 M, MeOH, rt, 18 h; (f) Ambersep 900‐OH, MeOH, rt, 2 h, 94%.

To further increase the valency of the systems prepared via DRA reaction, we decided to introduce three trihydroxypiperidines on the trivalent scaffold, the commercially available 1,3,5‐trialkynyl benzene (**33**). The CuAAC reaction of **33** with **22** in presence of a catalytic amount of CuSO_4_ and sodium ascorbate in a 2:1 THF/H_2_O mixture under MW irradiation at 80°C for 1 h afforded the Boc‐protected triamine **34** in 82% yield (Scheme 7). The acidic treatment of **34** with TFA in dry DCM for 4 h furnished, after treatment with Ambersep 900‐OH resin, the free triamine **35** (quantitative yield) pure enough to be used in the following step (Scheme 7). The debenzylated aldehyde **2** was refluxed with the triamine **35** in the presence of NaBH_3_CN and AcOH in a mixture of MeOH and CHCl_3_ to afford, after purification by FCC, the pure **36** in 35% yield (Scheme 7). Acidic deprotection of **36** with HCl in MeOH followed by the treatment with Ambersep 900‐OH furnished the corresponding deprotected trivalent **37** in 94% yield (Scheme 7).

**SCHEME 8:**
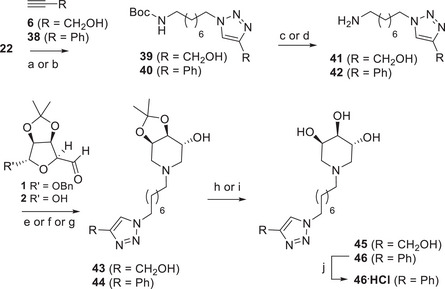
Reaction conditions: (a) reaction with propargyl alcohol (**6**), CuSO_4_, sodium ascorbate, THF:H_2_O 2:1 rt, 16 h, 84% of **39**; (b) reaction with phenylacetylene (**38**), CuSO_4_, sodium ascorbate, THF:H_2_O 2:1 rt, 16 h, 89% of **40**; (c) from **39**, reaction with TFA, dry DCM, rt, 6 h, then Ambersep 900‐OH, MeOH, rt, 24 h, 60% of **41**; (d) from **40**, reaction with TFA, dry DCM, rt, 4 h, then Ambersep 900‐OH, MeOH, rt, 16 h, quantitative yield of **42**; (e) from **41**, reaction with **2**, MeOH, CHCl_3_, reflux, 2 h, then NaBH_3_CN, AcOH, reflux, 3 d, 47% of **43**; (f) from **41**, reaction with **1**, dry MeOH, rt, 18 h, then H_2_, Pd(OH)_2_/C, rt, 8 d, 41% of **43**; (g) from **42**, reaction with **2**, MeOH, CHCl_3_, reflux, 2 h, then NaBH_3_CN, AcOH, reflux, 2 d, 43% of **44**; (h) from **43**, reaction with HCl 12 M, MeOH, rt, 18 h, then Ambersep 900‐OH, rt, 2 h, 87% of **45** (i) from **44**, reaction with 12 M HCl, MeOH, rt, 18 h, then Ambersep 900‐OH, rt, 2 h, quantitative yield of **46**; (j) 12 M HCl, MeOH, rt, 2 h, quantitative.

For the sake of comparing the biological activity of the new multivalent compounds with a monovalent reference compound, the two slightly different monovalent derivatives **45** and **46** shown in Scheme 8 were prepared, besides compound **19** obtained by unexpected product. They were designed as bearing the triazole ring ending with: i) a saturated hydroxymethyl group (**45**), according to analogue reference compounds previously reported by us [33] and others [55, 56, 57] or ii) a benzene ring (**46**), to better mimic the aromatic core present in the di‐ and trivalent derivatives described in Schemes 5, 6, 7.

The CuAAC reaction of the azide **22** with propargyl alcohol (**6**) or with phenylacetylene (**38**) in the presence of catalytic CuSO_4_ and sodium ascorbate in THF and H_2_O afforded triazole derivatives **39** and **40** in 84% and 89% yields, respectively, after FCC (Scheme 8). The corresponding free amines **41** (60% yield) and **42** (quantitative yield) were achieved after the acidic deprotection with TFA in dry CH_2_Cl_2_ for 6 h and 4 h, respectively, followed by the treatment with Ambersep 900‐OH resin. The dihaldehyde **2** was first refluxed with the amine **41** in the presence of NaBH_3_CN as the reducing agent and AcOH in a mixture of MeOH and CHCl_3_ to afford the pure piperidine derived scaffold **43** in 47% yield after FCC. Compound **43** was also obtained with similar yield (41%) performing the DRA from **41** and the benzylated aldehyde **1** in dry MeOH using H_2_ as reducing agent and Pd(OH)_2_/C as catalyst (see the Supporting Information). Similarly, the DRA reaction of **2** with the amine **42** with NaBH_3_CN and AcOH in a mixture of MeOH and CHCl_3_ at reflux furnished the compound **44** in 43% yield. However, in this case **44** could not be obtained from **1** and **42** in catalytic hydrogenation conditions, since the reaction did not go to completion even after 2 weeks. Acidic deprotection of **43** and **44** with HCl in MeOH followed by the treatment with Ambersep 900‐OH furnished the trihydroxypiperidines **45** and **46** in 87% and quantitative yields, respectively. Considering the poor solubility of compound **46** in most organic deuterated solvents (e.g. CDCl_3_, CD_3_OD, THF‐d8, D_2_O, acetone‐d6), we decided to transform it into the corresponding hydrochloride acid **46** · **HCl** by treatment with 12 M HCl in MeOH, for the purpose of performing the NMR characterization (Scheme 8).

Determination of fundamental inhibitory properties and binding mechanisms of the novel compounds represents the foundation for future studies, either as promising pharmacological chaperone or chemotherapy sensitizer candidates. Thus, the divalent trihydroxypiperidines **13**, **14**, **15**, **26**, **31** and the trivalent derivative **37** were evaluated as inhibitors of the human GCase enzyme through a screening in extracts from a pool of human leucocytes isolated from healthy donors (1 mM inhibitor concentration, 37°C and 5.8 pH, see the Experimental Section) and are summarized in Table 2. The inhibitory activity of the monovalent reference compounds **45** and **46**, as well as the monovalent **19**, were also reported to determine the relative potency (rp) and relative potency per bioactive unit (rp/n) values.

**TABLE 2:** IC_50_ values and type of inhibition *vs* GCase derived from human leukocyte homogenate of healthy donors of new compounds.

Entry	Compound^[^ ^a^ ^]^	Valency (n)	GCase inhibition (%)^[^ ^b^ ^]^	IC_50_ (mM)^[^ ^c^ ^]^	rp^[^ ^d^ ^]^	rp/n^[^ ^d^ ^]^
1	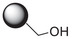 **45**	1	20	1.6 ± 0.16	_	_
2	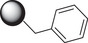 **46**	1	95	0.084 ± 0.020 non competitive inhibitor (K*i* = 51.0 ± 2.9 µM)	_	_
3	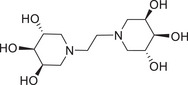 **13**	2	17	nd	nd	nd
4	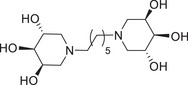 **14**	2	22	nd	nd	nd
5	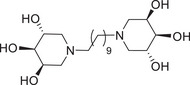 **15**	2	24	nd	nd	nd
6	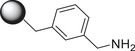 **19**	1	1	nd	_	_
7	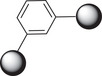 **26**	2	93	0.016 ± 0.010 pure competitive inhibitor (K*i* = 3.30 ± 0.24 µM)	100^[^ ^e^ ^]^ (5)^[^ ^f^ ^]^	50^[^ ^e^ ^]^ (2.5)^[^ ^f^ ^]^
8	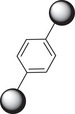 **31**	2	98%^[^ ^g^ ^]^	0.0080 ± 0.0004 mixed type inhibitor (K*i* = 6.0 ± 0.1 µM, K*i*’= 14.5 ± 0.5 µM)	200^[^ ^e^ ^]^ (10.5)^[^ ^f^ ^]^	100^[^ ^e^ ^]^ (5.3)^[^ ^f^ ^]^
9	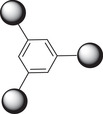 **37**	3	89	0.020 ± 0.010 mixed type inhibitor (K*i* = 9.7 ± 0.5 µM, K*i*’= 15.2 ± 0.4 µM)	80^[^ ^e^ ^]^ (4.2)^[^ ^f^ ^]^	27^[^ ^e^ ^]^ (1.4)^[^ ^f^ ^]^
10	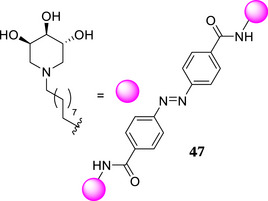	2	100	0.0070 ± 0.0001 mixed type inhibitor (K*i* = 4.1 ± 0.4 µM and K*i’* = 21.2 ± 4.9 µM) [32]	3.4	1.7
11	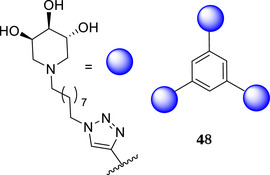	3	100	0.0070 ± 0.0010 pure competitive inhibitor [33] (K*i* = 3.1 ± 0.2 µM)	71	24

^[a]^
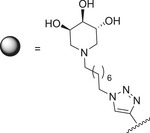
.

^[b]^ Percentage inhibition of GCase in human leukocytes extracts incubated with the inhibitor (1 mM).

^[c]^ IC_50_ values were determined by measuring GCase activity at different concentrations of each inhibitor. nd: non determined.

^[d]^ Calculated based on IC_50_ values.

^[e]^ Evaluated with respect to the monovalent reference compound **45**.

^[f]^ Evaluated with respect to the monovalent reference compound **46**.

^[g]^ Tested at 0.1 mM concentration.

Divalent compounds **13** and **14** resulted negligible inhibitors of GCase (17% and 22% inhibition at 1 mM respectively, Entries 3 and 4, Table 2), showing that the presence of an alkyl chain of at least eight carbon atoms is essential to impart GCase inhibition, as previously observed [25, 26]. However, even the longer 10‐carbon atom spacer connecting the two 3,4,5‐trihydroxypiperidine warheads of **15** did not prove beneficial for the affinity with the target enzyme, since only 24% inhibition was observed in this case (Entry 5, Table 2). Conversely, the divalent compounds **26** and **31**, and the trivalent compound **37**, in which the central benzene scaffold is connected to the trihydroxypiperidine units through an octyl chain, were very potent GCase inhibitors (89%–98% inhibitory activity, IC_50_ values between 8.0 and 20 µM, Entries 7‐9, Table 2). However, the total absence of inhibition observed for monovalent **19** (the mono‐reacted product of the DRA of dialdehyde **2** with *m*‐xylylenediamine) suggested that the presence of the phenyl ring alone, close to the trihydroxypiperidine unit, is not sufficient to guarantee affinity toward the GCase enzyme.

The relative potency (rp) values for multivalent compounds **26**, **31**, and **37** with respect to monovalent derivatives were obtained by dividing the IC_50_ of the monovalent reference compound by the IC_50_ of the divalent or trivalent derivatives (Table 2). As previously observed in the context of divalent glycosidase inhibitors, [58] rp values strongly depend on the choice of the monovalent reference inhibitor. Therefore, rp values for compounds **26**, **31** and **37** are in the 80‐200 range if calculated with respect to compound **45** (featuring a triazole with a terminal hydroxymethyl moiety) but dramatically drop to 10.5‐4.2 if calculated on the more active monovalent reference compound **46** (with a terminal phenyl ring). This also suggests that enhanced binding is due to the aromatic moiety of the scaffold, which exerts a synergistic effect, as also recently observed in the context of GALNS (*N*‐acetylgalactosamine 6‐sulfatase) inhibition by multivalent ligands [59]. In any case, considering the valency (which means dividing rp by n) the divalent derivatives **26** and **31**, and the trivalent derivative **37** were more active than both the monovalent counterparts **45** and **46** (rp/n values higher than 1), showing the role played by multivalency [21].

Prompted by these promising data, we determined the inhibition constant (K*i*) and the mode of inhibition of multivalent compounds **26**, **31**, **37**, and monovalent **46** by Lineweaver–Burk plots (see the Supporting Information).

Interestingly, the divalent compound **26** behaves as a pure competitive inhibitor of GCase (K*i* = 3.30 ± 0.24 µM), in contrast to the mixed type inhibition previously observed for the divalent architectures **47** conjugated with an azobenzene moiety (Entry 7 vs Entry 10, Table 2) [32]. A mixed type behavior has been reported for glycosidase inhibition by other multivalent glycomimetics [60, 61]. A noncompetitive inhibition was observed for the monovalent **46** (K*i* = 51.0 ± 2.9 µM, Entry 2, Table 2). The latter result is in line with our own and literature data, in which some piperidine iminosugars bearing aromatic groups are reported to behave as noncompetitive GCase inhibitors [28, 32, 62, 63]. Conversely, the divalent compound **31** and the trivalent compound **37** behave as mixed type inhibitors binding both the active site of the free enzyme in competition with the substrate (K*i* = 6.0 ± 0.1 µM for **31** and K*i* = 9.7 ± 0.5 µM for **37**) and an allosteric site on the enzyme‐substrate complex (K*i*’ = 14.5 ± 0.3 µM for **31** and K*i*’ = 15.2 ± 0.4 µM for **37**) (Entry 8–9, Table 2). This peculiar ability of trivalent compound **37** diverges from the pure competitive inhibition previously observed with the similar trivalent compound **48** (Entry 11, Table 2). This different behavior appears particularly unexpected because the trivalent compounds **37** and **48** share *exactly the same structure* except for an additional methylene group in the alkyl chain linker in case of **48**, resulting in a slightly longer distance between triazole ring and the endocyclic nitrogen of the 3,4,5‐trihhydroxipiperidine unit.

**FIGURE 1:**
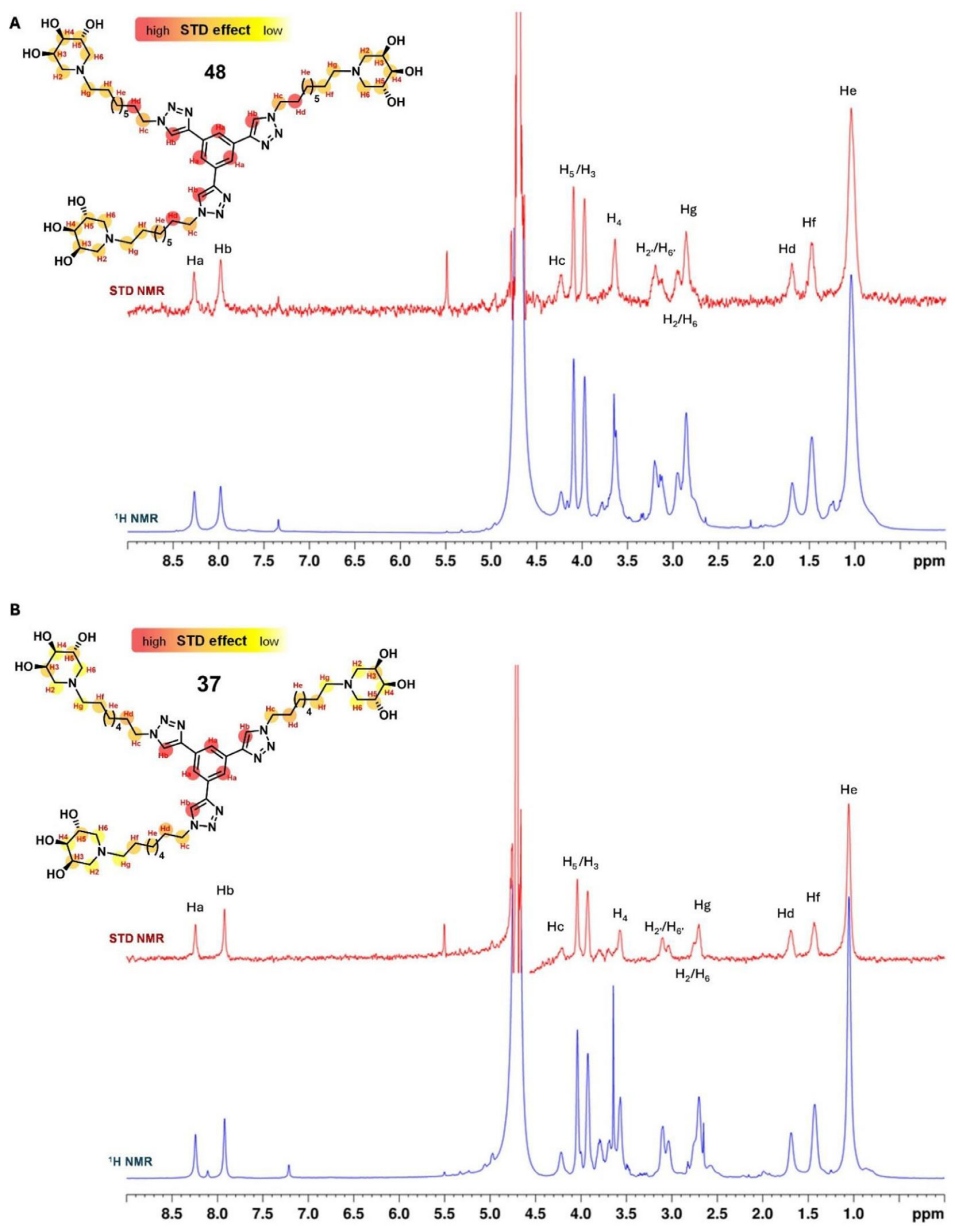
Saturation transfer difference (STD) NMR analysis of **37** and **48** binding to GCase. A) Structural representation of **48** with mapped STD intensities—from highest (in red) to lowest (in yellow)—upon interaction with GCase. The reference 1D 1H NMR spectrum is depicted in blue and STD NMR spectrum in red. B) Epitope mapping of **37** binding to GCase, using the same color scale and spectral layout as **48**.

With the aim to rationalize the distinct behavior of the two structurally related trivalent ligands **37** and **48** toward GCase, an integrated biophysical and computational approach was employed. Saturation transfer difference (STD) NMR experiments were carried out to probe the interaction of both compounds, **37** and **48**, with GCase (Figure 1). STD NMR is a well‐established technique for mapping the contact surface between a ligand and its target protein, typically allowing the identification of ligand moieties involved in the interaction with the receptor protein [64, 65]. However, in the case of symmetric multivalent ligands, such as **37** and **48**, the severe isochrony of several proton signals precluded the unambiguous assignment of individual protons involved in the interaction. Despite this limitation, a qualitative analysis of the STD NMR spectra, acquired under identical experimental conditions, revealed a different recognition of **37** compared to **48** by the target enzyme. While, in both cases, the aromatic moieties showed the highest STD NMR enhancements, indicating their predominant role in both molecular recognition processes, the overall fingerprint of the two STD NMR spectra was different. In particular, the STD NMR signals belonging to the resonances of the iminosugar units in the STD NMR spectrum acquired on GCase/**48** mixture exhibited higher enhancements (Figure 1) thus suggesting a different binding mode compared to **37**.

**FIGURE 2:**
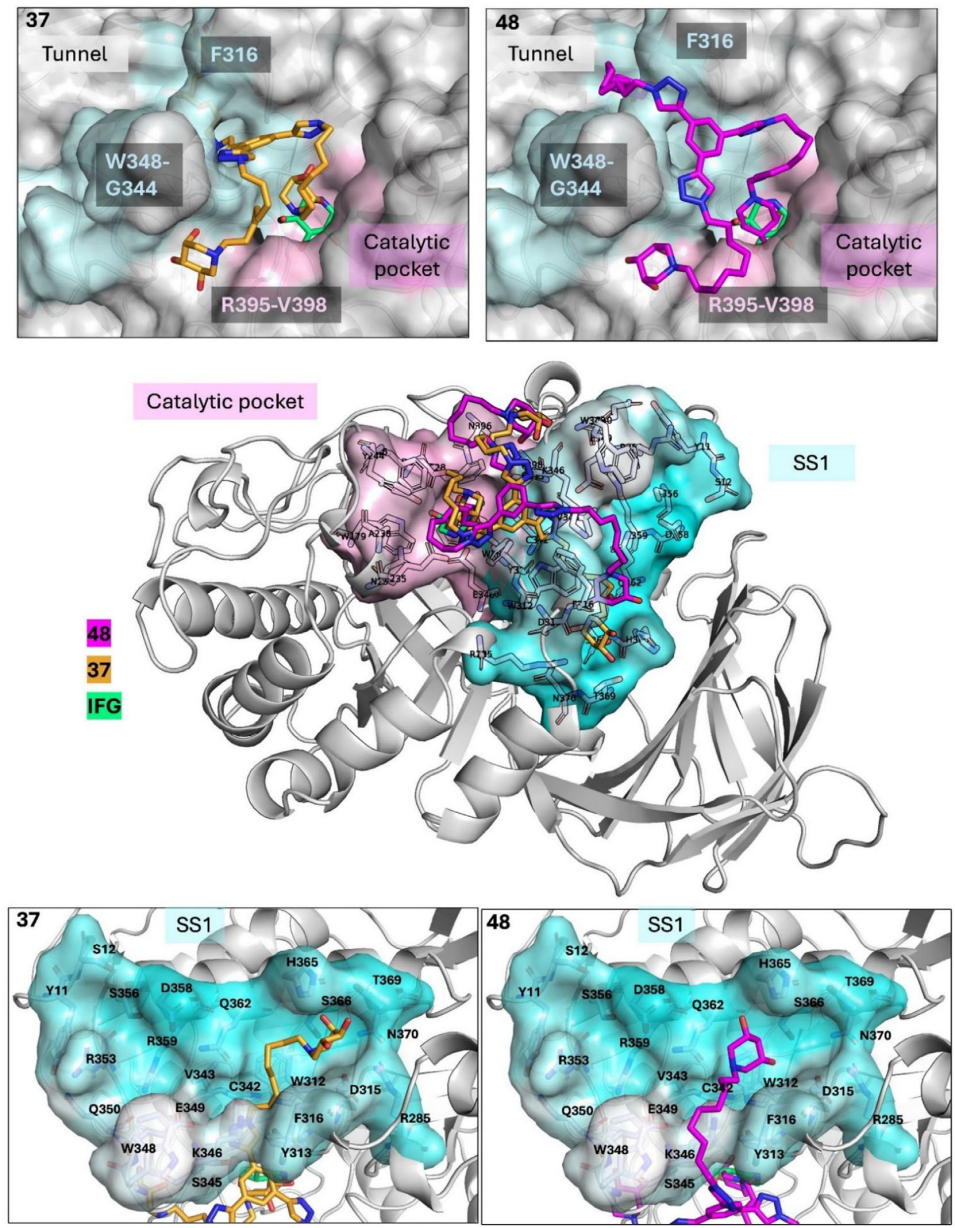
3D model of **37** (gold) and **48** (magenta) in complex with GCase. The central panel shows an overview of both ligands upon binding. Close‐up views of each compound ‐ **37** (on the left) and **48** (on the right) ‐ accomodated within the catalytic pocket (pink) and compared to the IFG (green) binding mode are reported in the upper panel. Blow‐up representations of **37** (on the left) and **48** (on the right) within the SS1 region (cyan) of the enzyme are reported in the lower panel.

To gain additional insight into how **37** and **48** might interact with GCase, computational studies were performed. First of all, both compounds were parameterized through a combination of Gaussian‐based geometry optimization and GAFF2 force field assignment, ensuring accurate conformational sampling prior to docking calculations. With the parametrized ligands in hand, the cavity mapping was carried out to identify potential interaction sites on the protein surface. The cavity search revealed two main binding regions (Figure 2 and Figure S16). The first corresponded to the known catalytic pocket, where the substrate and well‐known GCase competitive inhibitor—isofagomine (IFG)—bind [66]. The detection of this site suggests that the iminosugar moieties of both **37** and **48** are sterically compatible with the catalytic cleft, supporting the hypothesis of a competitive binding mode. In addition, a secondary surface cavity (SS1) was identified adjacent to the catalytic pocket, extending along a solvent‐accessible tunnel defined by loops L314–L317 and K346–E349. This elongated groove is lined with a combination of hydrophobic and polar residues, including several aromatic and charged side chains—such as F316, W348, and H365 ‐ that are well‐positioned to mediate stabilizing interactions. The geometry of this cavity, clearly visible in the surface representation (Figure S16), suggests it may act as an allosteric site capable of accommodating one of the distal branches of the trivalent ligands. Interestingly, the location and shape of SS1 closely match previously reported allosteric pockets implicated in noncompetitive inhibition [32].

To further explore how the two compounds are accommodated in these regions, docking and Molecular Dynamics MD calculations were subsequently performed [67]. The docking output revealed that both **37** and **48** displayed high binding affinities, with top‐ranked poses scoring −8.7 kcal/mol and −8.2 kcal/mol, respectively. Moreover, in both cases, one of the three branches was accommodated within the catalytic pocket (Figure 2). Nevertheless, the iminosugar unit of **48** was more deeply inserted into the catalytic cavity with respect to **37**, closely mimicking the IFG binding pose (Figure 2). This difference in the accommodation of the two compounds may account for the stronger STD NMR signals observed in the iminosugar region of **48** compared to **37**. However, the most striking difference was observed in the trajectory of the second arm. Probably due to its shorter alkyl chain, **37** was able to pass through the tunnel formed by loops L314–L317 and K346–E34, extending further into the groove and connecting the catalytic pocket to the internal cavity SS1. Despite its close structural similarity, **48** was sterically hindered from entering this tunnel due to the presence of the additional methylene group in its alkyl chains. Thus, it followed an alternative path: the arm passed over the tunnel and differently oriented the iminosugar within the SS1 region compared to **37**. Finally, the third arm of both ligands was consistently directed toward a shallow, solvent‐exposed groove adjacent to the catalytic cleft, which was occupied in a similar manner by both **37** and **48**.

Taken together, these data suggested two distinct binding modes: **48** engages GCase primarily through deep insertion into the catalytic pocket, while **37** capitalizes on its compact geometry to bridge all three sites, resulting in a more spatially distributed multivalent mode of recognition.

## 3 Conclusion

In summary, we demonstrated that the DRA reaction on a sugar‐derived dialdehyde with a polyamine can be exploited for the synthesis of multivalent iminosugars by forming the piperidine iminosugar skeleton simultaneously to its anchoring to the central scaffold. Both aliphatic and aromatic polyamines have been tested as scaffolds, highlighting that, for the former, a catalytic hydrogenation procedure was more efficient, while for the latter the use of NaBH_3_CN as the reducing agent performed best. All final di‐ and trivalent compounds were easily purified with the exception of the aromatic *p*‐substituted divalent derivative **31**, which was maintained as hydrochloride in the subsequent biological screening.

The inhibitory activity assay toward human GCase highlighted that a central aromatic scaffold is essential to guarantee the affinity for the target enzyme, with the lower IC_50_ values observed for divalent **26** (IC_50_ = 16 µM) and **31** (IC_50_ = 8 µM) and trivalent **37** (IC_50_ = 20 µM).

However, an in‐depth kinetic analysis revealed that *meta*‐substituted divalent **26** behaves as a pure competitive inhibitor, while the *para*‐substituted divalent **31** is a mixed type inhibitor, suggesting that a different spatial distribution of the iminosugar units around the aromatic core may vary the binding sites involved in the protein interaction. Even more intriguing, also the trivalent derivative **37** showed a different kinetic profile with respect to its previously reported analogue **48**, which simply bears a slightly longer linker between the triazole and the endocyclic nitrogen of the iminosugar unit (nonyl in **48** *vs* octyl in **37**) per arm. While trivalent **48** acts as a pure competitive inhibitor, the newly reported trivalent **37** shows a mixed‐type inhibition, binding both the active site of the free enzyme and an allosteric site on the enzyme‐substrate complex.

The combined application of ligand‐based NMR spectroscopy and computational studies allowed us to elucidate the molecular basis for the divergent inhibitory behavior of the trivalent compounds **37** and **48**.

The fingerprint of the STD NMR spectra and the differences in the STD NMR intensities clearly indicated a different binding mode between **37** and **48**. Indeed, although both compounds interact with the enzyme surface primarily through their aromatic moieties, **48** exhibited more pronounced saturation transfer to the iminosugar protons than **37**, suggesting a different accommodation within the protein binding sites. These results were further confirmed by computational studies, including cavity mapping, docking, and MD simulations, which allowed us to describe the spatial arrangement of **37** and **48** upon binding. The resulting data indicated that one of the three branches of both **37** and **48** occupied the catalytic pocket, placing the iminosugar moiety in direct contact with key active site residues. Interestingly, the iminosugar in **48** was found to be more deeply inserted into the cleft compared to **37** that adopted a different orientation with the terminal iminosugar less deeply penetrating the enzyme cavity. The second arm of each ligand showed a more divergent behavior: the shorter alkyl chain of **37** was able to pass through a tunnel close to the catalytic cleft, thereby accessing farther regions on the enzyme surface. By contrast, the longer alkyl chain of **48** was sterically hindered from entering this tunnel and instead passed over it, thereby differently positioning the iminosugar moiety within the SS1 region. Finally, the third arm was directed in both ligands toward a solvent‐exposed groove adjacent to the catalytic site.

In conclusion, the integration of kinetic analysis, NMR, docking, and MD simulations has uncovered a significant distinction in the binding modes of **37** and **48**. The catalytic pocket serves as a stable and common recognition site for both ligands, but only **37** is able to exploit the spatial extension of the enzyme surface by bridging multiple interaction sites.

These findings not only clarify the structural basis for the different inhibition profile of **37** and **48** but also highlight the potential of targeting interdomain corridors and secondary cavities in the rational design of next‐generation of GCase inhibitors.

## 4 Experimental Section

### General Procedures

Reagents were purchased from commercial suppliers and used without purification. All reactions were carried out under magnetic stirring and monitored by TLC on 0.25 mm silica gel plates with fluorescent indicator. Flash Column Chromatography (FCC) was carried out on Silica Gel (230–400 mesh). Yields refer to spectroscopically and analytically pure compounds unless otherwise stated. ^1^H‐NMR and ^13^C‐NMR spectra of the synthesized molecules were recorded on a Varian Gemini 200 MHz, a Varian Mercury 400 MHz or on a Varian INOVA 400 MHz instrument at 25°C. ^1^H‐NMR and ^13^C‐NMR spectra were referenced against the residual solvent signal [68]. Integrals are in accordance with assignments, coupling constants are given in Hz. For detailed peak assignments 2D spectra were measured (COSY, HSQC). For practical reasons the assignment of H and C atoms in NMR characterizations reflects the numbering of chemical structures in the Supporting Information. A signal at 110 ppm was present in ^13^C spectra recorded at the Varian Inova spectrometer due to FM radio frequency interference and is indicated in the corresponding spectra. IR spectra were recorded with IRAffinity‐1S SHIMADZU system spectrophotometer. High Resolution Mass spectrometry (HRMS) was performed with an ESP‐MALDI‐FT‐ICR spectrometer equipped with a 7 T magnet (calibration of the instrument was done with Na trifluoroacetic acid (TFA) cluster ions) using Electrospray Ionization (ESI). ESI‐MS spectra were recorded with a Thermo Scientific LCQ fleet ion trap mass spectrometer. The preliminary DRA experiments of this work were conducted by M.G. Davighi and reported in her PhD Thesis [69].

### General procedure for DRA (*Route II*) from **1** [40] with Pd (OH)_2_/C to synthesize compounds **10**, **11**, and **12**

To a solution of dialdehyde **1** in MeOH (0.09 – 0.07 M), amine (**7** or **8** or **9**) was added under nitrogen atmosphere (with or without 3Å molecular sieves powder). The reaction mixture was stirred for 1 h ‐ 2 h under nitrogen atmosphere, then Pd(OH)_2_/C was added and left stirring under H_2_ atmosphere for 2 d ‐ 6 d, until ^1^H NMR control assessed the disappearance of aromatic protons and the presence of piperidine signals. When present, the molecular sieves were removed by filtration through Celite, and the filtrate was concentrated under vacuum. The residue was purified by flash chromatography affording the compound (**10** or **11** or **12**).

### Synthesis of compound **10**

Application of the general procedure to **1** (240 mg, 0.862 mmol) and ethylenediamine (24 µL, 0.359 mmol) in dry MeOH in the presence of 3Å molecular sieves and with 120 mg of Pd(OH)_2_/C furnished, after purification by column chromatography (CH_2_Cl_2_:MeOH:NH_4_(OH) (6%) 5:1:0.1), 93 mg of **10** (0.250 mmol, 69%) as a white waxy solid (*Rf* = 0.33, CH_2_Cl_2_:MeOH:NH_4_OH (6%) 10:1:0.1).

**10**: white waxy solid. ${\left[\alpha \right]}_{D}^{23}$ = ‐ 14.9 (c = 0.8, MeOH). ^1^H NMR (400 MHz, CD_3_OD): *δ* = 4.30‐4.25 (m, 2H, H‐3), 4.87‐4.82 (m, 2H, H‐4), 4.82‐4.77 (m, 2H, H‐5), 2.97 (dd, J = 2.6, 12.8 Hz, 2H, Ha‐2), 2.75 (dd, J = 2.8, 11.9 Hz, 2H, Ha‐6), 2.59 (br s, 4H, H‐7), 2.56 (dd, J = 3.3, 12.9 Hz, 2H, Hb‐2), 2.19‐2.11 (m, 2H, Hb‐6), 1.48 (s, 6H, Me), 1.33 (s, 6H, Me). ^13^C NMR (100 MHz, CD_3_OD): *δ* = 108.8 (s, 2C, OC(CH_3_)_2_), 78.5 (d, 2C, C‐4), 73.0 (d, 2C, C‐3), 68.8 (d, 2C, C‐5), 56.3 (t, 2C, C‐6), 54.2 (t, 2C, C‐7), 54.0 (t, 2C, C‐2), 27.1 (q, 2C, Me), 25.2 (q, 2C, Me). IR (CHCl_3_): *ṽ* = 3345, 2990, 2938, 2630, 1595, 1564, 1462, 1379, 1244, 1221 cm^−1^. MS (ESI) m/z (%): 373.26 (100) [M+H]^+^, 395.31 (71) [M+Na]^+^. C_18_H_32_N_2_O_6_ (372.46): calcd C, 58.05; H, 8.66; N, 7.52; found C, 58.25; H, 8.42; N, 7.77.

Procedure for DRA (Route I) from **2** with NaBH_3_CN to synthesize compound **10** is reported in the Supporting Information.

### Synthesis of compound **11**

Application of the general procedure to **1** (200 mg, 0.719 mmol) and hexamethylenediamine (42 mg, 0.361 mmol) in MeOH with 100 mg of Pd(OH)_2_/C furnished, after purification by column chromatography (gradient eluent from EtOAc:MeOH:NH_4_OH (6%) 10:1:0.1 to EtOAc:MeOH:NH_4_OH (6%) 5:1:0.1), 122 mg of **11** (0.285 mmol, 79%) as a white solid (*Rf* = 0.2, EtOAc:MeOH 10:1).

**11**: white solid. M.p. = 110.6‐111.8 °C. ${\left[\alpha \right]}_{D}^{22}$ = ‐ 47.3 (c = 1, MeOH). ^1^H NMR (400 MHz, CD_3_OD): *δ* = 4.32‐4.25 (m, 2H, H‐3), 3.86‐3‐76 (m, 4H, H‐4, H‐5), 3.02 (d, J = 12.7 Hz, 2H, Ha‐2), 2.78‐2.69 (m, 2H, Ha‐6), 2.47‐2.33 (m, 6H, H‐7, Hb‐2), 2.05‐1.97 (m, 2H, Hb‐6), 1.59‐1.51 (m, 4H, H‐8) 1.49 (s, 6H, Me), 1.42‐ 1‐28 (m, 4H, H‐9), 1.34 (s, 6H, Me). ^13^C NMR (100 MHz, CD_3_OD): *δ =* 110.1 (s, 2C, OC(CH_3_)_2_), 80.3 (d, 2C, C‐4), 74.3 (d, 2C, C‐3), 70.5 (d, 2C, C‐5), 59.2 (t, 2C, C‐7), 57.8 (t, 2C, C‐6), 55.1 (t, 2C, C‐2), 28.5 (q, 2C, Me), 28.6 (t, 2C, C‐9), 28.4 (t, 2C, C‐8), 27.4 (q, 2C, Me). IR (CHCl_3_): *ṽ* = 3684, 3599, 3480, 3032, 2940, 2826, 1603, 1464, 1404, 1379, 1238, 1202, 1142 cm^−1^. MS (ESI) m/z (%) = 429.21 (100) [M+H]^+^, 451.20 (16) [M+Na]^+^. C_22_H_40_N_2_O_6_ (428.56): calcd C, 61.66; H, 9.41; N, 6.54; found C, 61.88; H, 9.31; N, 6.32.

### Synthesis of compound **12**

Application of the general procedure to **1** (150 mg, 0.539 mmol) and decamethylenediamine **9** (47 mg, 0.270 mmol) in MeOH with 75 mg of Pd(OH)_2_/C furnished, after purification by column chromatography (gradient eluent from CH_2_Cl_2_:MeOH:NH_4_OH (6%) 20:1:0.1 to 10:1:0.1), 103 mg of **12** (0.213 mmol, 79%) as white solid (*Rf* = 0.3, CH_2_Cl_2_:MeOH:NH_4_OH (6%) 20:1:0.1).

**12**: white solid. M.p. = 111.9 – 112.5 °C. ${\left[\alpha \right]}_{D}^{22}$ = ‐ 45.0 (c = 0.6, MeOH). ^1^H NMR (400 MHz, CD_3_OD): *δ* = 4.30‐4.27 (m, 2H, H‐3), 3.84‐3‐78 (m, 4H, H‐4, H‐5), 3.04‐3.00 (m, 2H, Ha‐2), 2.75‐2.72 (m, 2H, Ha‐6), 2.24‐2.36 (m, 6H, H‐7, Hb‐2), 2.00 (dd, J = 11.3, 8.9 Hz, 2H, Hb‐6), 1.55‐1.52 (m, 4H, H‐8) 1.49 (s, 6H, Me), 1.34 (s, 6H, Me), 1.33 (br s, 12H, from H‐9 to H‐11). ^13^C NMR (100 MHz, CD_3_OD): *δ* = 110.2 (s, 2C, OC(CH_3_)_2_), 80.3 (d, 2C, C‐4), 74.6 (d, 2C, C‐3), 70.6 (d, 2C, C‐5), 59.3 (t, 2C, C‐7), 57.8 (t, 2C, C‐6), 55.1 (t, 2C, C‐2), 30.6 (t, 2C, H‐9 or H‐10 or H‐11), 30.6 (t, 2C, H‐9 or H‐10 or H‐11), 28.6 (t, 2C, H‐8), 28.5 (q, 2C, Me), 27.5 (q, 2C, Me), 26.6 (t, 2C, H‐9 or H‐10 or H‐11). IR (neat) ν = 3499, 2918, 2851, 1215, 1048 cm^−1^. MS (ESI) m/z (%) = 485.33 (100) [M+H]^+^, 243.08 (22) [M+2H]^2+^. C_26_H_48_N_2_O_6_ (484.68): calcd C, 64.43; H, 9.98; N, 5.78; found C, 64.47; H,9.77; N, 5.68.

### Synthesis of compound **18**

A solution of dialdehyde **2** (100 mg, 0.531 mmol) and *m*‐xylylenediamine (34 mg, 0.250 mmol) in dry MeOH (8 mL) was stirred for 18 h at room temperature under nitrogen atmosphere. Then, NaBH_3_CN (96 mg, 1.53 mmol) and AcOH (58 µL, 1.01 mmol) were added and the reaction mixture was stirred at room temperature for 4 days until a control by ^1^H NMR spectroscopy attested the presence of **18** as acetate salt. The mixture was filtered through Celite and the solvent was removed under reduced pressure. The corresponding free amine was obtained by dissolving the residue in MeOH, then the strongly basic resin Ambersep 900‐OH was added, and the mixture was stirred for 2 h. The resin was removed by filtration and the crude product was purified by flash chromatography on silica gel (gradient eluent from CH_2_Cl_2_:MeOH:NH_4_OH (6%) 15:1:0.1 to 5:1:0.1) to give 18 mg of **18** (0.0616 mmol, 24%) as a pale‐yellow oil (*Rf* = 0.1 CH_2_Cl_2_:MeOH:NH_4_OH (6%) 10:1:0.1).

**18**: pale‐yellow oil. ${\left[\alpha \right]}_{D}^{23}$ = ‐ 23.0 (c = 0.5, MeOH). ^1^H NMR (400 MHz, CD_3_OD): *δ* = 7.36‐7.23 (m, 4H, Ar), 4.30‐4.25 (m, 1H, H‐3), 3.87‐3.77 (m, 4H, H‐4, H‐5, H‐7), 3.62‐3.52 (m, 2H, CH_2_NH_2_), 2.91 (br d, J = 14.4 Hz, 1H, Ha‐2), 2.72‐2.65 (m, 1H, Ha‐6), 2.50 (dd, J = 3.7, 12.9 Hz, 1H, Hb‐2), 2.11‐1.99 (m, 1H, Hb‐6), 1.49 (s, 3H, Me), 1.33 (s, 3H, Me). ^13^C NMR (50 MHz, CD_3_OD): *δ* = 142.7 (s, 1C, Ar), 139.0 (s, 1C, Ar), 129.7 (d, 1C, Ar), 129.6 (d, 1C, Ar), 129.3 (d, 1C, Ar), 127.6 (d, 1C, Ar), 110.2 (s, 1C, OC(CH_3_)_2_), 80.2 (d, 1C, C‐4), 74.6 (d, 1C, C‐3), 70.5 (d, 1C, C‐5), 63.0 (t, 1C, C‐7), 57.3 (t, 1C, C‐6), 55.0 (t, 1C, C‐2), 46.4 (t, 1C, CH_2_NH_2_), 28.6 (q, 1C, Me), 26.6 (q, 1C, Me). MS (ESI) m/z (%) = 293.09 (100) [M+H]^+^. C_16_H_24_N_2_O_3_ (292.37): calcd C, 65.73; H, 8.27; N, 9.58; found C, 65.88; H, 8.25; N, 9.80.

### General procedure for CuAAC reaction to synthesize intermediates **23**, **28**, **34**, and **40**

To a solution of **22** [51] (appropriate equivalent) in a 2:1 THF:H_2_O (0.05 M) mixture and alkyne (**20**, **27**, **33**, or **38**; 1 equiv.), CuSO_4_ (0.3 equiv) and sodium ascorbate (0.6 equiv) were added. The reaction mixture was stirred in a MW reactor at 80°C for 45‐75 min until TLC analysis showed the disappearance of the starting material (PE:EtOAc appropriate ratio) and formation of the desired product. After filtration through Celite, the solvent was removed under reduced pressure and the crude was purified through FCC on silica gel to obtain the multivalent adducts (**23**, **28**, **34** and **40**).

### Synthesis of compound **23**

The general procedure employing scaffold **20** (20 mg, 0.159 mmol), and azide **22** (90 mg, 0.333 mmol) afforded 79 mg of **23** (0.118 mmol, 75%) as a white solid after purification by FCC on silica gel (CH_2_Cl_2_:MeOH 50:1; *Rf* = 0.11).

**23**: white solid. M. p. = 110.1‐111.3 °C. ^1^H NMR (400 MHz, CDCl_3_): *δ* = 8.27 (br s, 1H, Ar), 7.85 (s, 2H, triazole), 7.80 (d, J = 7.7 Hz, 2H, Ar), 7.44 (t, J = 7.7 Hz, 1H, Ar), 4.57 (br s, 2H, NH), 4.37 (t, J = 7.0 Hz, 4H, CH_2_‐triazole), 3.17‐2.94 (m, 4H, CH_2_NH), 1.98‐1.83 (m, 4H, CH_2_CH_2_‐triazole), 1.47‐1.37 (m, 22H, CH_2_CH_2_‐NH, OC(CH_3_)_3_), 1.34‐1.22 (m, 16H). ^13^C NMR (100 MHz, CDCl_3_): *δ* = 156.1 (s, 2C, NCOO), 147.4 (s, 2C, triazole), 131.3 (s, 2C, Ar), 129.5 (d, 1C, Ar), 125.3 (d, 2C, Ar), 122.9 (d, 1C, Ar), 119.9 (d, 2C, triazole), 79.0 (s, 2C, OC(CH_3_)_3_), 50.5 (t, 2C, CH_2_‐triazole), 40.6 (t, 2C, CH_2_NH), 30.3 (t, 2C, CH_2_CH_2_‐triazole), 30.1 (t, 2C, CH_2_CH_2_‐NH), 29.1 (2C), 29.0 (2C), 28.5 (q, 6C, OC(CH_3_)_3_), 26.7 (2C), 26.5 (2C). IR (CHCl_3_): *ṽ* = 3658, 3457, 3008, 2934, 2860, 1706, 1508, 1461, 1367, 1227, 1168, 1048, 918 cm^−1^. MS (ESI) m/z (%) = 689.33 (100) [M+Na]^+^. C_36_H_58_N_8_O_4_ (666.90): calcd C, 64.84; H, 8.77; N, 16.80; found C, 64.48; H, 8.81; N, 16.42.

### Synthesis of compound **28**

The general procedure employing scaffold **27** (20 mg, 0.159 mmol) and azide **22** (90 mg, 0.333 mmol) afforded 65 mg of **28** (0.0975 mmol, 61%) as a white solid after purification by FCC on silica gel (CH_2_Cl_2_:MeOH 50:1; *Rf* = 0.11).

**28**: white solid. M. p. = 176.3‐177.0 °C. ^1^H NMR (400 MHz, CDCl_3_): *δ* = 7.89 (s, 4H, Ar), 7.80 (s, 2H, triazole), 4.53 (br s, 2H, NH), 4.38 (t, J = 7.2 Hz, 4H, CH_2_‐triazole), 3.07 (br s, 4H, CH_2_NH), 2.02‐1.84 (m, 4H, CH_2_CH_2_‐triazole), 1.49‐1.39 (m, 22H, CH_2_CH_2_‐NH, OC(CH_3_)_3_), 1.37‐1.25 (m, 16H). ^13^C NMR (100 MHz, CDCl_3_): *δ* = 156.1 (s, 2C, NCOO), 147.3 (s, 2C, triazole), 130.4 (s, 2C, Ar), 126.2 (d, 4C, Ar), 119.7 (d, 2C, triazole), 79.1 (s, 2C, OC(CH_3_)_3_), 50.6 (t, 2C, CH_2_‐triazole), 40.6 (t, 2C, CH_2_NH), 30.4 (t, 2C, CH_2_CH_2_‐triazole), 30.1 (t, 2C, CH_2_CH_2_‐NH), 29.1 (2C), 29.0 (2C), 28.5 (q, 6C, OC(CH_3_)_3_), 26.7 (2C), 26.5 (2C). IR (CHCl_3_): *ṽ* = 3455, 2930, 2856, 1706, 1507, 1366, 1261, 1168, 1098, 1015, 936 cm^−1^. MS (ESI) m/z (%) = 689.33 (100) [M+Na]^+^. C_36_H_58_N_8_O_4_ (666.90): calcd C, 64.84; H, 8.77; N, 16.80; found C, 64.76; H, 8.93; N, 16.51.

### Synthesis of compound **34**

The general procedure employing scaffold **33** (19 mg, 0.127 mmol) and azide **22** (110 mg, 0.407 mmol) afforded 100 mg of **34** (0.104 mmol, 82%) as a white waxy solid after purification by FCC on silica gel (gradient eluent from CH_2_Cl_2_:MeOH 100:1 to CH_2_Cl_2_:MeOH:NH_4_OH (6%); *Rf* = 0.2 in CH_2_Cl_2_:MeOH:NH_4_OH (6%) 50:1:0.1).

**34**: white waxy solid. ^1^H NMR (400 MHz, CDCl_3_): *δ* = 8.29 (s, 3H, Ar), 7.95 (s, 3H, triazole), 4.56 (br s, 3H, NH), 4.40 (t, J = 6.9 Hz, 6H, CH_2_‐triazole), 3.15‐2.98 (m, 6H, CH_2_‐NH), 2.00‐1.87 (m, 6H, CH_2_CH_2_‐triazole), 1.48‐1.39 (m, 33H, CH_2_CH_2_‐NH, OC(CH_3_)_3_), 1.36‐1.24 (m, 24H). ^13^C NMR (50 MHz, CD_3_OD): *δ* = 156.1 (s, 3C, NCOO), 147.2 (s, 3C, triazole), 131.9 (s, 3C, Ar), 122.3 (d, 3C, Ar), 120.3 (d, 3C, triazole), 79.1 (s, 3C, OC(CH_3_)_3_), 50.6 (t, 3C, CH_2_‐triazole), 40.6 (t, 3C, CH_2_NH), 30.3 (t, 3C, CH_2_CH_2_‐triazole), 30.1 (t, 3C, CH_2_CH_2_‐NH), 29.1 (3C), 29.0 (3C), 28.5 (q, 9C, OC(CH_3_)_3_), 26.7 (3C), 26.5 (3C). IR (CHCl_3_): *ṽ* = 3457, 2979, 2934, 2859, 1706, 1508, 1463, 1396, 1367, 1259, 1168, 1097, 1015 cm^−1^. MS (ESI) m/z (%) = 983.50 (100) [M+Na]^+^. C_51_H_84_N_12_O_6_ (961.29): calcd C, 63.72; H, 8.81; N, 17.48; found C, 63.74; H, 8.78; N, 17.11.

### Synthesis of compound **40**

The general procedure employing scaffold **38** (103 µL, 0.938 mmol) and azide **22** (230 mg, 0.851 mmol) afforded 282 mg of **40** (0.757 mmol, 89%) as a white solid after purification by FCC on silica gel (PE:EtOAc 3:1; *Rf* = 0.11).

**40**: white solid. M.p. = 84.5‐86.2 °C. ^1^H NMR (400 MHz, CDCl_3_): *δ* = 7.83 (d, J = 8.1 Hz, 2H, Ar), 7.75 (s, 1H, triazole), 7.42 (t, J = 7.7 Hz, 2H, Ar), 7.36‐7.29 (m, 1H, Ar), 4.51 (br s, 1H, NH), 4.39 (t, J = 7.2 Hz, 2H, CH_2_‐triazole), 3.17‐2.97 (m, 2H, CH_2_‐NH), 2.01‐1.86 (m, 2H, CH_2_CH_2_‐triazole), 1.51‐1.39 (m, 11H, CH_2_CH_2_‐NH, OC(CH_3_)_3_), 1.38‐1.23 (m, 8H). ^13^C NMR (50 MHz, CDCl_3_): *δ* = 156.0 (s, 1C, NCOO), 147.6 (s, 1C, triazole), 130.7 (s, 1C, Ar), 128.8 (d, 2C, Ar), 128.0 (d, 1C, Ar), 125.6 (d, 2C, Ar), 119.5 (d, 1C, triazole), 78.9 (s, 1C, OC(CH_3_)_3_), 50.3 (t, 1C, CH_2_‐triazole), 40.5 (t, 1C, CH_2_NH), 30.3, 30.0, 29.0, 28.9, 28.4 (q, 3C, OC(CH_3_)_3_), 26.6, 26.4. IR (CHCl_3_): *ṽ* = 3455, 3007, 2932, 2857, 1707, 1508, 1368, 1244, 1167, 972, 937 cm^−1^. MS (ESI) m/z (%) = 395.12 (57) [M+Na]^+^, 766.89 (100) [2M+Na]^+^. Anal. Calcd for C_21_H_32_N_4_O_2_: C, 67.71; H, 8.66; N, 15.04. Found: C, 67.54; H, 8.48; N, 14.71.

### Synthesis of compound **39**

The general procedure employing propargyl alcohol **6** (64 µL, 1.11 mmol) and azide **22** (250 mg, 0.925 mmol) afforded 255 mg of **39** (0.781 mmol, 84%) as a white solid after purification by FCC on silica gel (CH_2_Cl_2_:MeOH:NH_4_OH (6%) 20:1:0.1; *Rf* = 0.25).

**39**: white solid. M. p. 79.2‐80.2 °C. ^1^H NMR (400 MHz, CDCl_3_): *δ* = 7.52 (s, 1H, triazole), 4.83‐4.74 (m, 2H, CH_2_OH), 4.61‐4.48 (br s, 1H, NH), 4.33 (t, J = 7.2 Hz, 2H, CH_2_‐triazole), 3.15‐2.91 (m, 3H, CH_2_NH, OH), 1.93‐1.84 (m, 2H, CH_2_CH_2_‐triazole), 1.50‐1.35 (m, 11H, CH_2_CH_2_‐NH, OC(CH_3_)_3_), 1.33‐1.22 (m, 8H). ^13^C NMR (50 MHz, CDCl_3_): *δ* = 156.1 (s, 1C, NCOO), 148.0 (s, 1C, triazole), 121.7 (d, 1C, triazole), 79.1 (s, 1C, OC(CH_3_)_3_), 56.2 (t, 1C, CH_2_‐OH), 50.3 (t, 1C, CH_2_‐triazole), 40.5 (t, 1C, CH_2_NH), 30.2, 29.9, 28.9, 28.8, 28.4 (q, 3C, OC(CH_3_)_3_), 26.6, 26.3. IR (CHCl_3_) ν = 3748, 3593, 3454, 3007, 2932, 2859, 1705, 1508, 1368, 1275, 1234, 1167, 1045 cm^−1^. MS (ESI) m/z (%) = 349.14 (100) [M+Na]^+^, 674.91 (76) [2M+Na]^+^. Anal. Calcd for C_16_H_30_N_4_O_3_: C, 58.87; H, 9.26; N, 17.16. Found: C, 58.99; H, 9.13; N, 17.03.

### Synthesis of compound **29**

A solution of **28** (45 mg, 0.0675 mmol) in dry CH_2_Cl_2_ (1.5 mL) was left stirring with TFA (237 µL, 3.08 mmol) at room temperature for 4 h until the disappearance of starting material **28** was assessed by a control *via* 1H NMR. Then, the crude mixture was concentrated under vacuum. The corresponding free amine was obtained by dissolving the residue in MeOH, then NH_4_OH (32%) (20 µL) was added, and the mixture was stirred for 20 min. The mixture was concentrated under vacuum to afford 31 mg of **29** (0.0664 mmol) as a colorless oil, pure enough to be employed in the subsequent double reductive amination step.

### General procedure for deprotection of Boc to obtain compound **24**, **35**, **41**, and **42**

To a solution of compound (**23**, **34**, **39**, and **40**) in dry CH_2_Cl_2_ (0.09 M) was left stirring with TFA at room temperature for 4‐6 h. The disappearance of starting material was assessed by a control *via* ^1^H NMR. Then, the crude mixture was concentrated under vacuum. The corresponding free amine was obtained by dissolving the residue in MeOH, then the strongly basic resin Ambersep 900‐OH was added and the mixture was stirred for 2 h. The resin was removed by filtration and the crude product, if necessary, was purified on silica gel by flash column chromatography to afford compound as free base (**24**, **35**, **41**, and **42**).

### Synthesis of compound **24**

Following the general procedure TFA (572 µL, 7.48 mmol) was added to a CH_2_Cl_2_ solution of **23** (113 mg, 0.169 mmol) and furnished, after treatment with Ambersep 900‐OH, 80 mg of **24** (0.171 mmol) as a colorless oil, pure enough to be employed in the subsequent double reductive amination step.

### Synthesis of compound **35**

Following the general procedure TFA (493 µL, 6.45 mmol) was added to a CH_2_Cl_2_ solution of **34** (93 mg, 0.0978 mmol) and furnished, after treatment with Ambersep 900‐OH, 64 mg of **35** (0.0968 mmol) as a colorless oil, pure enough to be employed in the subsequent double reductive amination step.

### Synthesis of compound **41**

Following the general procedure TFA (1.14 mL, 14.9 mmol) was added to a CH_2_Cl_2_ solution of **39** (222 mg, 0.680 mmol) and furnished, after treatment with Ambersep 900‐OH and purification by column chromatography (CH_2_Cl_2_:MeOH:NH_4_OH (6%) 10:1:0.1), 93 mg of **41** (0.411 mmol, 60%) as a white solid (*Rf* = 0.05, CH_2_Cl_2_:MeOH:NH_4_OH (6%) 3:1:0.1).

**41**: white solid. M.p. = 86.6‐88.2 °C. ^1^H NMR (400 MHz, CD_3_OD): *δ* = 7.91 (s, 1H, triazole), 4.67 (s, 2H, CH_2_OH), 4.40 (t, J = 9.0, 2H, CH_2_‐triazole), 2.63 (t, J = 7.2 Hz, 2H, CH_2_‐NH), 1.90 (quint, J = 7.2 Hz, 2H, CH_2_CH_2_‐triazole), 1.53‐1.41 (m, 2H, CH_2_CH_2_‐NH), 1.40‐1.23 (m, 8H). ^13^C NMR (50 MHz, CD_3_OD): *δ* = 149.0 (s, 1C, triazole), 124.0 (d, 1C, triazole), 56.4 (t, 1C, CH_2_‐OH), 51.3 (t, 1C, CH_2_‐triazole), 42.4 (t, 1C, CH_2_NH), 33.5 (t, 1C, CH_2_CH_2_‐triazole), 31.3, 30.4, 30.0, 27.8, 27.4. IR (CHCl_3_): *ṽ* = 3013, 2930, 2857, 924, 918 cm^−1^. MS (ESI) m/z (%) = 227.11 (100) [M+H]^+^. Anal. Calcd for C_11_H_22_N_4_O: C, 58.38; H, 9.80; N, 24.76. Found: C, 58.44; H, 9.69; N, 25.00.

### Synthesis of compound **42**

Following the general procedure TFA (226 µL, 2.95 mmol) was added to a CH_2_Cl_2_ solution of **40** (50 mg, 0.134 mmol) and furnished, after treatment with Ambersep 900‐OH, 37 mg of **42** (0.136 mmol, quantitative) as a white solid.

**42**: white solid. M.p. 121.3‐122.3 °C. ^1^H NMR (400 MHz, CD_3_OD): *δ* = 8.33 (s, 1H, triazole), 7.81 (d, J = 7.4 Hz, 2H, Ar), 7.43 (t, J = 7.4 Hz, 2H, Ar), 7.37‐7.30 (m, 1H, Ar), 4.43 (t, J = 7.1 Hz, 2H, CH_2_‐triazole), 2.69 (t, J = 7.4 Hz, 2H, CH_2_‐NH), 2.01‐1.88 (m, 2H, CH_2_CH_2_‐triazole), 1.56‐1.45 (m, 2H, CH_2_CH_2_‐NH), 1.39‐1.29 (m, 8H). ^13^C NMR (50 MHz, CD_3_OD): *δ* = 148.8 (s, 1C, triazole), 131.8 (s, 1C, Ar), 130.0 (d, 2C, Ar), 129.3 (d, 1C, Ar), 126.6 (d, 2C, Ar), 122.2 (d, 1C, triazole), 51.4 (t, 1C, CH_2_‐triazole), 41.9 (t, 1C, CH_2_NH), 31.9 (t, 1C, CH_2_CH_2_NH), 31.2 (t, 1C, CH_2_CH_2_‐triazole), 30.2, 29.9, 27.6, 27.4. IR (CHCl_3_): *ṽ* = 3026, 2928, 2857, 1261, 1225, 1204, 1096, 1013 cm^−1^. MS (ESI) m/z (%) = 273.15 (100) [M+H]^+^, 544.89 (19) [2M+H]^+^. Anal. Calcd for C_16_H_24_N_4_: C, 70.55; H, 8.88; N, 20.57. Found: C, 70.32; H, 8.98; N, 20.33.

### General procedure for DRA (*Route I*) from **2** [40] with NaBH_3_CN to synthesize compounds **25**, **30**, **36**, **43**, and **44**

A solution of dialdehyde **2** (appropriate equivalent) and amine (**24**, **29**, **35**, **41**, or **42**) in a mixture of MeOH and CHCl_3_ was stirred for 2 h at reflux. Then, NaBH_3_CN (3 equiv.) and AcOH (2 equiv.) were added and the reaction mixture was stirred at reflux for 2‐3 days until a control by ^1^H NMR spectroscopy attested the presence of product as acetate salt. The solvent was removed under reduced pressure. The corresponding free amine was obtained by dissolving the residue in MeOH, then the strongly basic resin Ambersep 900‐OH was added, and the mixture was stirred for 4 h. The resin was removed by filtration and the crude product was purified by flash chromatography on silica gel to give compound as free amine (**25**, **30**, **36**, **43**, or **44**).

### Synthesis of compound **25**

Application of the general procedure to **2** (68 mg, 0.361 mmol) and diamine **24** (80 mg, 0.171 mmol) in a mixture of MeOH (23 mL) and CHCl_3_ (3.2 mL) furnished, after Ambersep 900‐OH and purification by column chromatography (gradient eluent from CH_2_Cl_2_:MeOH:NH_4_OH (6%) 20:1:0.1 to 15:1:0.1), 49 mg of **25** (0.0629 mmol, 37%) as a white waxy solid (*Rf* = 0.35 CH_2_Cl_2_:MeOH:NH_4_OH (6%) 10:1:0.1).

**25**: white waxy solid. ${\left[\alpha \right]}_{D}^{24}$ = + 9.17 (c = 0.6, CHCl_3_). ^1^H NMR (400 MHz, CD_3_OD): *δ* = 8.41 (s, 2H, triazole), 8.31‐8.27 (m, 1H, Ar), 7.83 (dd, J = 1.6, 7.8 Hz, 2H, Ar), 7.53 (t, J = 7.8 Hz, 1H, Ar), 4.47 (t, J = 7.0 Hz, 4H, H‐14), 4.26 (dt, J = 3.8, 4.4 Hz, 2H, H‐3), 3.85‐3.74 (m, 4H, H‐4, H‐5), 3.04‐2.96 (m, 2H, Ha‐2), 2.77‐2.67 (m, 2H, Ha‐6), 2.43‐2.28 (m, 6H, Hb‐2, H‐7), 2.05‐1.91 (m, 6H, Hb‐6, H‐13), 1.55‐1.46 (m, 4H, H‐8), 1.48 (s, 6H, Me), 1.40‐1.26 (m, 16H, H‐9, H‐10, H‐11, H‐12), 1.32 (s, 6H, Me). ^13^C NMR (50 MHz, CD_3_OD): *δ* = 148.4 (s, 2C, triazole), 132.6 (s, 2C, Ar), 130.7 (d, 1C, Ar), 126.4 (d, 2C, Ar), 123.8 (d, 1C, Ar), 122.4 (d, 2C, triazole), 110.2 (s, 2C, OC(CH_3_)_2_), 80.3 (d, 2C, C‐4), 74.6 (d, 2C, C‐3), 70.5 (d, 2C, C‐5), 59.2 (t, 2C, C‐7), 57.7 (t, 2C, C‐6), 55.1 (t, 2C, C‐2), 51.5 (t, 2C, C‐14), 31.2, 30.3, 29.9, 28.5, 28.4, 27.4, 26.6 (t, 12C, C‐8, C‐9, C‐10, C‐11, C‐12, C‐13 and q, 4C, Me). IR (CHCl_3_): *ṽ* = 3846, 3742, 3673, 3650, 2995, 2935, 2856, 1542, 1464, 1378, 1260, 1058, 1017, 941 cm^−1^. MS (ESI) m/z (%) = 779.67 (100) [M+H]^+^. C_42_H_66_N_8_O_6_ (779.02): calcd C, 64.75; H, 8.54; N, 14.38; found C, 64.53; H, 8.67; N, 14.08.

### Synthesis of compound **30**

Application of the general procedure to **2** (26 mg, 0.138 mmol) and diamine **29** (31 mg, 0.0664 mmol) in a mixture of MeOH (9 mL) and CHCl_3_ (1.2 mL) furnished, after Ambersep 900‐OH and purification by column chromatography (CH_2_Cl_2_:MeOH:NH_4_OH (6%) 25:1:0.1), 22 mg of **30** (0.0282 mmol, 43%) as a white waxy solid (*Rf* = 0.33 CH_2_Cl_2_:MeOH:NH_4_OH (6%) 10:1:0.1).

**30**: white waxy solid. ${\left[\alpha \right]}_{D}^{25}$ = + 8.33 (c = 0.6, CHCl_3_). ^1^H NMR (400 MHz, CD_3_OD): *δ* = 8.38 (s, 2H, triazole), 7.91 (s, 4H, Ar), 4.46 (t, J = 7.0 Hz, 4H, H‐14), 4.26 (dt, J = 3.6, 4.0 Hz, 2H, H‐3), 3.86‐3.75 (m, 4H, H‐4, H‐5), 3.00 (br d, J = 13.1 Hz, 2H, Ha‐2), 2.76‐2.68 (m, 2H, Ha‐6), 2.44‐2.31 (m, 6H, Hb‐2, H‐7), 2.03‐1.92 (m, 6H, Hb‐6, H‐13), 1.55‐1.46 (m, 4H, H‐8), 1.48 (s, 6H, Me), 1.42‐1.24 (m, 16H, H‐9, H‐10, H‐11, H‐12), 1.32 (s, 6H, Me). ^13^C NMR (100 MHz, CD_3_OD): *δ* = 148.3 (s, 2C, triazole), 131.7 (s, 2C, Ar), 127.2 (d, 4C, Ar), 122.3 (d, 2C, triazole), 110.2 (s, 2C, OC(CH_3_)_2_), 80.3 (d, 2C, C‐4), 74.5 (d, 2C, C‐3), 70.5 (d, 2C, C‐5), 59.2 (t, 2C, C‐7), 57.7 (t, 2C, C‐6), 55.0 (t, 2C, C‐2), 51.5 (t, 2C, C‐14), 31.2, 30.3, 29.9, 28.5, 28.4, 27.4, 26.6 (t, 12C, C‐8, C‐9, C‐10, C‐11, C‐12, C‐13 and q, 4C, Me). IR (CHCl_3_): *ṽ* = 3862, 3743, 3673, 2994, 2932, 2858, 1712, 1515, 1456, 1368, 1261, 1098, 1017 cm^−1^. MS (ESI) m/z (%) = 390.25 (100) [M+2H]^2+^, 779.02 [M+H^+^]. HRMS (ESP+): *m/z* calcd for C_42_H_66_N_8_O_6_: 779.51781 [M+H]^+^; found: 779.51769.

### Synthesis of compound **36**

Application of the general procedure to **2** (62 mg, 0.329 mmol) and triamine **35** (70 mg, 0.106 mmol) in a mixture of MeOH (15 mL) and CHCl_3_ (2.1 mL) furnished, after Ambersep 900‐OH and purification by column chromatography (CH_2_Cl_2_:MeOH:NH_4_OH (6%) 20:1:0.1), 42 mg of **36** (0.0372 mmol, 35%) as a white waxy solid (*Rf* = 0.28 CH_2_Cl_2_:MeOH:NH_4_OH (6%) 10:1:0.1).

**36**: white waxy solid. ${\left[\alpha \right]}_{D}^{24}$ = + 5.82 (c = 0.55, CHCl_3_). ^1^H NMR (400 MHz, CD_3_OD): *δ* = 8.45 (s, 3H, triazole), 8.28 (s, 3H, Ar), 4.46 (t, J = 7.0 Hz, 6H, H‐14), 4.24 (dt, J = 3.7, 4.2 Hz, 3H, H‐3), 3.83‐3.73 (m, 6H, H‐4, H‐5), 2.97 (br d, J = 12.5 Hz, 3H, Ha‐2), 2.73‐2.65 (m, 3H, Ha‐6), 2.40‐2.26 (m, 9H, Hb‐2, H‐7), 2.01‐1.90 (m, 9H, Hb‐6, H‐13), 1.52‐1.42 (m, 6H, H‐8), 1.46 (s, 9H, Me), 1.39‐1.21 (m, 24H, H‐9, H‐10, H‐11, H‐12), 1.31 (s, 9H, Me). ^13^C NMR (100 MHz, CD_3_OD): *δ* = 148.0 (s, 3C, triazole), 133.4 (s, 3C, Ar), 123.2 (d, 3C, Ar), 122.7 (d, 3C, triazole), 111.4 (interference), 110.1 (s, 3C, OC(CH_3_)_2_), 80.3 (d, 3C, C‐4), 74.5 (d, 3C, C‐3), 70.5 (d, 3C, C‐5), 59.2 (t, 3C, C‐7), 57.8 (t, 3C, C‐6), 55.0 (t, 3C, C‐2), 51.6 (t, 3C, C‐14), 31.2, 30.3, 30.0, 28.5, 28.4, 27.4, 26.6 (t, 18C, C‐8, C‐9, C‐10, C‐11, C‐12, C‐13 and q, 6C, Me). IR (CHCl_3_): *ṽ* = 3743, 3681, 3029, 2936, 2859, 1606, 1515, 1464, 1379, 1260, 1238, 1058, 1016, 931 cm^−1^. MS (ESI) m/z (%) = 565.42 (46) [M+3H]^3+^, 1129.58 (100) [M+H]^+^. C_60_H_96_N_12_O_9_ (1129.48): calcd C, 63.80; H, 8.57; N, 14.88; found C, 63.72; H, 8.54; N, 14.53.

### Synthesis of compound **43**

Application of the general procedure to **2** (42 mg, 0.223 mmol) and amine **41** (50 mg, 0.221 mmol) in a mixture of MeOH (30 mL) and CHCl_3_ (4 mL) furnished, after Ambersep 900‐OH and purification by column chromatography (CH_2_Cl_2_:MeOH:NH_4_OH (6%) 10:1:0.1), 40 mg of **43** (0.105 mmol, 47%) as a pale‐yellow oil (*Rf* = 0.26, CH_2_Cl_2_:MeOH:NH_4_OH (6%) 10:1:0.1).

**43**: pale yellow‐oil. ${\left[\alpha \right]}_{D}^{23}$ = + 5.11 (c = 0.9, CHCl_3_). ^1^H NMR (400 MHz, CD_3_OD): *δ* = 7.90 (s, 1H, triazole), 4.67 (s, 2H, H‐15), 4.39 (t, J = 7.0 Hz, 2H, H‐14), 4.32‐4.25 (m, 1H, H‐3), 3.86‐3.76 (m, 2H, H‐4, H‐5), 3.01 (br d, J = 13.1 Hz, 1H, Ha‐2), 2.77‐2.69 (m, 1H, Ha‐6), 2.47‐2.31 (m, 3H, Hb‐2, H‐7), 2.04‐1.96 (m, 1H, Hb‐6), 1.90 (quint, J = 7.1 Hz, 2H, H‐13), 1.56‐1.45 (m, 5H, H‐8, Me), 1.40‐1.26 (m, 11H, H‐9, H‐10, H‐11, H‐12, Me). ^13^C NMR (100 MHz, CD_3_OD): *δ* = 149.0 (s, 1C, triazole), 124.0 (d, 1C, triazole), 110.2 (s, 1C, OC(CH_3_)_2_), 80.3 (d, 1C, C‐4), 74.5 (d, 1C, C‐3), 70.5 (d, 1C, C‐5), 59.2 (t, 1C, C‐7), 57.7 (t, 1C, C‐6), 56.5 (t, 1C, C‐2), 55.1 (t, 1C, C‐15), 51.3 (t, 1C, C‐14), 31.3 (t, 1C, C‐13), 30.3, 29.9, 28.5, 28.4, 27.4, 27.4, 26.6 (t, 5C, C‐8, C‐9, C‐10, C‐11, C‐12 and q, 2C, 2xMe). IR (CHCl_3_): *ṽ* = 3672, 3599, 3005, 2936, 2859, 1464, 1379, 1236, 1142, 1057, 930 cm^−1^. MS (ESI) m/z (%) = 383.23 (100) [M+H]^+^, 405.24 (21) [M+Na]^+^. Anal. Calcd for C_19_H_34_N_4_O_4_: C, 59.66; H, 8.96; N, 14.65. Found: C, 59.43; H, 8.91; N, 14.28.

Procedure for DRA (*Route II*) from **2** with NaBH_3_CN to synthesize compound **43** is reported in the Supporting Information.

### Synthesis of compound **44**

Application of the general procedure to **2** (35 mg, 0.186 mmol) and amine **42** (50 mg, 0.184 mmol) in a mixture of MeOH (25 mL) and CHCl_3_ (3.3 mL) furnished, after Ambersep 900‐OH and purification by column chromatography (CH_2_Cl_2_:MeOH:NH_4_OH (6%) 30:1:0.1), 34 mg of **44** (0.0793 mmol, 43%) as a white solid (*Rf* = 0.14, CH_2_Cl_2_:MeOH:NH_4_OH (6%) 30:1:0.1).

**44**: white solid. M.p. = 90.5‐92.1 °C. ${\left[\alpha \right]}_{D}^{23}$ = + 9.2 (c = 0.5, CHCl_3_). ^1^H NMR (400 MHz, CD_3_OD): *δ* = 8.31 (s, 1H, triazole), 7.82 (d, J = 7.4 Hz, 2H, Ar), 7.42 (t, J = 7.4 Hz, 2H, Ar), 7.37‐7.30 (m, 1H, Ar), 4.43 (t, J = 7.0, 2H, H‐14), 4.29‐4.23 (m, 1H, H‐3), 3.85‐3.76 (m, 2H, H‐4, H‐5), 2.98 (br d, J = 13.0 Hz, 1H, Ha‐2), 2.76‐2.67 (m, 1H, Ha‐6), 2.43‐2.28 (m, 3H, Hb‐2, H‐7), 2.03‐1.88 (m, 3H, Hb‐6, H‐13), 1.55‐1.43 (m, 5H, H‐8, Me), 1.39‐1.23 (m, 11H, H‐9, H‐10, H‐11, H‐12, Me). ^13^C NMR (100 MHz, CD_3_OD): *δ* = 148.8 (s, 1C, triazole), 131.8 (s, 1C, Ar), 130.0 (d, 2C, Ar), 129.3 (d, 1C, Ar), 126.6 (d, 2C, Ar), 122.1 (d, 1C, triazole), 110.2 (s, 1C, OC(CH_3_)_2_), 80.3 (d, 1C, C‐4), 74.5 (d, 1C, C‐3), 70.5 (d, 1C, C‐5), 59.2 (t, 1C, C‐7), 57.8 (t, 1C, C‐6), 55.0 (t, 1C, C‐2), 51.4 (t, 1C, C‐14), 31.2, 30.3, 29.9, 28.5, 28.4, 27.4, 26.6 (t, 6C, C‐8, C‐9, C‐10, C‐11, C‐12, C‐13 and q, 2C, 2xMe). IR (CHCl_3_): *ṽ* = 3672, 2994, 2936, 2857, 1464, 1379, 1244, 1213, 1059, cm^−1^. MS (ESI) m/z (%) = 429.94 (100) [M+H]^+^, 451.29 (10) [M+Na]^+^, 878.85 (15) [2M+Na] ^+^. Anal. Calcd for C_24_H_36_N_4_O_3_: C, 67.26; H, 8.47; N, 13.07. Found: C, 67.65; H, 8.13; N, 13.20.

### General procedure for acetonide deprotection to obtain compounds **13**, **14**, **15**, **19**, **26**, **37**, and **45**

A solution of compound (**10**, **11**, **12**, **18**, **25**, **36**, or **43**) in MeOH (0.05 M) was left stirring with 12 M HCl at room temperature for 16‐20 h. The crude mixture was concentrated to yield the hydrochloride salt of compound. The corresponding free amine was obtained by dissolving the residue in MeOH, then the strongly basic resin Ambersep 900‐OH was added, and the mixture was stirred for 40 min. The resin was removed by filtration and the crude product, if necessary, was purified on silica gel by FCC to afford compound as free base (**13**, **14**, **15**, **19**, **26**, **37**, or **45**).

### Synthesis of compound **13**

Following the general procedure 12 M HCl (60 µL) was added to a solution of **10** (15 mg, 0.0403 mmol) and furnished, after treatment with Ambersep 900‐OH, 10 mg of **13** (0.0342 mmol, 86 %) as a white waxy solid (*Rf* = 0.00 CH_2_Cl_2_:MeOH:NH_4_OH (6%) 10:1:0.1).

**13**: white waxy solid. ${\left[\alpha \right]}_{D}^{23}$ = ‐ 30.1, (c = 0.4, MeOH). ^1^H NMR (400 MHz, D_2_O): *δ* = 3.81‐3.77 (m, 2H, H‐3), 3.68‐3.61 (m, 2H, H‐5), 3.32‐3.26 (m, 2H, H‐4), 2.75‐2.60 (m, 4H, Ha‐2, Ha‐6), 2.41‐2.34 (m, 4H, H‐7), 2.14 (m, 2H, Hb‐2), 1.98‐1.87 (m, 2H, Hb‐6). ^13^C NMR (50 MHz, D_2_O): *δ* = 73.8 (d, 2C, C‐4), 67.6 (d, 2C, C‐3), 67.4 (d, 2C, C‐5), 56.7 (t, 2C, C‐2), 55.8 (t, 2C, C‐6), 53.3 (t, 2C, C‐7). MS (ESI) m/z (%) = 293.26 (100) [M+H]^+^, 315.26 (26) [M+Na]^+^. C_12_H_24_N_2_O_6_ (292.33): calcd C, 49.30; H, 8.28; N, 9.58; found C, 49.59; H, 8.45; N, 9.29.

### Synthesis of compound **14**

Following the general procedure 12 M HCl (60 µL) was added to a solution of **11** (20 mg, 0.0467 mmol) and furnished, after treatment with Ambersep 900‐OH, 16 mg of **14** (0.0459 mmol, 98%) as a pale‐yellow oil (*Rf* = 0.00 CH_2_Cl_2_:MeOH:NH_4_OH (6%) 10:1:0.1).

**14**: pale‐yellow oil. ${\left[\alpha \right]}_{D}^{23}$ = ‐ 38.9 (c = 0.75, MeOH). ^1^H NMR (400 MHz, CD_3_OD): *δ* = 3.90 (br s, 2H, H‐3), 3.83‐3.74 (m, 2H, H‐5), 3.44‐3.36 (m, 2H, H‐4), 2.88‐2.70 (m, 4H, Ha‐2, Ha‐6), 2.45‐2.32 (m, 4H, H‐7), 2.32‐2.21 (m, 2H, Hb‐2), 2.08 (br s, 2H, Hb‐6), 1.59‐1.45 (m, 4H, H‐8), 1.39‐1.30 (m, 4H, H‐9). ^13^C NMR (100 MHz, CD_3_OD): *δ* = 75.3 (d, 2C, C‐4), 69.5 (d, 2C, C‐5), 69.1 (d, 2C, C‐3), 59.2 (t, 2C, C‐7), 58.3 (t, 2C, C‐6), 57.6 (t, 2C, C‐2), 28.5 (t, 2C, C‐9), 27.5 (t, 2C, C‐8). MS (ESI) m/z (%) = 349.33 (9) [M+ H]^+^, 371.23 (100) [M+ Na]^+^, 387.17 (8), [M+ K]^+^. C_16_H_32_N_2_O_6_ (348.44): calcd C, 55.15; H, 9.26; N, 8.04; found C, 54.99; H, 9.44; N, 7.88.

### Synthesis of compound **1** **5**

Following the general procedure 12 M HCl (292 µL) was added to a solution of **12** (95 mg, 0.196 mmol) and furnished, after treatment with Ambersep 900‐OH, 79 mg of **15** (0.185 mmol, 95%) as a white solid (*Rf* = 0.00 CH_2_Cl_2_:MeOH:NH_4_OH (6%) 10:1:0.1).

**15**: white solid. M.p. 127.9‐129.5 °C. ${\left[\alpha \right]}_{D}^{23}$ = ‐ 35.6 (c = 0.7, MeOH). ^1^H NMR (400 MHz, CD_3_OD): *δ* = 3.91‐3.88 (m, 2H, H‐3), 3.82‐3.77 (m, 2H, H‐5), 3.39 (br s, 2H, H‐4), 2.78 (br s, 4H, Ha‐2, Ha‐6), 2.41‐2.24 (m, 4H, H‐7), 2.27‐2.24 (m, 2H, Hb‐2), 2.07 (br s, 2H, Hb‐6), 1.52‐1.49 (m, 4H, H‐8), 1.32 (br s, 12H, from H‐9 to H‐11). 13C NMR (100 MHz, CD_3_OD): *δ* = 111.4 (interference) 75.3 (d, 2C, C‐4), 69.5 (d, 2C, C‐5), 69.1 (d, 2C, C‐3), 59.4 (t, 2C, C‐7), 58.3 (t, 2C, C‐6), 57.6 (t, 2C, C‐2), 30.7 (t, 4C, C‐10, C‐11), 28.7 (t, 2C, C‐9), 27.5 (t, 2C, C‐8). MS (ESI) m/z (%) = 405.33 (100) [M+H]^+^, 203.17 (88) [M+H]^2+^, 427.33 (50) [M+ Na]^+^. C_20_H_40_N_2_O_6_ (404.55): calcd C, 59.38; H, 9.97; N, 6.92; found C, 59.32; H, 9.56; N, 6.62.

### Synthesis of compound **19**

Following the general procedure 12 M HCl (15 µL) was added to a solution of **18** (17 mg, 0.0581 mmol) and furnished, after treatment with Ambersep 900‐OH and purification by column chromatography (gradient eluent from CH_2_Cl_2_:MeOH:NH_4_OH (6%) 3:1:0.1 to 2:1:0.1), 8.5 mg of **19** (0.0337 mmol, 57%) as a colorless oil (*Rf* = 0.1 CH_2_Cl_2_:MeOH:NH_4_OH (6%) 3:1:0.1).

**19**: colorless oil. ${\left[\alpha \right]}_{D}^{24}$ = ‐ 6.0 (c = 0.8, MeOH). ^1^H NMR (400 MHz, CD_3_OD): *δ* = 7.37 (br s, 1H, Ar), 7.32‐7.21 (m, 3H, Ar), 3.91‐3.85 (m, 1H, H‐3), 3.82‐3.75 (m, 3H, H‐5, CH_2_NH_2_), 3.61‐3.50 (m, 2H, H‐7), 3.43‐3.33 (m, 1H, H‐4), 2.88‐2.63 (m, 2H, Ha‐2, Ha‐6), 2.29 (br d, J = 10.6 Hz, 1H, Hb‐2), 2.17‐1.97 (m, 1H, Hb‐6). ^13^C NMR (50 MHz, CD_3_OD): *δ* = 143.1 (s, 1C, Ar), 139.2 (s, 1C, Ar), 129.5 (d, 2C, Ar), 129.1 (d, 1C, Ar), 127.4 (d, 1C, Ar), 75.4 (d, 1C, C‐4), 69.7 (d, 1C, C‐5), 69.3 (d, 1C, C‐3), 63.2 (t, 1C, C‐7), 58.0 (t, 1C, C‐6), 57.4 (t, 1C, C‐2), 46.5 (t, 1C, CH_2_NH_2_). MS (ESI) m/z (%) = 253.10 (100) [M+H]^+^. C_13_H_20_N_2_O_3_ (252.31): calcd C, 61.88; H, 7.99; N, 11.10; found C, 61.50; H, 8.17; N, 10.98.

### Synthesis of compound **26**

Following the general procedure 12 M HCl (15 µL) was added to a solution of **25** (39 mg, 0.0501 mmol) and furnished, after treatment with Ambersep 900‐OH and purification by column chromatography (gradient eluent from CH_2_Cl_2_:MeOH:NH_4_OH (6%) 5:1:0.1 to 2:1:0.1), 27 mg of **26** (0.0386 mmol, 77%) as a white solid (*Rf* = 0.11 CH_2_Cl_2_:MeOH:NH_4_OH (6%) 3:1:0.1).

**26**: white solid. M. p. 145.4‐147.2 °C. ${\left[\alpha \right]}_{D}^{26}$ = ‐ 48.2 (c = 0.5, MeOH). ^1^H NMR (400 MHz, CD_3_OD): *δ* = 8.39 (s, 2H, triazole), 8.29 (s, 1H, Ar), 7.82 (d, J = 7.8 Hz, 2H, Ar), 7.51 (t, J = 7.8 Hz, 1H, Ar), 4.45 (t, J = 7.0 Hz, 4H, H‐14), 3.91‐3.86 (m, 2H, H‐3), 3.79 (td, J = 3.9, 7.8 Hz, 2H, H‐5), 3.45‐3.34 (m, 2H, H‐4), 2.90‐2.65 (m, 4H, Ha‐2, Ha‐6), 2.40‐2.30 (m, 4H, H‐7), 2.29‐2.20 (m, 2H, Hb‐2), 2.17‐2.02 (m, 2H, Hb‐6), 2.01‐1.88 (m, 4H, H‐13), 1.48 (quint, J = 7.3 Hz, 4H, H‐8), 1.40‐1.24 (m, 16H, H‐9, H‐10, H‐11, H‐12). ^13^C NMR (50 MHz, CD_3_OD): *δ* = 148.4 (s, 2C, triazole), 132.6 (s, 2C, Ar), 130.7 (d, 1C, Ar), 126.4 (d, 2C, Ar), 123.8 (d, 1C, Ar), 122.5 (d, 2C, triazole), 75.2 (d, 2C, C‐4), 69.5 (d, 2C, C‐5), 69.0 (d, 2C, C‐3), 59.2 (t, 2C, C‐7), 58.1 (t, 2C, C‐6), 57.5 (t, 2C, C‐2), 51.5 (t, 2C, C‐14), 31.2, 30.3, 29.9, 28.4, 27.4 (t, 12C, C‐8, C‐9, C‐10, C‐11, C‐12, C‐13). IR (neat): *ṽ* = 3429, 2923, 2850, 1463, 1430, 1344, 1307, 1226, 1087, 1072, 1063, 1021, 838 cm^−1^. MS (ESI) m/z (%) = 350.25 (66) [M+2H]^2+^, 699.42 (57) [M+H^+^], 721.58 (100) [M+Na]^+^. C_36_H_58_N_8_O_6_ (698.90): calcd C, 61.87; H, 8.36; N, 16.03; found C, 61.76; H, 8.56; N, 15.64.

### Synthesis of compound **37**

Following the general procedure 12 M HCl (30 µL) was added to a solution of **36** (35 mg, 0.0310 mmol) and furnished, after treatment with Ambersep 900‐OH and purification by column chromatography (gradient eluent from CH_2_Cl_2_:MeOH:NH_4_OH (6%) 5:1:0.1 to 2:1:0.1), 29 mg of **37** (0.0287 mmol, 94%) as a colorless oil (*Rf* = 0.12 CH_2_Cl_2_:MeOH:NH_4_OH (6%) 3:1:0.1).

**37**: colorless oil. ${\left[\alpha \right]}_{D}^{26}$ = ‐ 9.40 (c = 0.5, MeOH). ^1^H NMR (400 MHz, CD_3_OD): *δ* = 8.46 (s, 3H, triazole), 8.27 (s, 3H, Ar), 4.47 (t, J = 7.0 Hz, 6H, H‐14), 3.91‐3.86 (m, 3H, H‐3), 3.79 (td, J = 3.9, 7.8 Hz, 3H, H‐5), 3.46‐3.36 (m, 3H, H‐4), 2.88‐2.66 (m, 6H, Ha‐2, Ha‐6), 2.43‐2.31 (m, 6H, H‐7), 2.30‐2.22 (m, 3H, Hb‐2), 2.17‐2.03 (m, 3H, Hb‐6), 2.02‐1.92 (m, 6H, H‐13), 1.48 (quint, J = 7.2 Hz, 6H, H‐8), 1.39‐1.24 (m, 24H, H‐9, H‐10, H‐11, H‐12). ^13^C NMR (100 MHz, CD_3_OD): *δ* = 148.0 (s, 3C, triazole), 133.4 (s, 3C, Ar), 123.3 (d, 3C, Ar), 122.8 (d, 3C, triazole), 111.4 (interference), 75.1 (d, 3C, C‐4), 69.5 (d, 3C, C‐5), 69.0 (d, 3C, C‐3), 59.2 (t, 3C, C‐7), 58.1 (t, 3C, C‐6), 57.4 (t, 3C, C‐2), 51.6 (t, 3C, C‐14), 31.2, 30.4, 30.0, 28.4, 27.4 (t, 18C, C‐8, C‐9, C‐10, C‐11, C‐12, C‐13). IR (neat): *ṽ* = 3337, 3126, 2927, 2853, 2815, 1466, 1340, 1227, 1056, 837 cm^−1^. MS (ESI) m/z (%) = 337.25 (100) [M+3H]^3+^, 505.42 (52) [M+2H]^2+^, 1031.75 (23) [M+Na]^+^. C_51_H_84_N_12_O_9_ (1009.29): calcd C, 60.69; H, 8.39; N, 16.65; found C, 60.32; H, 8.64; N, 16.86.

### Synthesis of compound **45**

Following the general procedure 12 M HCl (45 µL) was added to a solution of **43** (34 mg, 0.0889 mmol) and furnished, after treatment with Ambersep 900‐OH and purification by column chromatography (CH_2_Cl_2_:MeOH:NH_4_OH (6%) 5:1:0.1), 26 mg of **45** (0.0759 mmol, 87%) as a white solid (*Rf* = 0.25, CH_2_Cl_2_:MeOH:NH_4_OH (6%) 3:1:0.1).

**45**: white solid. M.p. = 111.4‐112.1 °C. ${\left[\alpha \right]}_{D}^{24}$ = ‐ 21.3 (c = 1, MeOH). ^1^H NMR (400 MHz, CD_3_OD): *δ* = 7.90 (s, 1H, triazole), 4.67 (s, 2H, H‐15), 4.39 (t, J = 7.0 Hz, 2H, H‐14), 3.93‐3.86 (m, 1H, H‐3), 3.79 (td, J = 4.0, 7.8 Hz, 1H, H‐5), 3.43‐3.35 (m, 1H, H‐4), 2.86‐2.67 (m, 2H, Ha‐2, Ha‐6), 2.41‐2.32 (m, 2H, H‐7), 2.31‐2.23 (m, 1H, Hb‐2), 2.14‐2.00 (m, 1H, Hb‐6), 1.95‐1.83 (m, 2H, H‐13), 1.56‐1.43 (m, 2H, H‐8), 1.40‐1.22 (m, 8H, H‐9, H‐10, H‐11, H‐12) ppm. ^13^C NMR (100 MHz, CD_3_OD): *δ* = 149.0 (s, 1C, triazole), 124.0 (d, 1C, triazole), 75.2 (d, 1C, C‐4), 69.5 (d, 1C, C‐5), 69.1 (d, 1C, C‐3), 59.2 (t, 1C, C‐7), 58.2 (t, 1C, C‐6), 57.6 (t, 1C, C‐2), 56.5 (t, 1C, C‐15), 51.3 (t, 1C, C‐14), 31.3, 30.4, 30.0, 28.4, 27.5, 27.4 (t, 6C, C‐8, C‐9, C‐10, C‐11, C‐12, C‐13). IR (neat): *ṽ* = 3256, 3110, 2918, 2887, 2855, 2817, 1473, 1231, 1096, 1071, 1066, 1060, 1008, 1002 cm^−1^. MS (ESI) m/z (%) = 343.18 (100) [M+H]^+^, 365.20 (74) [M+Na]^+^, 706.82 (33) [2M+Na]^+^. Anal. Calcd for C_16_H_30_N_4_O_4_: C, 56.12; H, 8.83; N, 16.36. Found: C, 56.23; H, 8.71; N, 16.50.

### Synthesis of compound **31·2HCl**

A solution of **30** (17.7 mg, 0.0227 mmol) in MeOH (0.6 mL) was left stirring with 12 M HCl (8 µL) at room temperature for 15 h. The reaction mixture was concentrated to yield the corresponding hydrochloride salt **31·2HCl** (17.5 mg, 0.0227 mmol) as a mixture of diastereoisomers.

**31·2HCl**: yellow waxy solid. ^1^H NMR (400 MHz, CD_3_OD): *δ* = 8.75 (s, 2H, triazole), 7.91 (s, 4H, Ar), 4.50 ‐ 4.47 (m, 4H, H‐14), 4.08‐4.01 (m, 2H, H‐3), 3.91 (br s, 2H, H‐5), 3.79 – 3.78 (m, 2H, H‐4), 3.56 – 3.25 (m, 4H, H‐2 or H‐6), 3.19 – 3.01 (m, 8H, H‐7, H‐2 or H‐6), 1.94 (br s, 4H, H‐13), 1.65 – 1.60 (m, 4H, H‐8), 1.31 – 1.17 (m, 16 H, H‐9, H‐10, H‐11, H‐12); ppm. ^13^C NMR (100 MHz, CD_3_OD): *δ* = 111.4 (interference), 145.9, 129.7, 128.1, 124.9, 74.3, 69.3, 68.2, 67.5, 66.3, 64.2, 58.3, 57.9, 56.9, 56.8, 53.1, 52.8, 30.8, 29.9, 29.7, 27.4, 27.2, 25.1, 24.9 ppm. MS (ESI) m/z (%) = 350.25 (100) [M+2H]^2+^, 699.42 (30) [M+H]^+^. C_36_H_60_C_l2_N_8_O_6_ (771.83): calcd C, 56.02; H,7.84; N, 14.52; found C, 56.14; H, 8.28; N, 14.39.

### Synthesis of compound **32**

A solution of **31·2HCl** (64 mg, 0.0834 mmol) in dry pyridine (1.5 mL) was stirred with acetic anhydride (0.5 mL) at room temperature for 18 h until a TLC control attested the disappearance of **31·2HCl** (CH_2_Cl_2_:MeOH: NH_4_OH (6%) 10:1:0.1). The crude mixture was diluted with toluene and then concentrated under vacuum. Then, the crude residue was purified by FCC on silica gel (gradient eluent from CH_2_Cl_2_:MeOH:NH_4_OH (6%) 50:1:0.1 to 30:1:0.1) to give 37 mg of the acetylated compound **32** (0.0389 mmol, 47%) as a colorless oil (*Rf =* 0.27, CH_2_Cl_2_:MeOH: NH_4_OH (6%) 30:1:0.1).

**32**: colorless oil. ${\left[\alpha \right]}_{D}^{25}$ = + 15.2 (c = 1, CHCl_3_). ^1^H NMR (400 MHz, CDCl_3_): *δ* = 7.89 (s, 4H, Ar), 7.79 (s, 2H, triazole), 5.30‐5.24 (m, 2H, H‐3), 5.19‐5.11 (m, 2H, H‐5), 4.96‐4.89 (m, 2H, H‐4), 4.39 (t, J = 7.2 Hz, 4H, H‐14), 3.03‐2.92 (m, 2H, Ha‐6), 2.90‐2.78 (m, 2H, Ha‐2), 2.44‐2.28 (m, 6H, H‐7, Hb‐2), 2.26‐2.14 (m, 2H, Hb‐6), 2.08 (s, 6H, OAc), 2.03 (s, 6H, OAc), 2.02 (s, 6H, OAc), 1.98‐1.89 (m, 4H, H‐13), 1.46‐1.19 (m, 20H, H‐8, H‐9, H‐10, H‐11, H‐12). ^13^C NMR (100 MHz, CDCl_3_): *δ* = 170.6 (s, 2C, C = O), 170.2 (s, 4C, C = O), 147.4 (s, 2C, triazole), 130.5 (s, 2C, Ar), 126.2 (d, 4C, Ar), 119.6 (d, 2C, triazole), 71.3 (d, 2C, C‐4), 68.4 (d, 2C, C‐5), 68.1 (d, 2C, C‐3), 57.5 (t, 2C, C‐7), 54.5 (t, 2C, C‐2), 53.6 (t, 2C, C‐6), 50.6 (t, 2C, C‐14), 30.4 (t, 2C, C‐13), 29.3, 29.0, 27.2, 26.7, 26.5 (t, 10C, C‐8, C‐9, C‐10, C‐11, C‐12), 21.2 (q, 2C, OAc), 21.1 (q, 2C, OAc), 20.9 (q, 2C, OAc). IR (CHCl_3_): *ṽ* = 3688, 3024, 3017, 2934, 1742, 1371, 1260, 1234, 1090, 1049, 1016, 930 cm^−1^. MS (ESI) m/z (%) = 350.46 (100) [M+Na+2K]^3+^, 973.30 (42) [M+Na]^+^. C_48_H_70_N_8_O_12_ (951.12): calcd C, 60.61; H, 7.42; N, 11.78; found C, 60.70; H, 7.31; N, 11.50.

### Synthesis of compound **46·HCl**

A solution of **44** (24 mg, 0.0560 mmol) in MeOH (2 mL) was left stirring with 12 M HCl (30 µL) at room temperature for 18 h. The reaction mixture was concentrated to yield **46** as hydrochloride salt. The corresponding free amine was obtained by dissolving the residue in MeOH, then the strongly basic resin Ambersep 900 OH was added and the mixture was stirred for 2 h. The resin was removed by filtration and the mixture was concentrated under vacuum. The crude was purified by FCC on silica gel (CH_2_Cl_2_:MeOH:NH_4_OH (6%) 5:1:0.1) to give 22 mg of **46** (0.0560 mmol, quantitative yield) as a waxy white solid (*Rf* = 0.40, CH_2_Cl_2_:MeOH:NH_4_OH (6%) 5:1:0.1), insoluble in all the tested deuterated solvent and impossible to be characterized. Therefore, a solution of **46** (22 mg, 0.0560 mmol) in MeOH (2 mL) was left stirring with 12 M HCl (60 µL) at room temperature for 4 h to yield 24 mg of the hydrochloride salt **46·HCl** (0.0560 mmol) as a colorless oil, and as a 2:1 mixture of diastereoisomers (isomer A major:isomer B minor).

**46·HCl**: colorless oil. ^1^H NMR (400 MHz, CD_3_OD): *δ* = 9.01 (s, 2H, triazole, isomer A and B), 7.93‐7.81 (m, 4H, Ar, isomer A and B), 7.65‐7.49 (m, 6H, Ar, isomer A and B), 4.72‐4.61 (m, 4H, H‐14, isomer A and B), 4.22‐4.15 (m, 1H, H‐3, isomer A), 4.15‐4.10 (m, 1H, H‐3, isomer B), 4.08‐3.97 (m, 2H, H‐5, isomer A and B), 3.95‐3.85 (m, 1H, H‐4, isomer A), 3.57‐3.48 (m, 2H, H‐4, Ha‐2, isomer B), 3.47‐3.38 (m, 1H, Ha‐6, isomer B), 3.31‐3.27 (m, 1H, Ha‐6, isomer A), 3.24‐3.10 (m, 7H, Ha‐2, Hb‐6, isomer A and Hb‐2, isomer B and H‐7, isomer A and B), 3.03 (t, J = 11.3 Hz, 1H, Hb‐2, isomer A), 2.84 (t, J = 11.2 Hz, 1H, Hb‐6, isomer B), 2.16‐2.01 (m, 4H, H‐13, isomer A and B), 1.86‐1.65 (m, 4H, H‐8, isomer A and B), 1.50‐1.33 (m, 16H, H‐8, H‐9, H‐10, H‐11, isomer A and B). ^13^C NMR (100 MHz, CD_3_OD): *δ* = 145.4, 131.9, 130.7, 127.7, 126.3, 125.8, 74.3, 69.3, 68.1, 67.5, 66.3, 64.2, 58.3, 57.9, 56.9, 56.8, 54.1, 53.1, 52.8, 30.5, 29.8, 29.7, 27.4, 27.1, 25.1, 24.9. IR (neat): *ṽ* = 3312, 3209, 3072, 3044, 2942, 2929, 2860, 2195, 1884, 1468, 1444, 1373, 1104, 1076, 1051, 961 cm^−1^. MS (ESI) m/z (%) = 389.23 (100) [M+H]^+^, 411.26 (22) [M+Na]^+^, 798.90 (42) [2M+Na]^+^. Anal. Calcd for C_21_H_33_ClN_4_O_3_: C, 59.35; H, 7.83; N, 13.18. Found: C, 58.95; H, 7.90; N, 12.90.

### Inhibitory activity toward human GCase from leukocyte homogenates

All experiments on biological materials were performed in accordance with the ethical standards of the institutional research committee and with the 1964 Helsinki Declaration and its later amendments. In keeping with ethical guidelines, all blood and cell samples were obtained for storage and analyzed only after written informed consent of the patients (and/or their family members) was obtained, using a form approved by the local Ethics Committee (Codice Protocollo: Lysolate ‘’Late onset Lysosomal Storage Disorders (LSDs) in the differential diagnosis of neurodegenerative diseases: development of new diagnostic procedures and focus on potential pharmacological chaperones (PCs). Project ID code:16774_bio, May 5, 2020, Comitato Etico Regionale per la Sperimentazione Clinica della Regione Toscana, Area Vasta Centro, Florence, Italy). Control and patient samples were anonymized and used only for research purposes.

Compounds **13**, **14**, **15**, **19**, **26**, **31**, **37**, **45**, and **46** were screened toward GCase from leukocytes isolated from healthy donors (controls). Isolated leukocytes were lysed by sonication, and the protein content of solutions was determined using a micro BCA protein assay kit (Sigma‐Aldrich), according to the manufacturer's instructions. The GCase activity of the leukocyte extracts was measured in a flat‐bottomed 96‐well plate in a final volume of 30 µL using 4‐methylumbelliferyl‐β‐d‐glucoside as substrate (Sigma‐Aldrich). Each assay solution contained 3 µL of iminosugar solution, 7 µL of leukocytes homogenate (protein content 4.29 µg/µL), and 20 µL of substrate (3.33 mM concentration) dissolved in citrate/phosphate buffer (0.1  : 0.2, M/M, pH 5.8) containing sodium taurocholate (0.3%) and Triton X‐100 (0.15%). All samples were incubated for 1 h at 37 °C. After this time, the reactions were stopped by adding 200 µL of a solution containing 0.5 M sodium carbonate and 0.025% Triton X‐100. The fluorescence of 4‐methylumbelliferone released by β‐glucosidase activity was measured in SpectraMax M2 microplate reader (λex = 365 nm, λem = 435 nm; Molecular Devices). Percentage GCase inhibition is given with respect to the control (without iminosugar). Data are mean + SD (n = 3). See the Supporting Information for further details.

The IC_50_ values of **26**, **31**, **37**, **45**, and **46** against GCase were determined by measuring the initial hydrolysis rate of 4‐methylumbelliferyl‐β‐d‐glucoside substrate (3.33 mM) in the presence of increasing inhibitor concentrations. See the Supporting Information for further details.

The action mechanism of compounds **26**, **31**, **37**, and **46** was determined studying the dependence of the main kinetic parameters (K_m_ and V_max_) from the inhibitor concentration. The GCase activity was determined using the 4‐methylumbelliferyl‐β‐d‐glucoside as substrate. Kinetic data obtained were analyzed using the *Lineweaver*‐*Burk* plot. See the Supporting Information for further details.

### NMR Spectroscopy

Nuclear magnetic resonance (NMR) spectra were acquired on a Bruker 600 MHz Avance Neo instrument equipped with a cryo‐probe. NMR samples were dissolved in deuterated PBS buffer; [D4] (trimethylsilyl)propionic acid, sodium salt (TSP, 10 µM) was used as internal reference to calibrate all of the spectra. Data acquisition and processing were analyzed by using TOPSPIN 4.4 software. Heteronuclear single quantum coherence (HSQC) experiments were carried out with setting data points of 2048 × 600. STD NMR experiments were perforemed by using a protein:ligand ratio of 1:80 and a saturation time of 2 s with the on‐resonance pulse at ‐1 ppm and the off‐resonance pulse at 40 ppm. By using these conditions, no STD signals were observed in the control STD NMR spectrum of the ligand alone. A train of 50 ms (field strength of 21 Hz) Gaussian‐shaped pulse with an attenuation of 60 db has been used to saturate the protein.

### Computational studies

Compounds **37** and **48** were parameterized following a tailored protocol combining quantum and classical approaches. Geometry optimizations and atomic charge derivation were performed using Gaussian 09 [70] at the HF/6‐31G* level, applying the RESP methodology for electrostatic potential fitting. The resulting parameters were integrated into the AMBER force field via Antechamber and xLeap [71], generating the required  .prep and  .frcmod files. Trajectories were analyzed using the ptraj module from the AMBER 18 suite [72], and molecular visualization of the MD simulations was carried out using VMD [73].

Docking studies were conducted in two stages. Initial cavity detection and blind docking were performed using CB‐Dock2 [74, 75], which automatically identifies potential binding pockets and generates preliminary docking poses using AutoDock Vina [76, 77]. This analysis provided a first approximation of plausible binding sites on the GCase surface. Subsequently, refined docking calculations were carried out using AutoDock Vina, with ligand and protein preparation conducted in UCSF ChimeraX 1.8 [78]. For both compounds **37** and **48**, docking was performed within a grid box of dimensions 35 × 30 × 40 Å^3^, using an exhaustiveness parameter set to 64 to ensure extensive conformational sampling. The generated binding modes were ranked by predicted binding free energy (affinity, in kcal/mol). Each mode is also associated with a pair of RMSD values (lower and upper bounds) relative to the best‐scoring pose, which reflect the structural deviation used for clustering. The docking poses were grouped based on structural similarity using an RMSD threshold of 2.0 Å, and representative conformations were selected from each cluster. For both ligands, the pose with the lowest predicted binding energy (mode 1) was chosen as the reference structure for subsequent analysis.

Molecular dynamics (MD) simulations were carried out using the AMBER 18 software package. The enzyme GCase was modelled from chain D of the crystallographic structure PDB ID: 2NSX. Protein preparation was performed using the Protein Preparation Wizard in Maestro (Schrödinger), including hydrogen addition, optimization of ionizable states, and capping of terminal residues. ff14SB force field [79] was used for the protein, while the solvent was modelled with TIP3P water molecules arranged in a truncated octahedral box extending 15 Å beyond the solute. System neutrality was achieved by the addition of counterions. A hybrid force field strategy was adopted for the parametrized ligands **37** and **48**: the iminosugar moieties were treated with GLYCAM06j‐1 [80], whereas the triazole linkers, alkyl spacers, and aromatic units were assigned GAFF2 parameters [81]. Ligand and system preparation was completed using the tleap module.

Initial energy minimization was conducted in two stages using the SANDER module: first with positional restraints on the solute, followed by full system minimization. The system was then gradually heated from 0 to 300 K over 100 ps under constant volume conditions, followed by a pressure equilibration phase at 1 atm. Production MD simulations were run for 20 ns in the NPT ensemble using the PMEMD module, with periodic boundary conditions and the particle mesh Ewald method (grid spacing 1 Å) applied to handle long‐range electrostatics. A time step of 2 fs was used, and coordinates were saved every 2 ps.The duration of 20 ns was chosen as a sufficient timescale to assess the local stability of the ligand poses obtained from docking, particularly the behavior of the iminosugar units within the catalytic pocket. Longer timescales were not required at this stage, as the primary objective was to confirm initial anchoring rather than explore global protein conformational dynamics. Trajectory analysis was performed using ptraj and CPPTRAJ [78, 82]. RMSD‐based clustering of the ligand conformations was carried out using the K‐means algorithm, and the most populated cluster was used to extract representative binding modes. Hydrogen bonds were defined by a donor–acceptor distance ≤ 3.0 Å and an A–H–D angle ≥ 135°. Dihedral behavior of key torsional angles was analyzed via custom scripts to generate histogram plots of preferred conformations. Visualization and figure preparation were performed with VMD and PyMOL [83].

## Conflicts of Interest

The authors declare no competing interest.

## Supporting information




**Supporting file**: chem70468‐sup‐0001‐SuppMat.pdf.

## Data Availability

The data underlying this study are available in the published article and online Supporting Information.
