# Supplementary material for: Simultaneous Grafting of 3,4,5‐Trihydroxypiperidine Iminosugars Onto Multivalent Scaffolds via Double Reductive Amination Provides New GCase Inhibitors
Source: Chemistry. 2025 Dec 8;32(2):e02436. doi: 10.1002/chem.202502436 (PMC12790318; doi:10.1002/chem.202502436)
Supplement: Supplementary file 1 — Supporting file: chem70468‐sup‐0001‐SuppMat.pdf. [file CHEM-32-e02436-s001.pdf]

t

Chemistry–A European Journal

Supporting Information

Simultaneous Grafting of 3,4,5-Trihydroxypiperidine Iminosugars onto Multivalent Scaffolds via Double Reductive Amination Provides New GCase Inhibitors

Maria Giulia Davighi,[a] Francesca Clemente,[a] Alessio Morano,[a] Francesca Mangiavacchi,[a] Francesca Cardona,[a]* Andrea Goti,[a] Paolo Paoli,[b] Amelia Morrone,[c, d] Ferran Nieto-Fabregat,[e] Roberta Marchetti[e] and Camilla Matassini[a]*

[a] Dr. M. G. Davighi, Dr. F. Clemente, A. Morano, Dr. F. Mangiavacchi, Prof. F. Cardona, Prof. A. Goti, Prof. C. Matassini, Department of Chemistry "Ugo Schiff" (DICUS), University of Florence, Via della Lastruccia 3-13, 50019 Sesto F.no (FI), Italy.

[b] Prof. P. Paoli, Department of Experimental and Clinical Biomedical Sciences University of Florence Viale Morgagni 50, 50134 Firenze, Italy.

[c] Prof. A. Morrone, Laboratory of Molecular Genetics of Neurometabolic Diseases, Neuroscience Department Meyer Children's Hospital, IRCCS, Viale Pieraccini 24, 50139 Firenze, Italy.

[d] Prof. A. Morrone, Department of Neurosciences, Psycology, Drug Research and Child Health (NEUROFARBA) University of Florence, Viale Pieraccini 24, 50139 Firenze, Italy.

[e] Prof. R. Marchetti, Dr. F. Nieto-Fabregat, Department of Chemical Sciences, University of Naples Federico II, Via Cinthia 4, Naples 80126, Italy.

Table of contents:

- ESI-MS analysis after acetylation of the crude mixture to attest the formation of **16** S3
- Proposed mechanisms for the reaction between the benzylated aldehyde **1** and hexamethylene diamine **8** S4
- Attempts to obtain compound **31** and ^{1}H-NMR spectra of crude mixtures obtained after deacetylation of **32** S6
- Synthesis of compound **10** (*Route I*) S7
- Synthesis of compound **43** (*Route II*) S7
- ^{1}H NMR and ^{13}C NMR spectra of new compounds S8
- Inhibitory activity towards human GCase from leukocyte homogenates S37
- IC_{50} determination and curves S37
- Kinetic analysis for compounds **26**, **31**, **37** and **46** S40
- Computational studies S44

ESI-MS analysis after acetylation of the crude mixture to attest the formation of 16

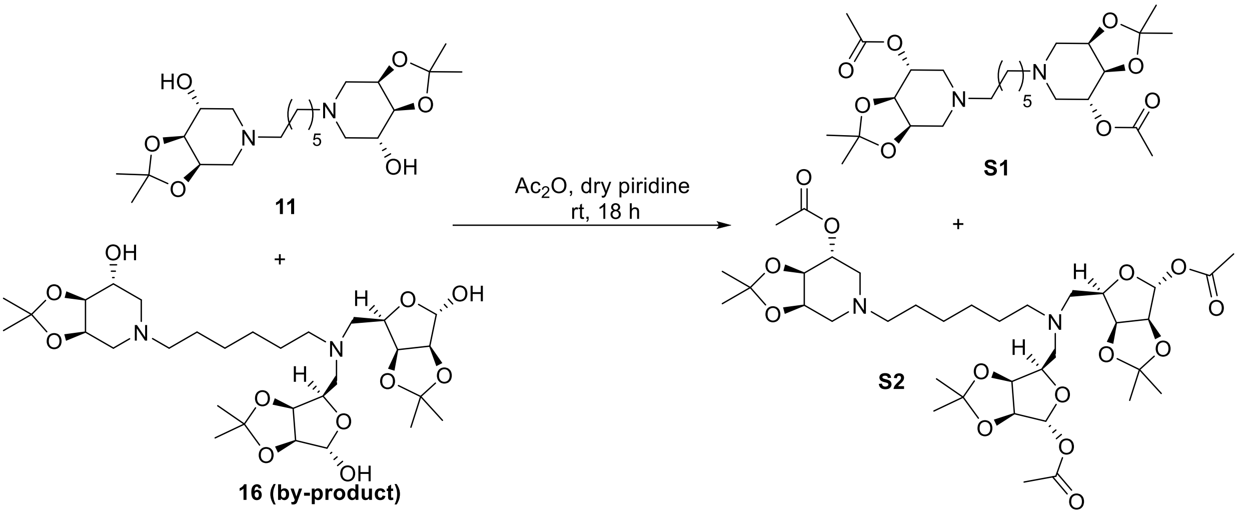

Compound 11 (MW= 428.57)
MS (ESI) m/z (%) = 429 (87) [M+H]+
Compound 16 (MW= 616.75)
MS (ESI) m/z (%) = 617.28 (100) [M+H]+, 639.28 (74) [M+Na]+

Scheme S1: Acetylation of the crude mixture containing divalent **11** and by-product **16**.

A solution of crude mixture (**16** + **11**) in dry pyridine (0.2 mL) and acetic anhydride (0.3 mL) was stirred at room temperature for 18 h. The crude mixture was diluted with toluene and then concentrated under vacuum. Then, the crude residue was analysed by MS (ESI) to attest the presence of acetylated compound **16**: compound **S2** (MW= 742.86) MS (ESI) m/z (%) = 765.17 (100) $[M+Na]^+$, 743.19 (100) $[M+H]^+$ and compound **S1** (MW= 512.64) MS (ESI) m/z (%) = 535.13 (23) $[M+Na]^+$.

Proposed mechanisms for the reaction between the benzylated aldehyde 1 and hexamethylene diamine 8

Considering the obtained experimental data, the mechanism reported in the scheme 1 was proposed for the reaction between the aldehyde **1** and hexamethylenediamine to obtain both the products **11** and **16**.

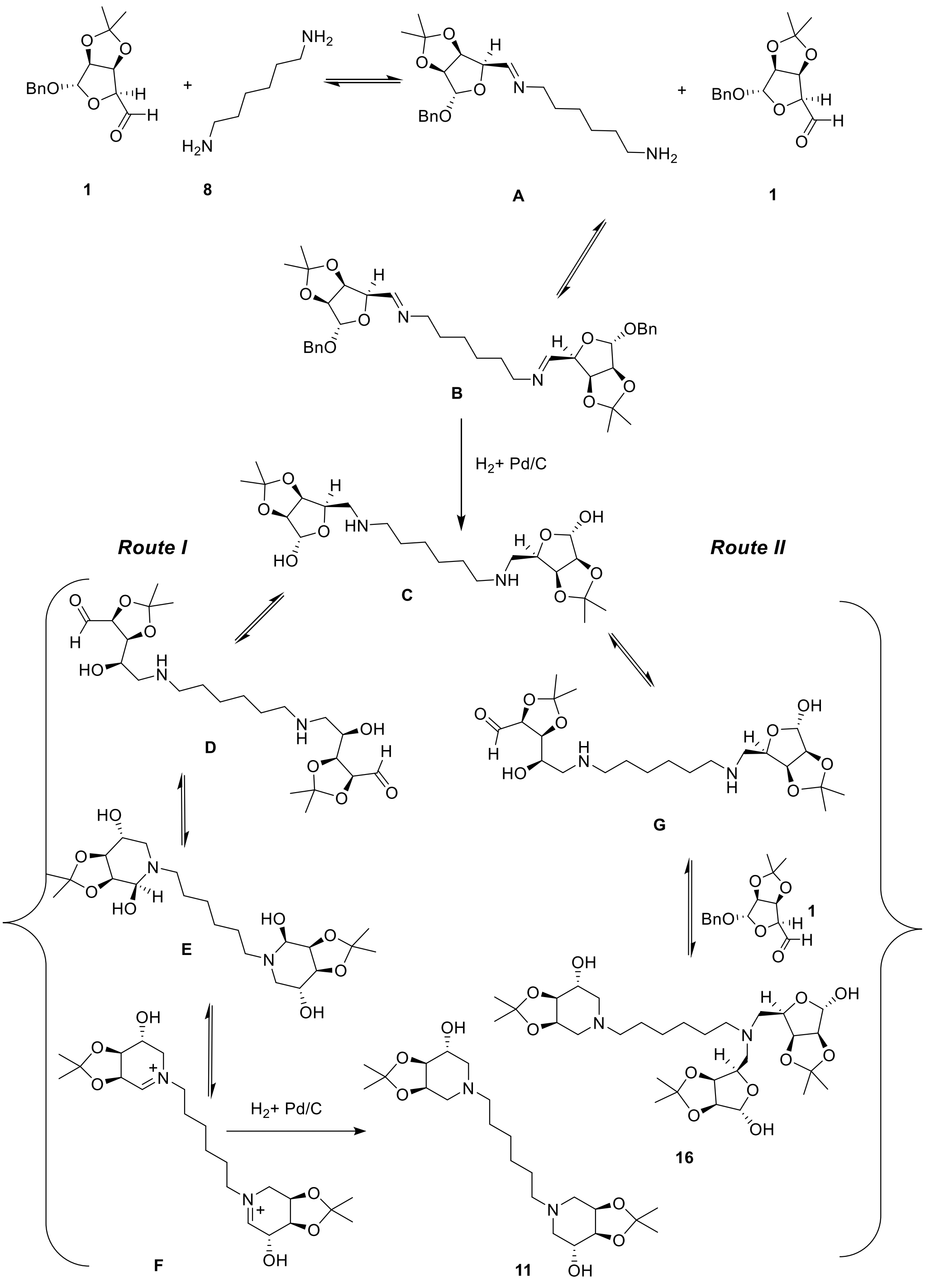

Scheme S2: mechanism proposed for the DRA between aldehyde **1** and hexamethylenediamine.

The first steps are equilibria that involved the condensation of the hexamethylenediamine with the exocyclic aldehyde group of **1** to give the imine intermediates **A** and **B**. The reduction of imine moieties with H_2 in the presence of $Pd(OH)_2/C$ furnished the diamine **C**. Then, the intermediate **C** can evolve following two different

routes. Another intramolecular reductive amination provided the divalent **11** after the loss of water and the reduction of the iminium ions (*Route* I, scheme 1). Alternatively, in the presence of an excess of **1**, the second amine in **G** can react with another molecule of **1** through a reductive amination reaction to give the by-product **16** bearing a tertiary amine (*Route* II, scheme 1).

Attempts to obtain compound 31 and ^{1}H-NMR spectra of crude mixtures obtained after deacetylation of 32

Treatment of **30** with HCl in MeOH at room temperature for 18 hours gave the hydrochloride salt **31·2HCl** which we attempted to purify via FCC with a polar basic eluent (CH_2Cl_2:MeOH: 6% NH_4OH 10:1:0.1) to concomitantly liberate the free amine. Unfortunately, neither the hydrochloride salt nor the free amine was recovered from the column, forcing us to turn to a protection/deprotection strategy. Hence, compound **31·2HCl** was prepared and fully acetylated by treatment with acetic anhydride in dry pyridine affording compound **32** with 47% yield after FCC (Scheme 6). However, subsequently attempts to remove the acetyl groups of **32**, performed both with Na_2CO_3 in MeOH and with ammonia solution (4M ammonia in methanol; MercK), failed in obtaining the pure **31** (see the Supporting Information).

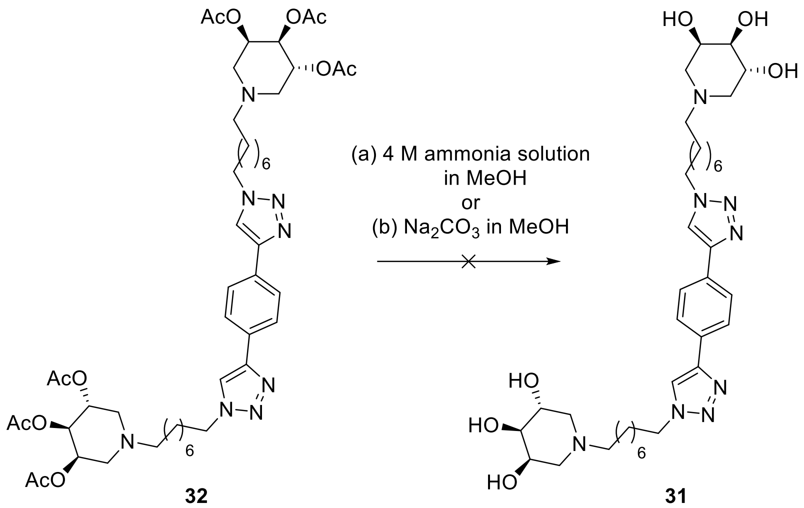

Scheme S3: Deacetylation of the **32** in different conditions.

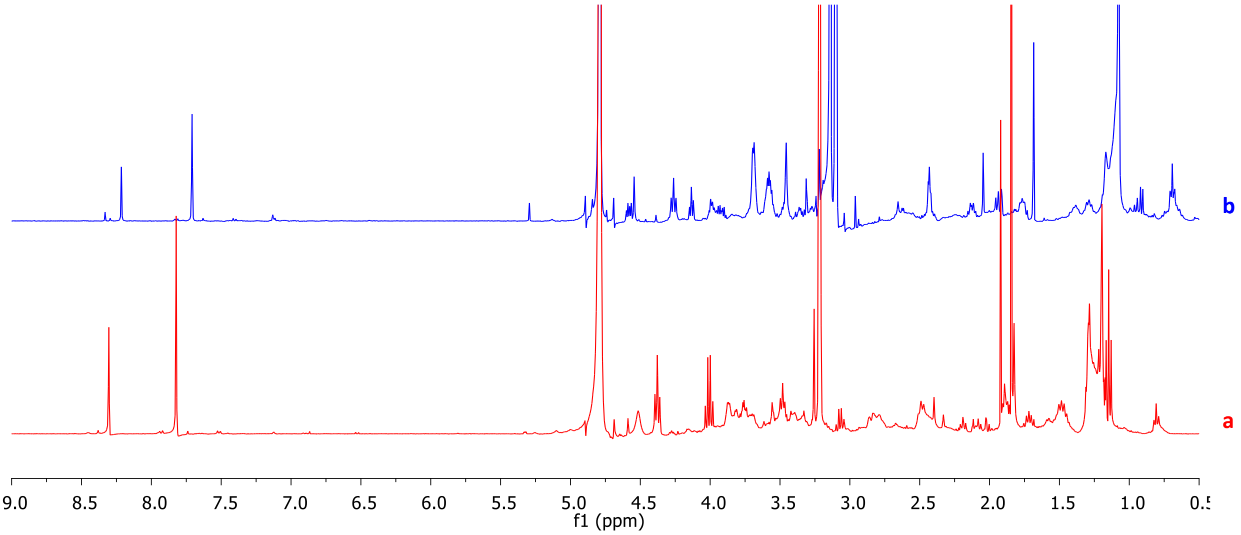

Figure S1: (a) spectrum of the crude mixture obtained after deprotection of **32** with a 4 M ammonia solution in MeOH; (b) spectrum of the crude mixture obtained after deprotection of **32** with Na_2CO_3 in MeOH.

Synthesis of compound 10 (*Route I*)

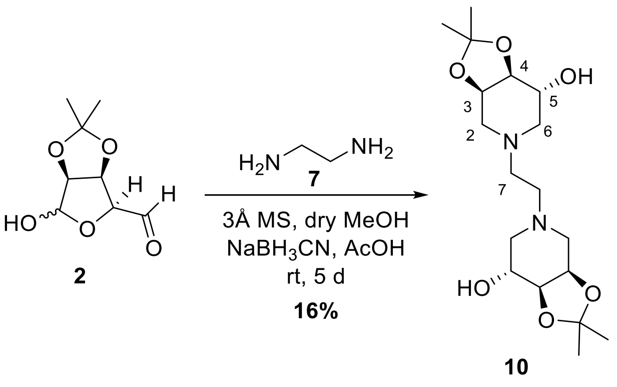

A solution of dialdehyde **2** (107 mg, 0.569 mmol) in dry MeOH (1 mL) was stirred in the presence of 3Å molecular sieves powder under nitrogen atmosphere for 15 min, and then $NaBH_3CN$ (107 mg, 1.703 mmol), AcOH (65 µL, 1.14 mmol) and ethylenediamine **7** (19 µL, 0.285 mmol) were added. The mixture was stirred for five days under nitrogen atmosphere until molecular sieves were removed by filtration through Celite and the filtrate was concentrated under vacuum. The residue was purified by flash chromatography (gradient eluent from CH_2Cl_2:MeOH:$NH_4(OH)$ (6%) 20:1:0.1 to 10:1:1) to afford 17 mg of **10** (0.0456 mmol, 16%) as a white waxy solid (*Rf* = 0.33, CH_2Cl_2:MeOH:NH_4OH (6%) 10:1:0.1).

Synthesis of compound 43 (*Route II*)

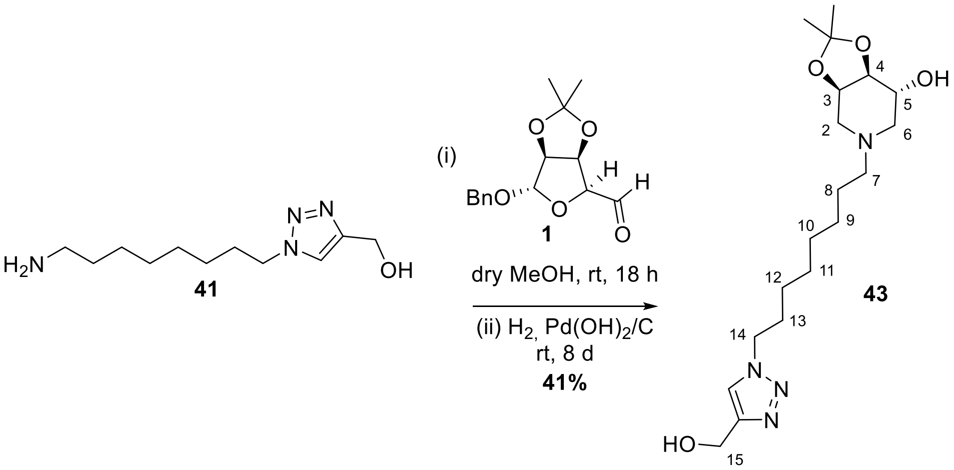

A solution of dialdehyde **1** (36 mg, 0.129 mmol) and amine **41** (29 mg, 0.128 mmol) in dry MeOH (2 mL) was stirred for 18 hours under nitrogen atmosphere. Then, $Pd(OH)_2/C$ (15 mg) was added and nitrogen was replaced by hydrogen gas, bubbling hydrogen with a balloon and the reaction mixture was stirred at room temperature for 8 days until ^1H NMR control assessed the disappearance of aromatic protons and the presence of **43**. The catalyst was removed by filtration on a short pad of Celite, washed several times with MeOH, and the solvent was evaporated under vacuum. The crude was purified by flash chromatography on silica gel (CH_2Cl_2:MeOH:NH_4OH (6%) 15:1:0.1) to afford 20 mg of **43** (0.0523 mmol, 41%) as a pale yellow-oil (*Rf* = 0.26, CH_2Cl_2:MeOH:NH_4OH (6%) 10:1:0.1).

^{1}H NMR and ^{13}C NMR spectra of new compounds

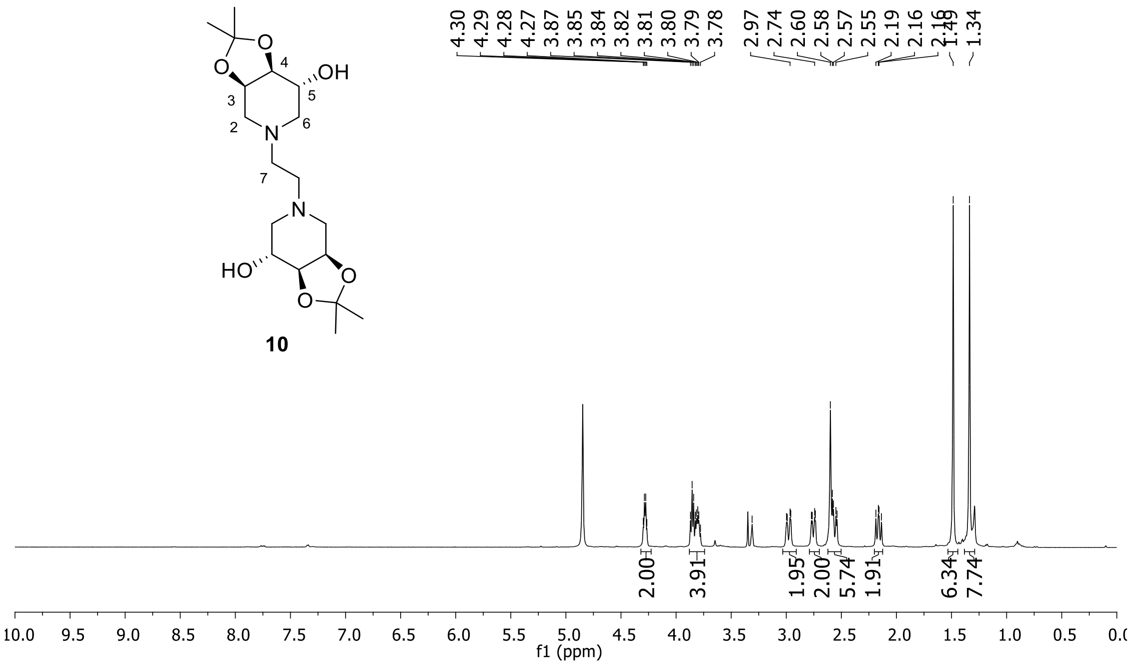

^{1}H-NMR spectrum of compound **10** (400 MHz, CD_3OD).

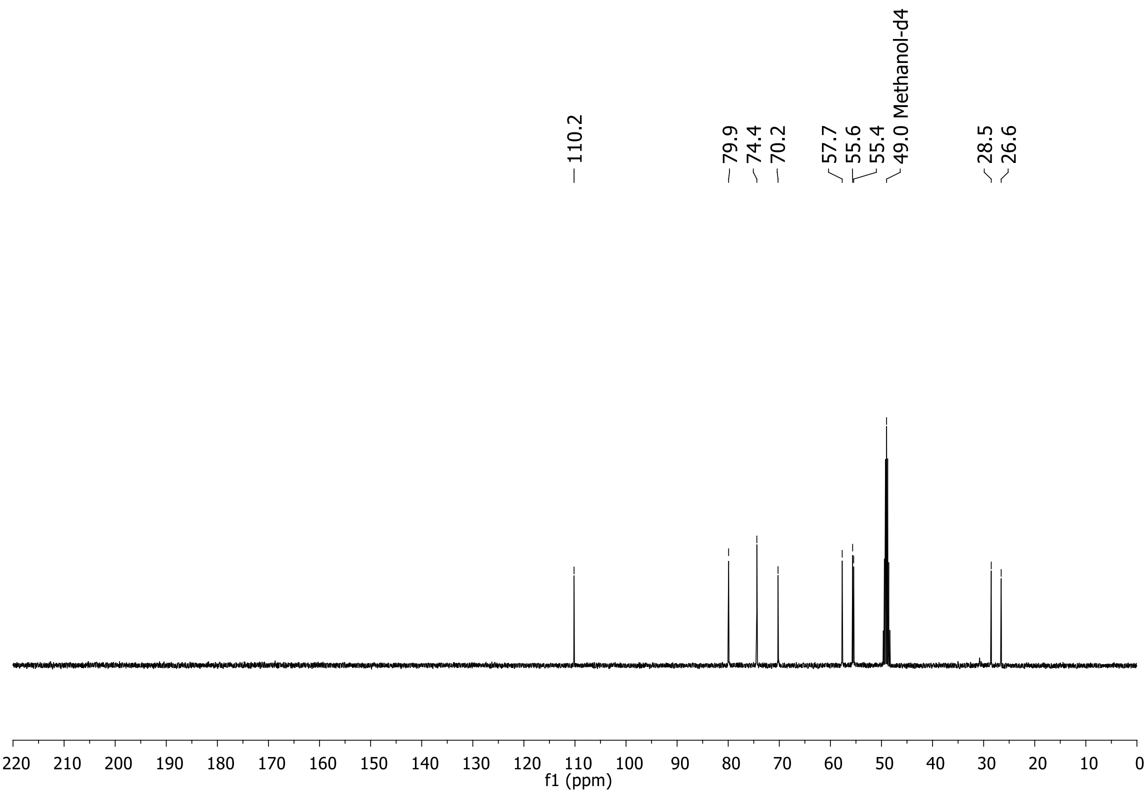

^{13}C-NMR spectrum of compound **10** (100 MHz, CD_3OD).

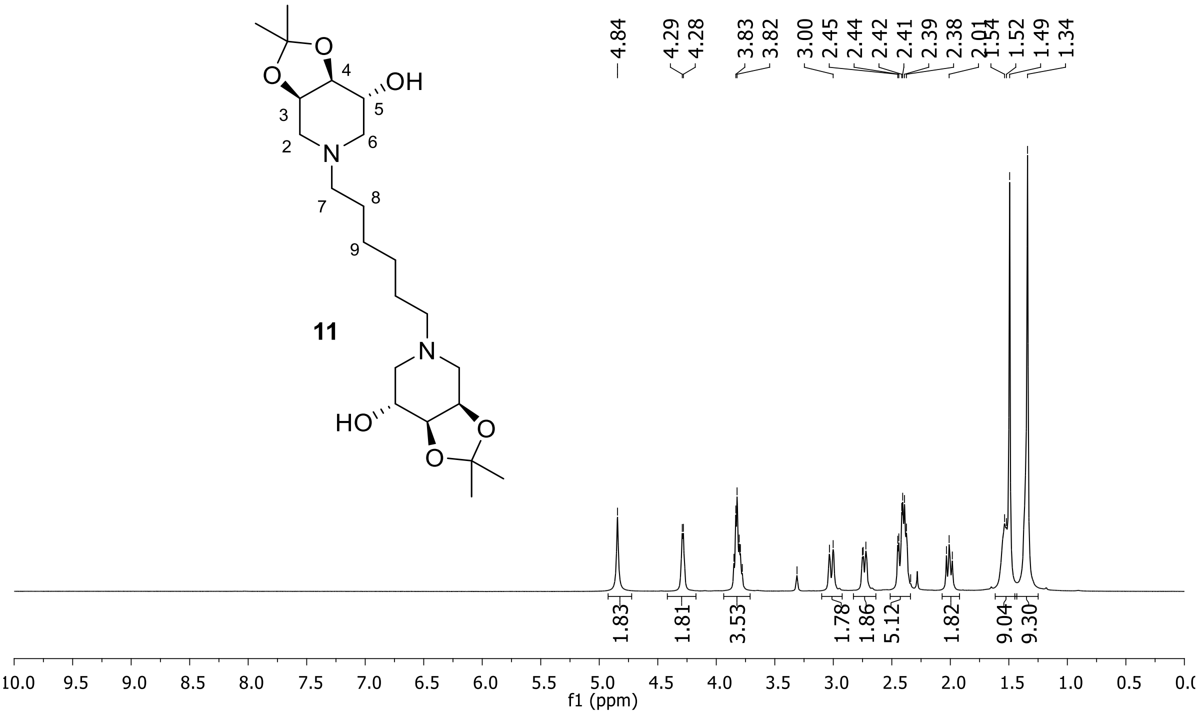

^{1}H-NMR spectrum of compound **11** (400 MHz, CD_3OD).

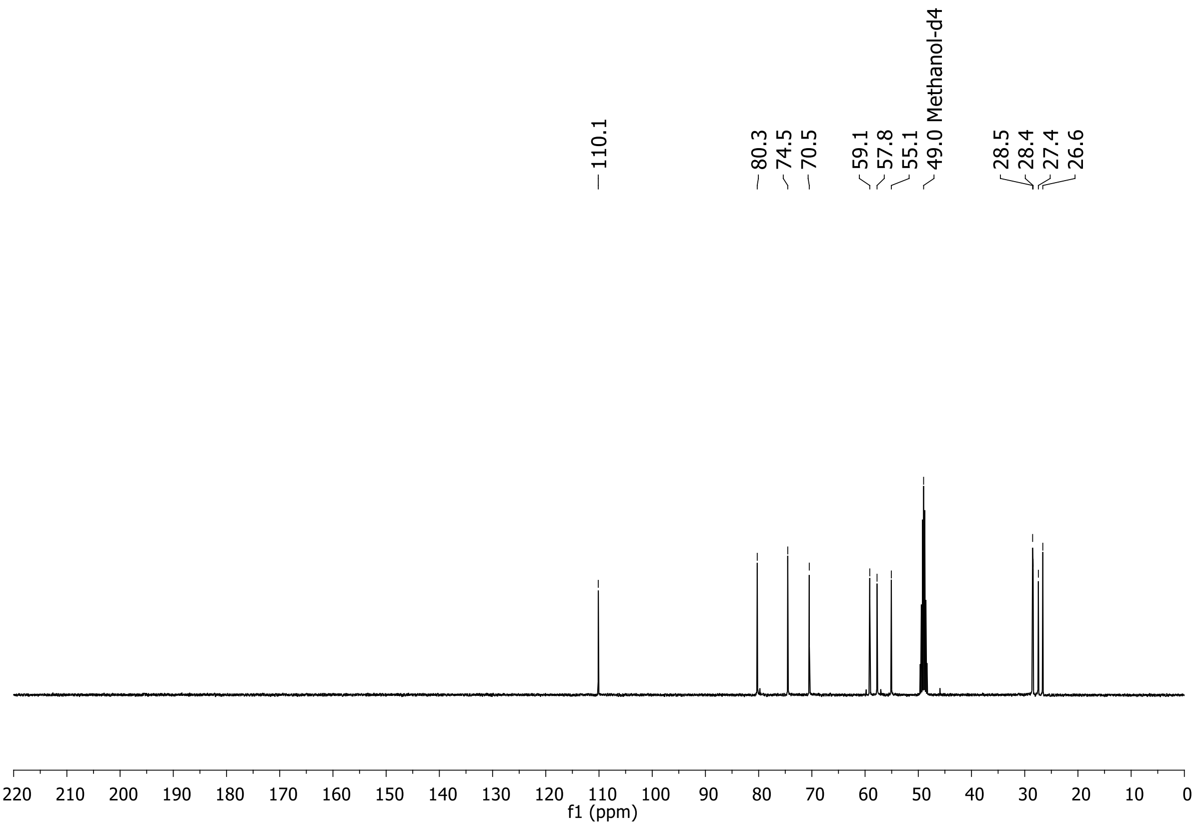

^{13}C-NMR spectrum of compound **11** (100 MHz, CD_3OD).

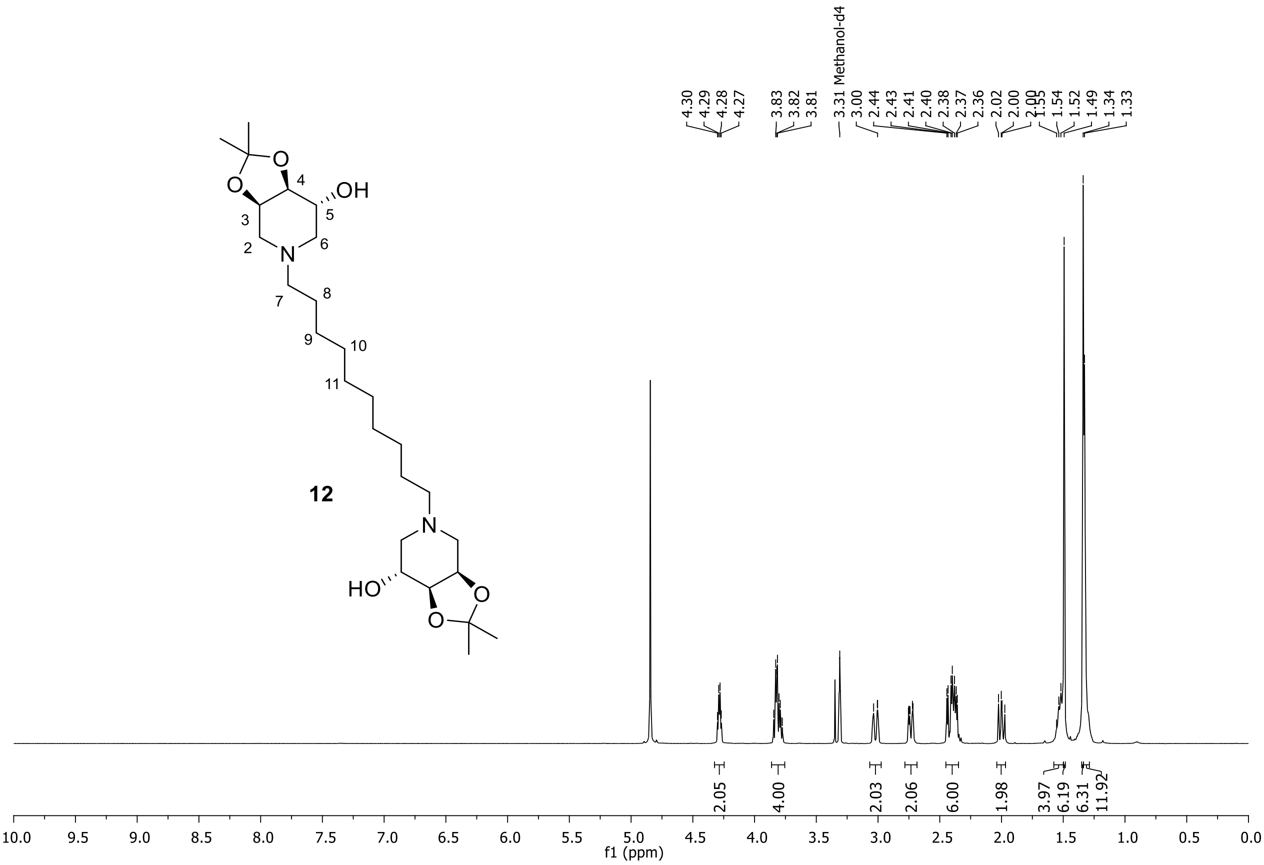

^{1}H NMR spectrum of compound **12** (400 MHz, CD_3OD).

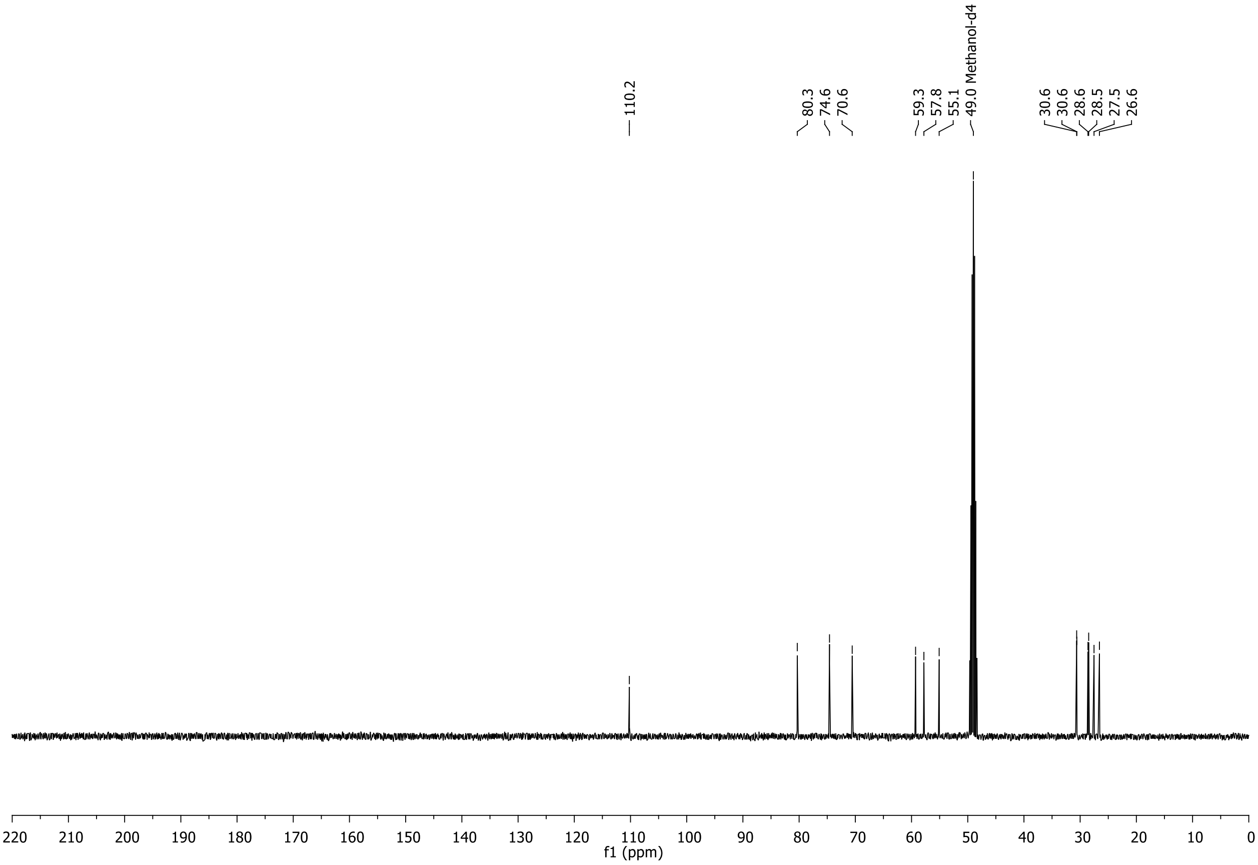

^{13}C NMR spectrum of compound **12** (100 MHz, CD_3OD).

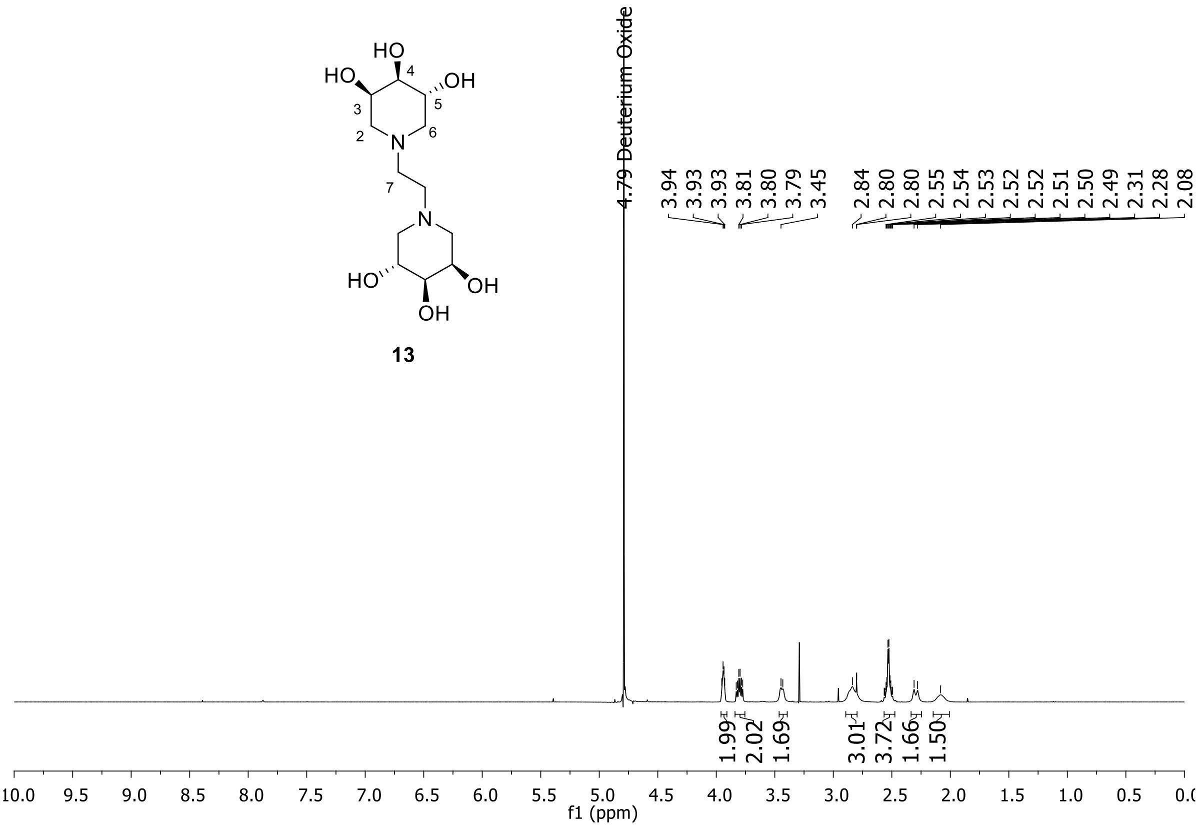

^{1}H-NMR spectrum of compound **13** (400 MHz, D_2O).

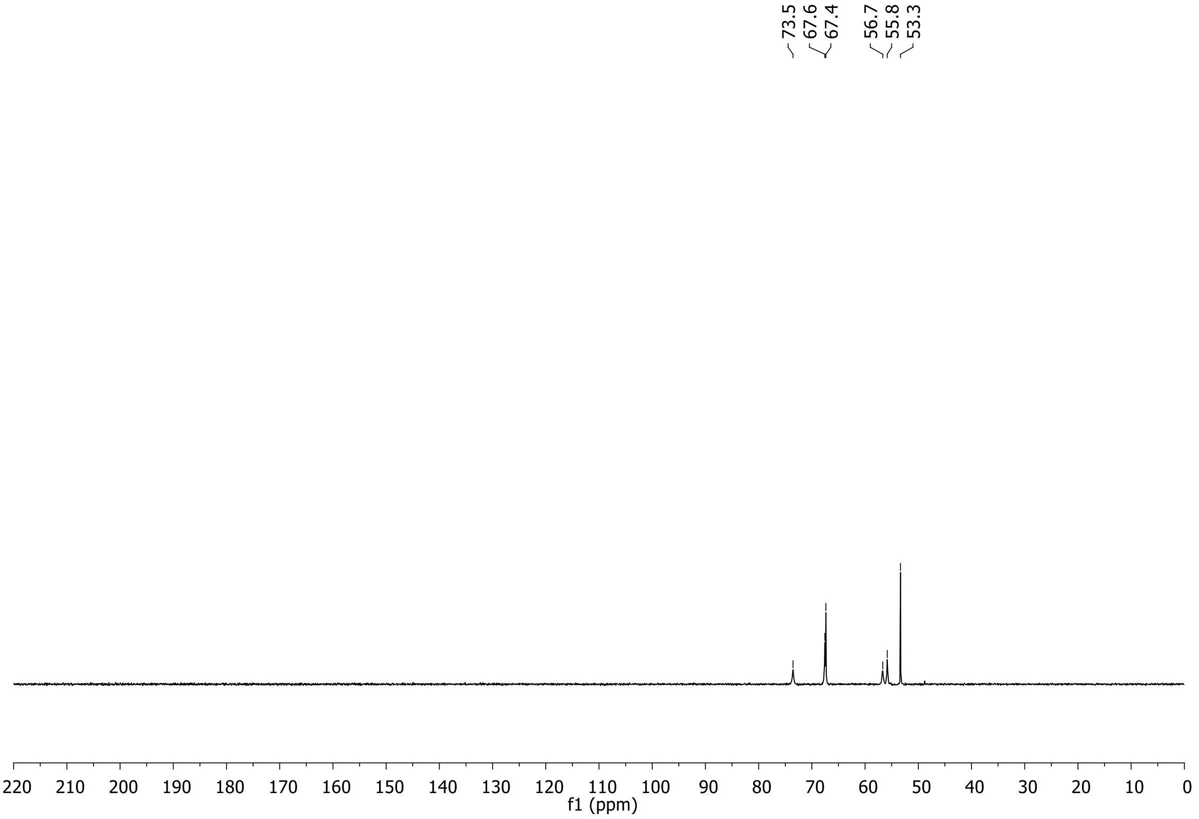

^{13}C-NMR spectrum of compound **13** (100 MHz, D_2O).

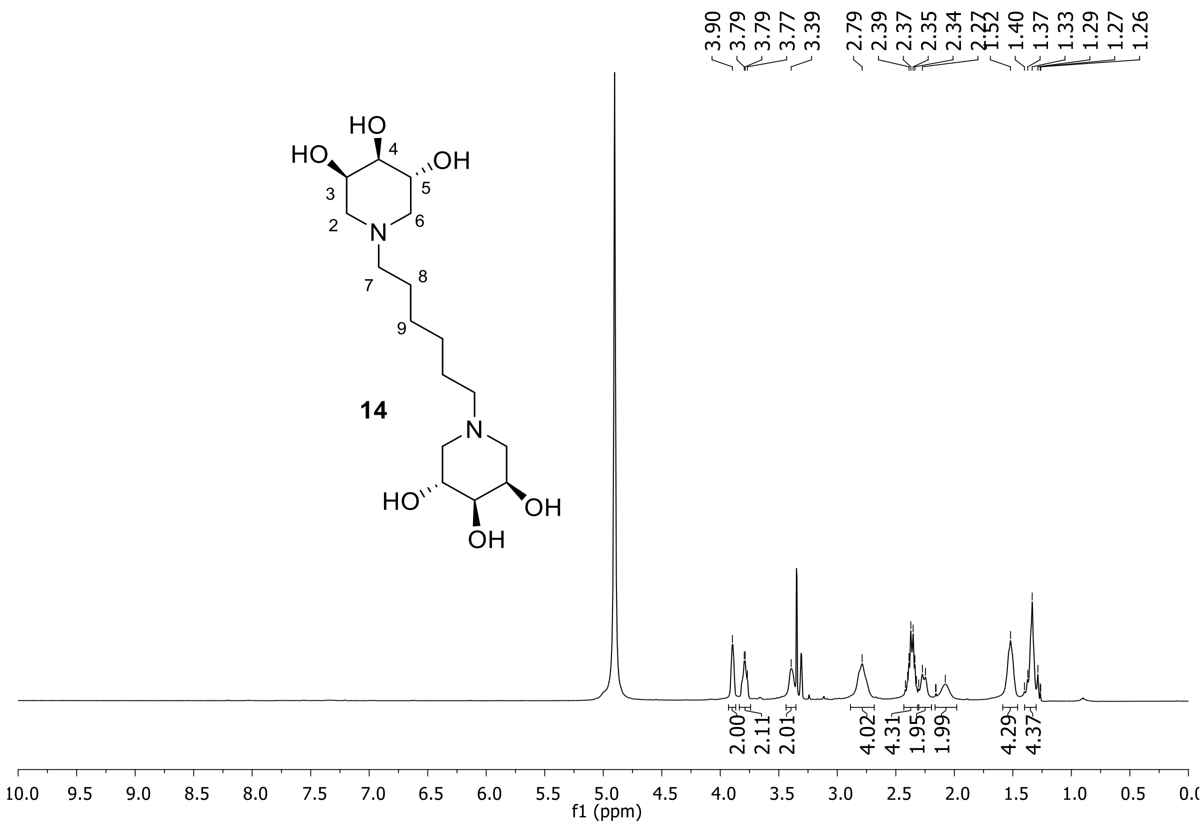

^{1}H-NMR spectrum of compound **14** (400 MHz, CD_3OD).

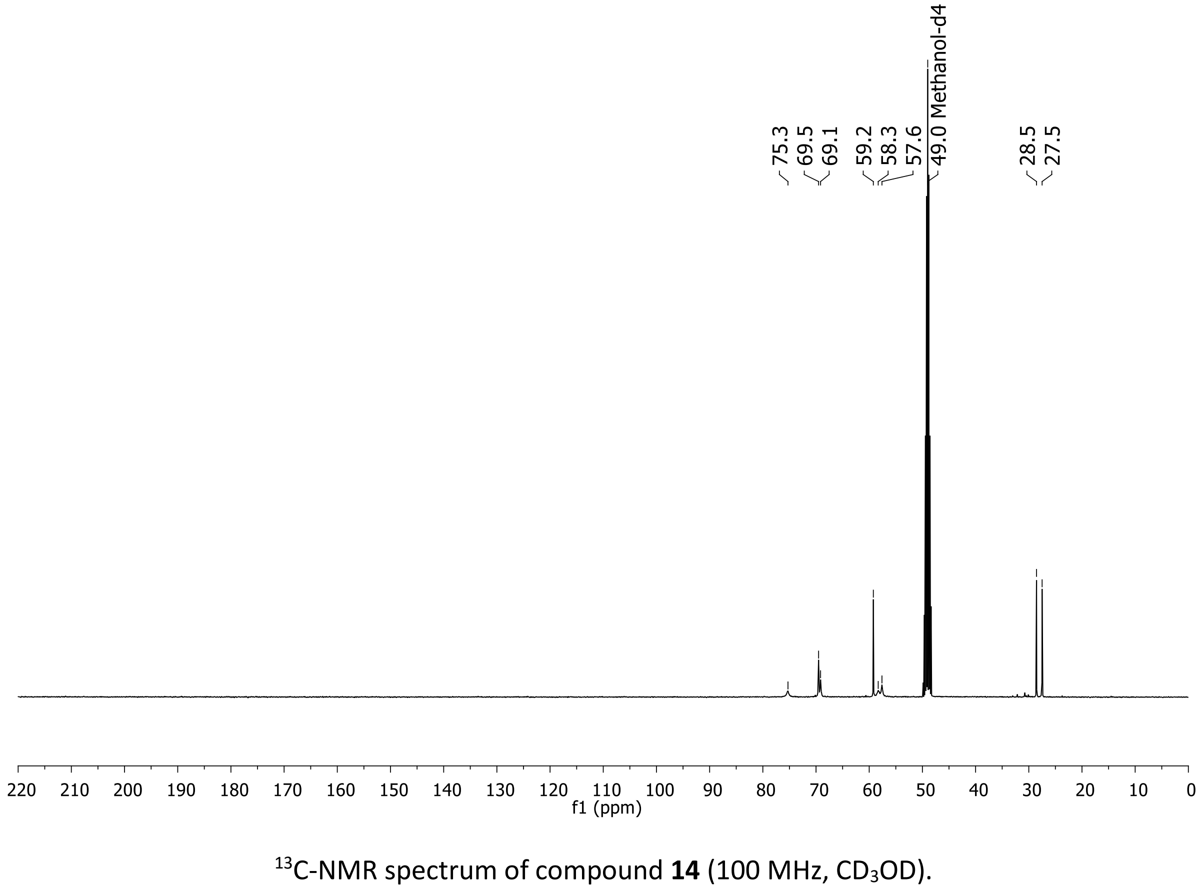

^{13}C-NMR spectrum of compound **14** (100 MHz, CD_3OD).

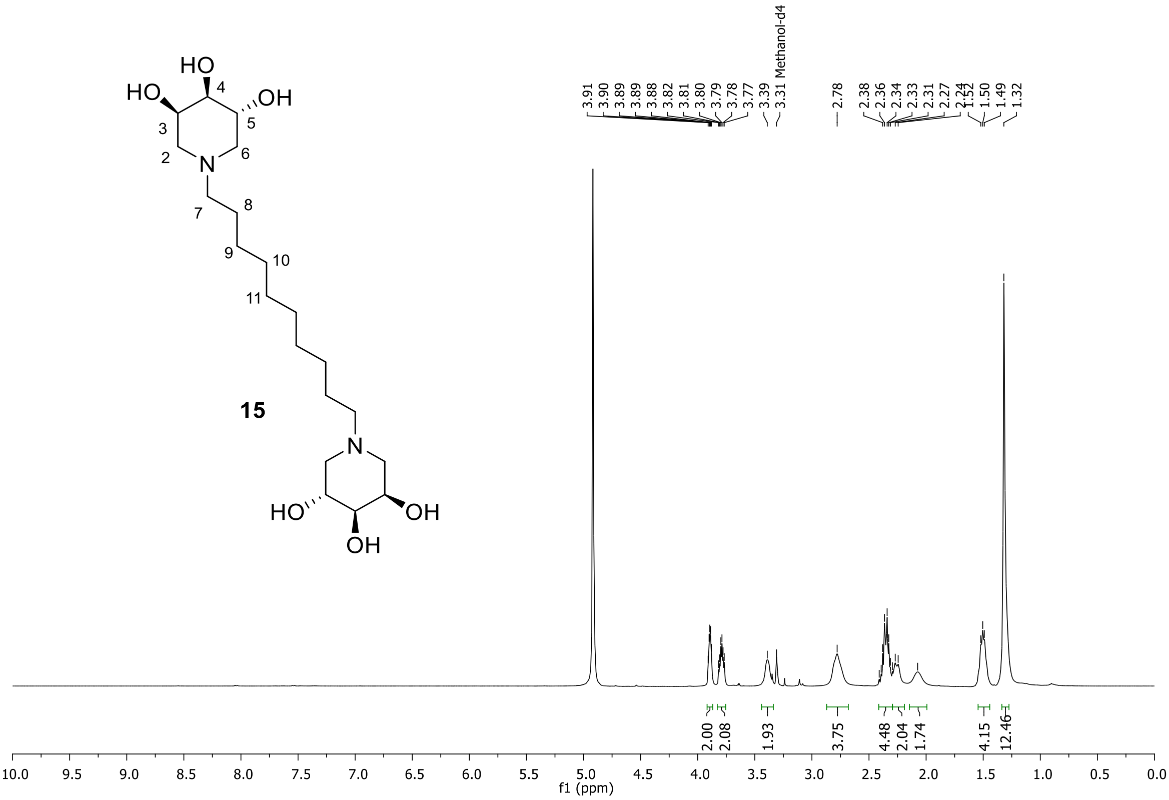

^{1}H-NMR spectrum of compound **15** (400 MHz, CD_3OD).

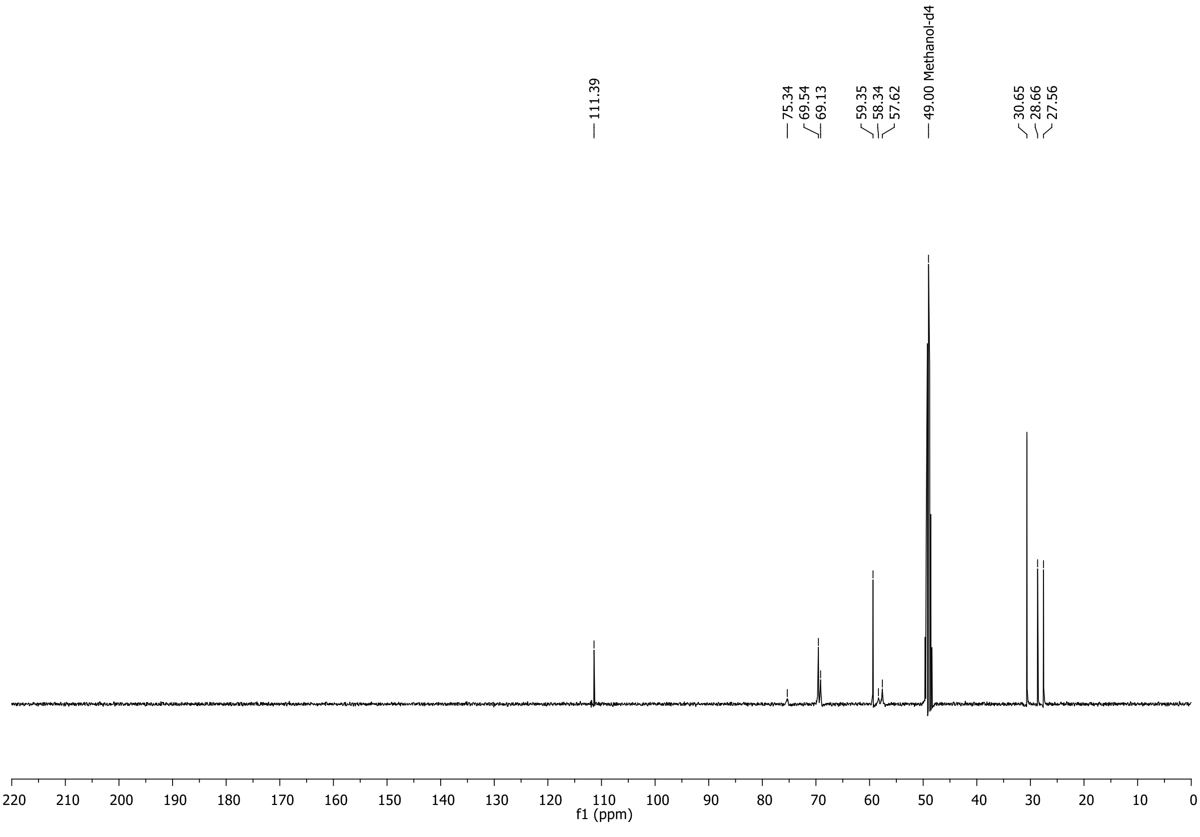

^{13}C-NMR spectrum of compound **15** (100 MHz, CD_3OD).

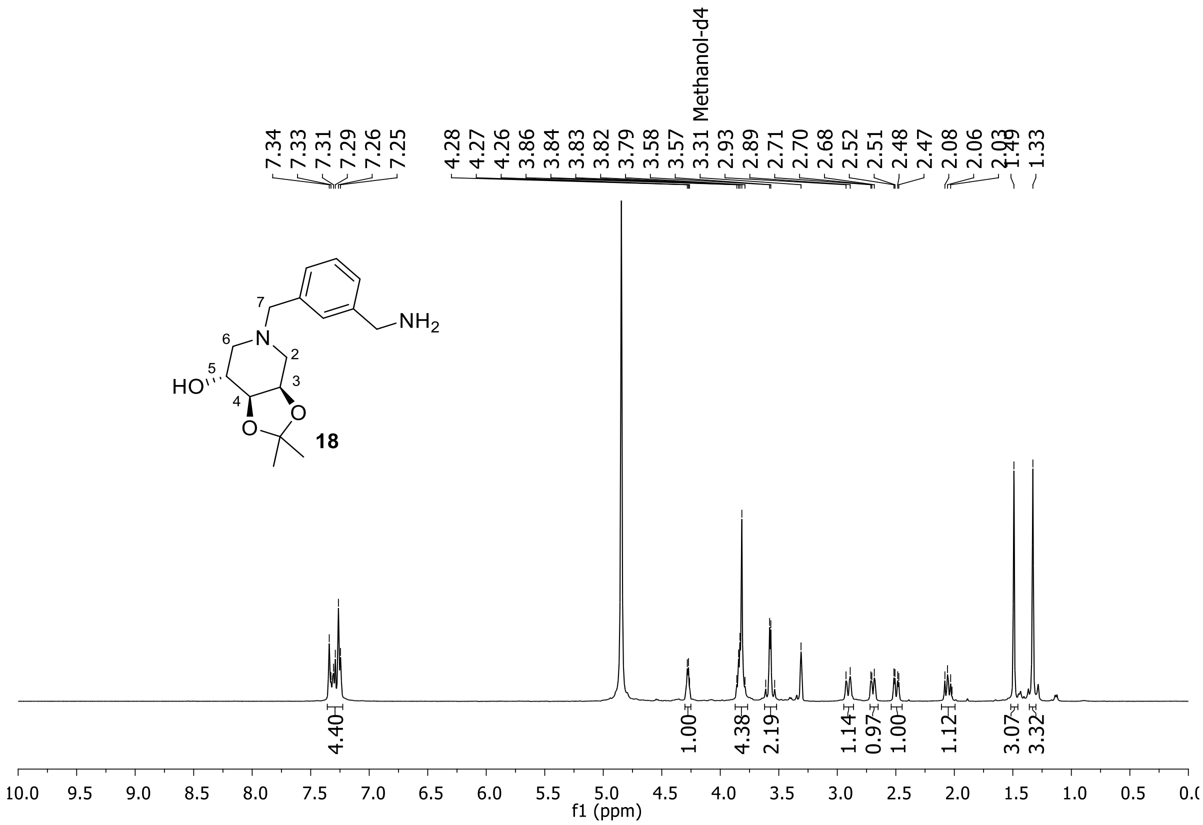

^{1}H-NMR spectrum of compound **18** (400 MHz, CD_3OD).

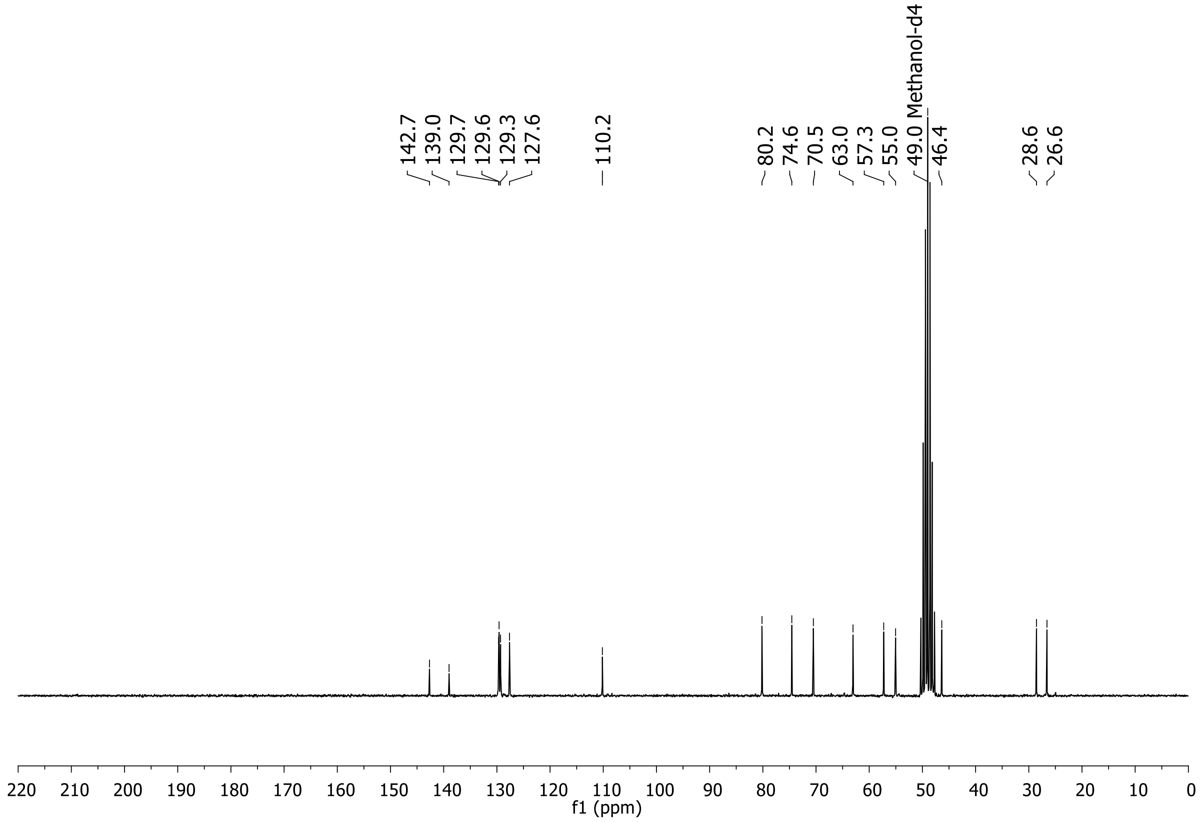

^{13}C-NMR spectrum of compound **18** (50 MHz, CD_3OD).

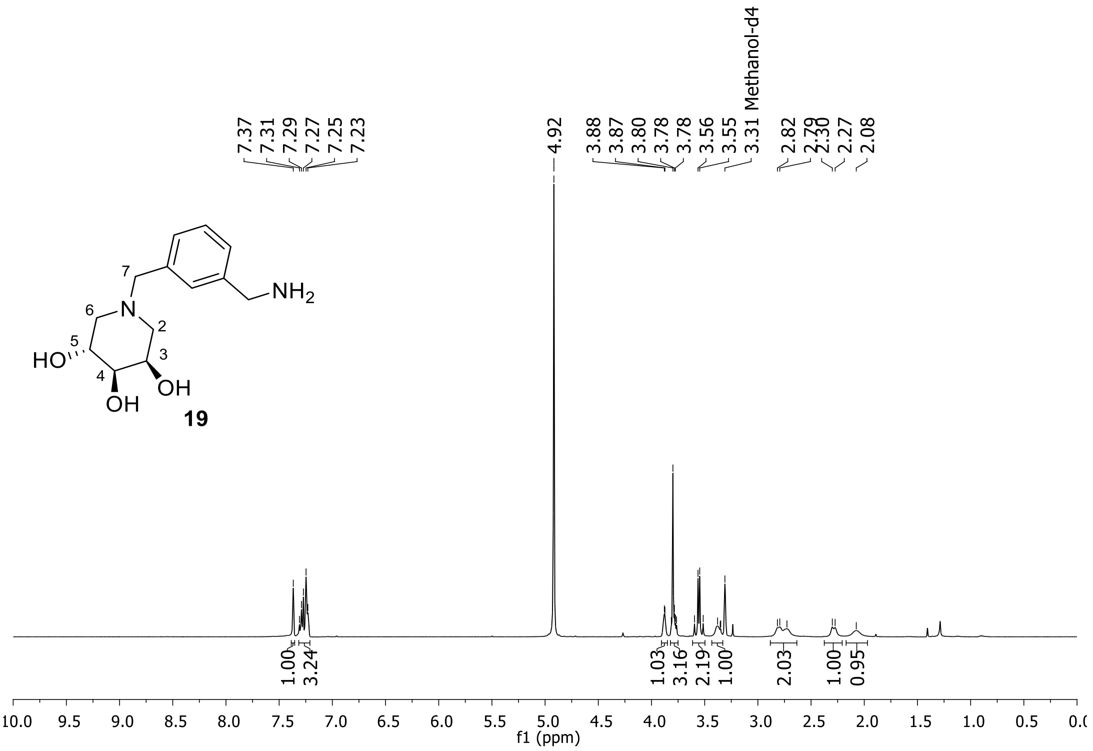

^{1}H-NMR spectrum of compound **19** (400 MHz, CD_3OD).

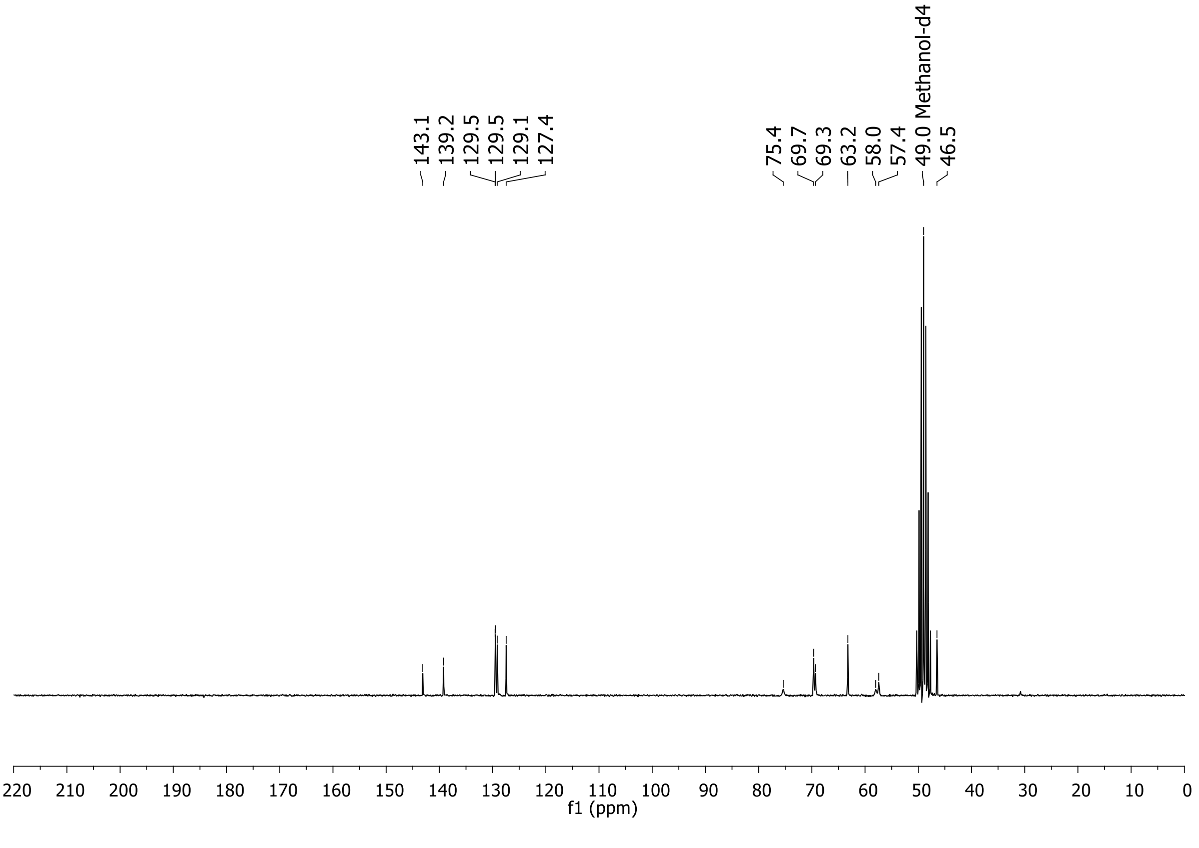

^{13}C-NMR spectrum of compound **19** (50 MHz, CD_3OD).

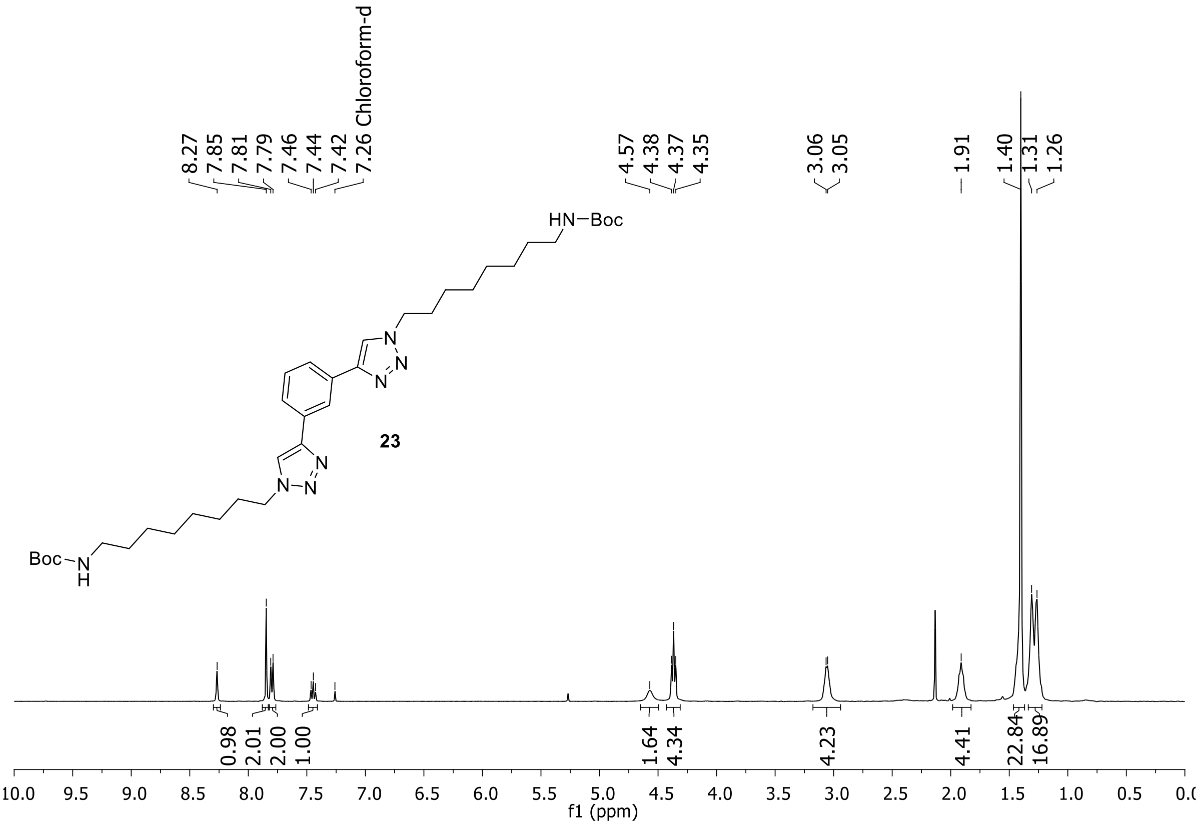

^{1}H-NMR spectrum of compound **23** (400 MHz, $CDCl_3$).

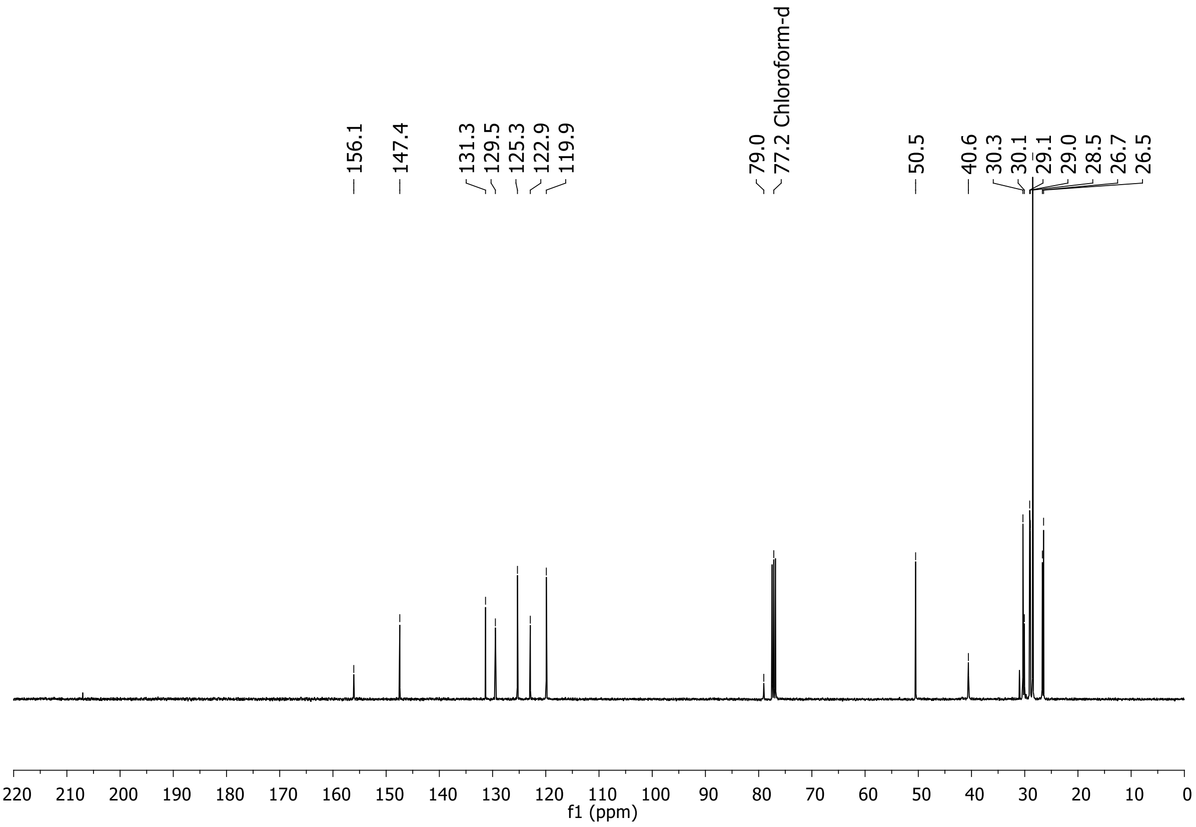

^{13}C-NMR spectrum of compound **23** (100 MHz, $CDCl_3$).

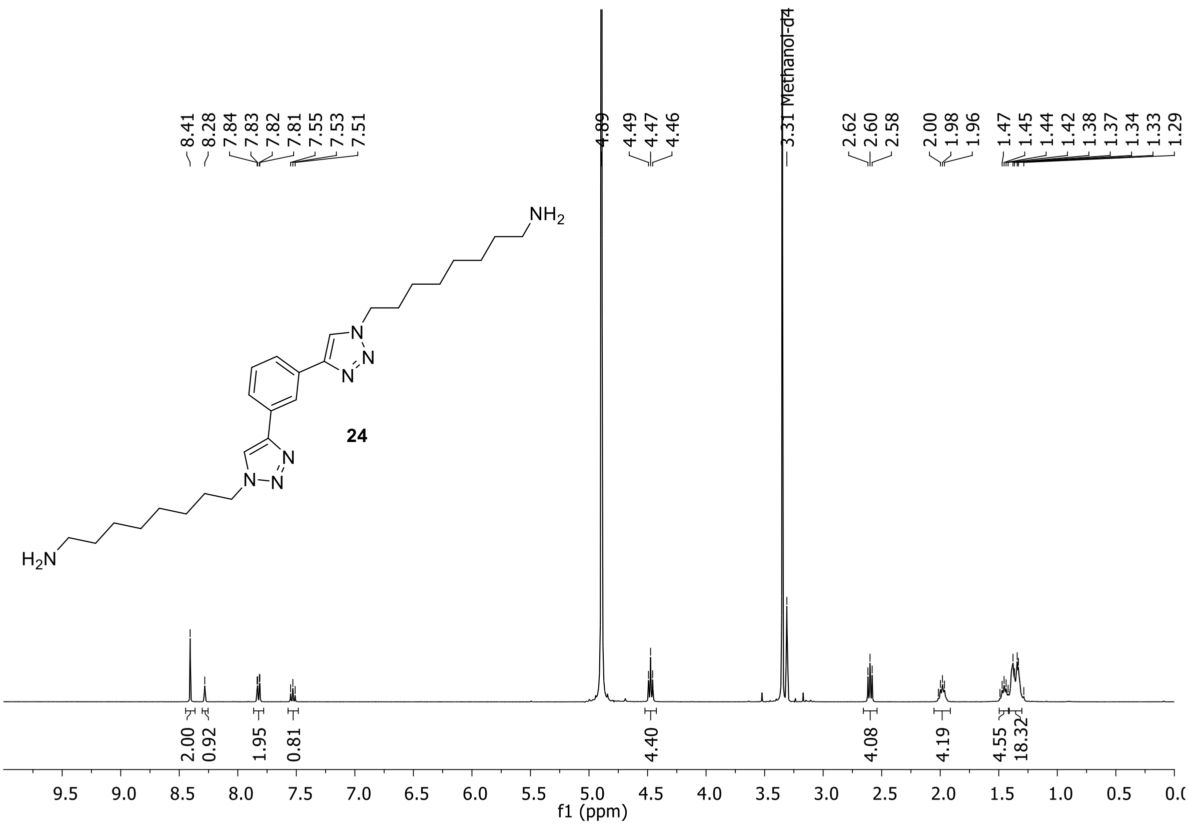

^{1}H-NMR spectrum of compound **24** (400 MHz, CD_3OD).

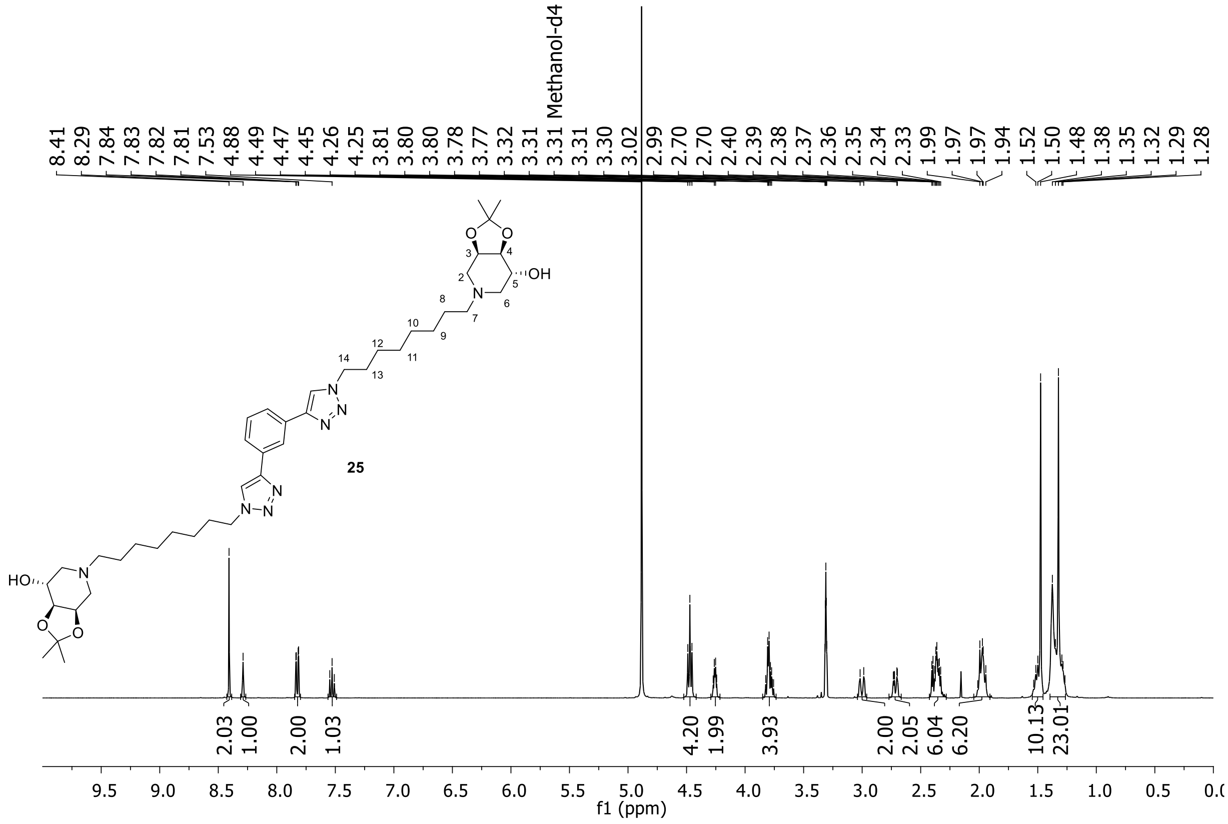

^{1}H-NMR spectrum of compound **25** (400 MHz, CD_3OD).

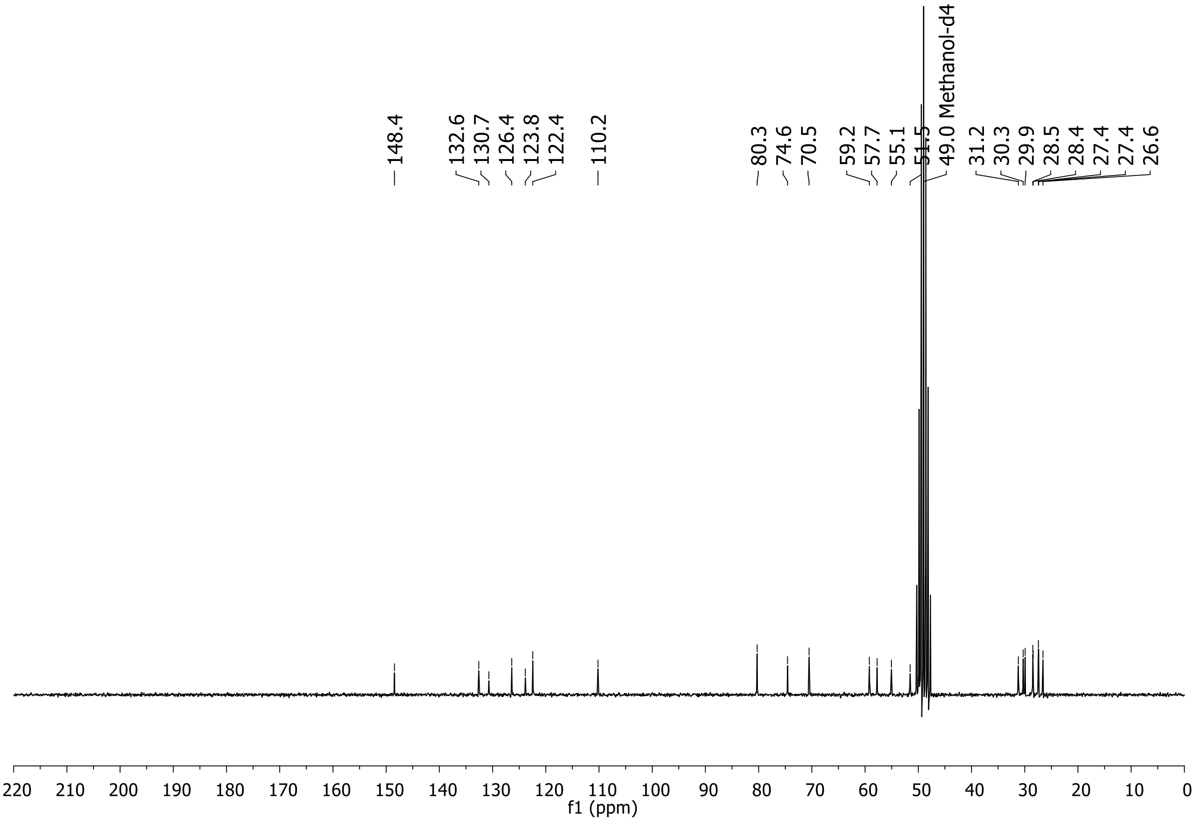

^{13}C-NMR spectrum of compound **25** (50 MHz, CD_3OD).

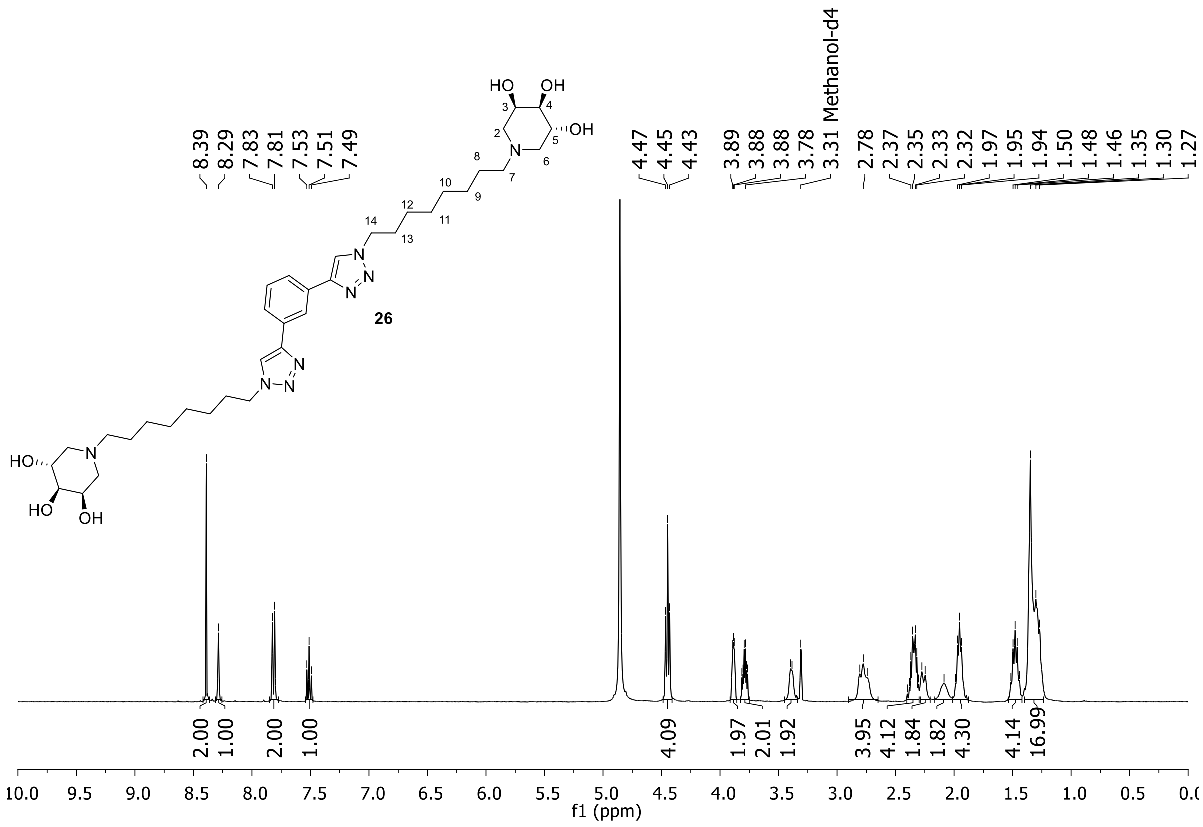

^{1}H-NMR spectrum of compound **26** (400 MHz, CD_3OD).

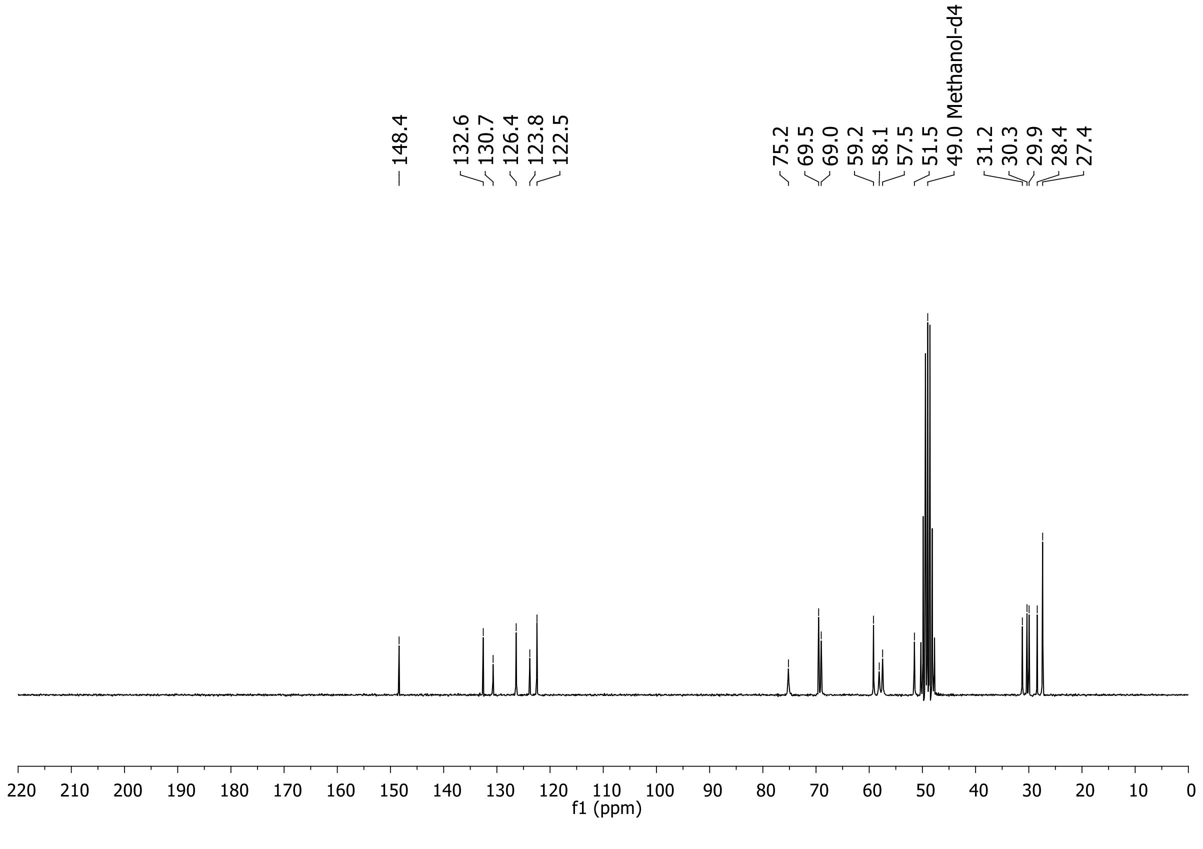

^{13}C-NMR spectrum of compound **26** (50 MHz, CD_3OD).

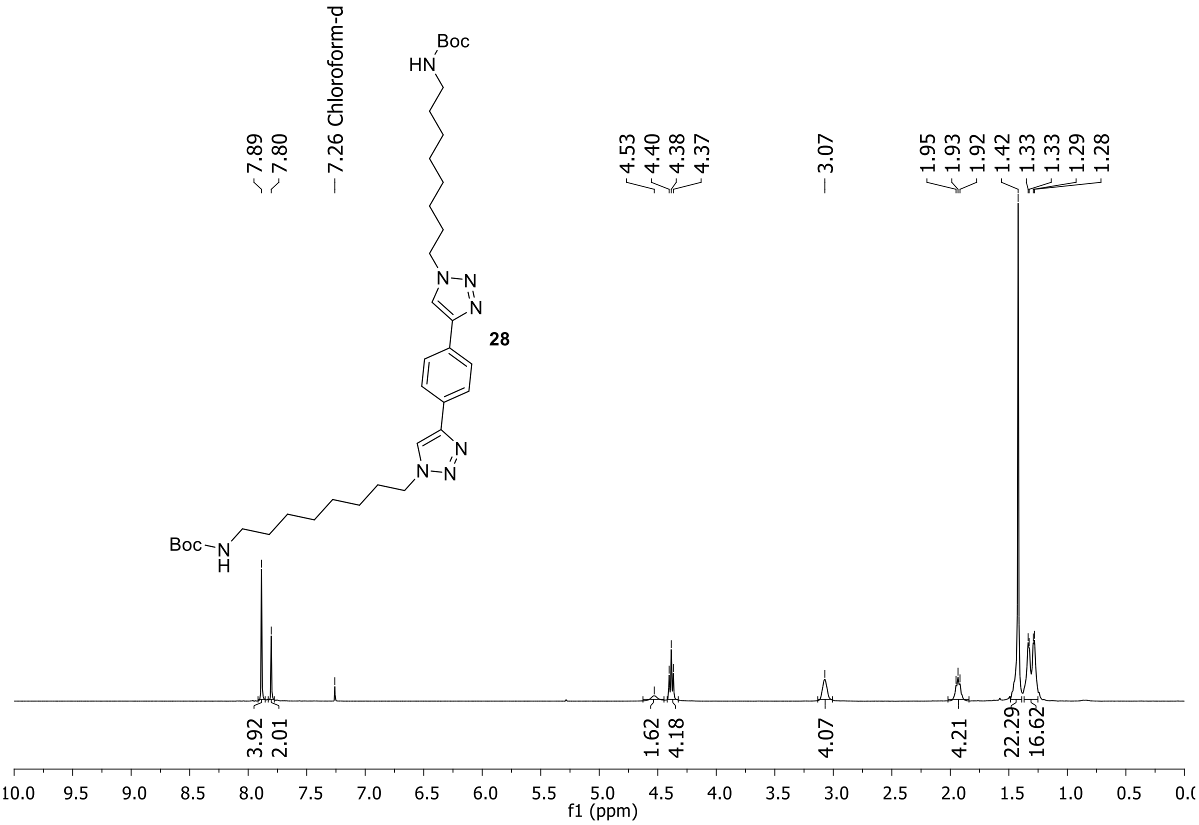

^{1}H-NMR spectrum of compound **28** (400 MHz, $CDCl_3$).

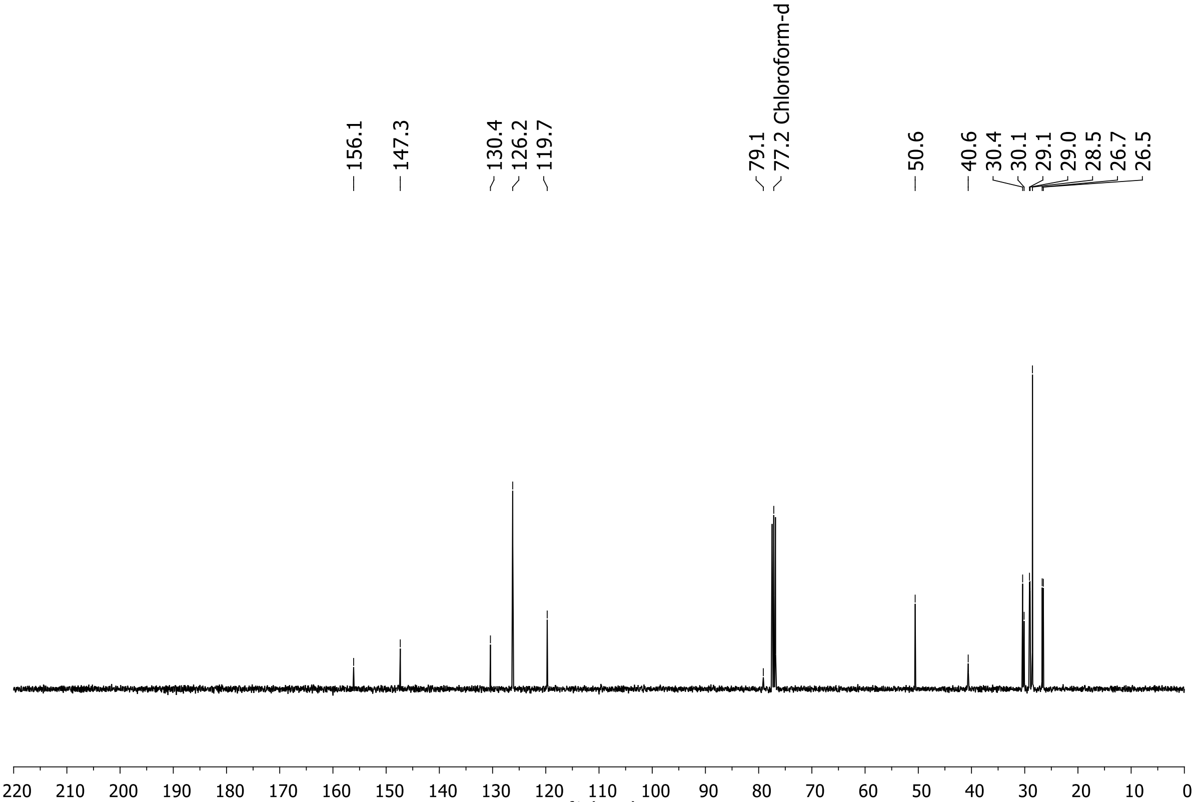

^{13}C-NMR spectrum of compound **28** (100 MHz, $CDCl_3$).

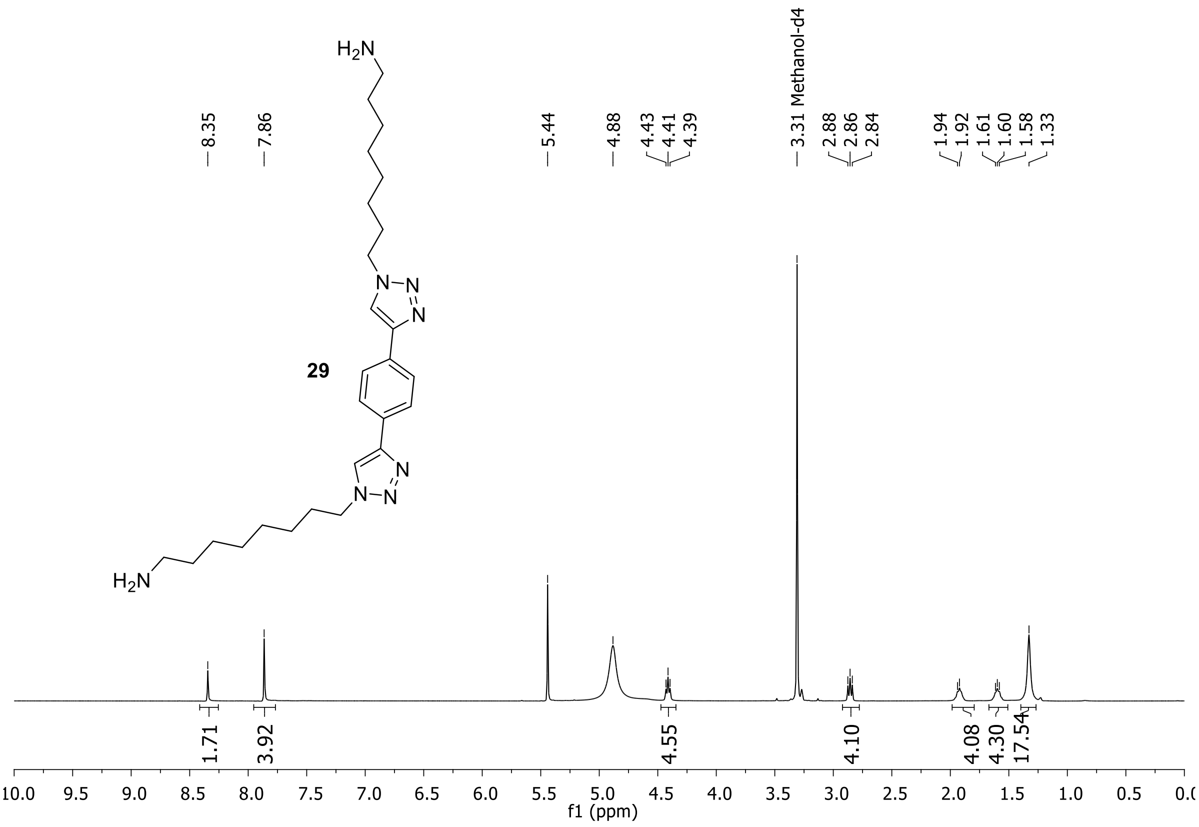

^{1}H-NMR spectrum of compound **29** (400 MHz, CD_3OD).

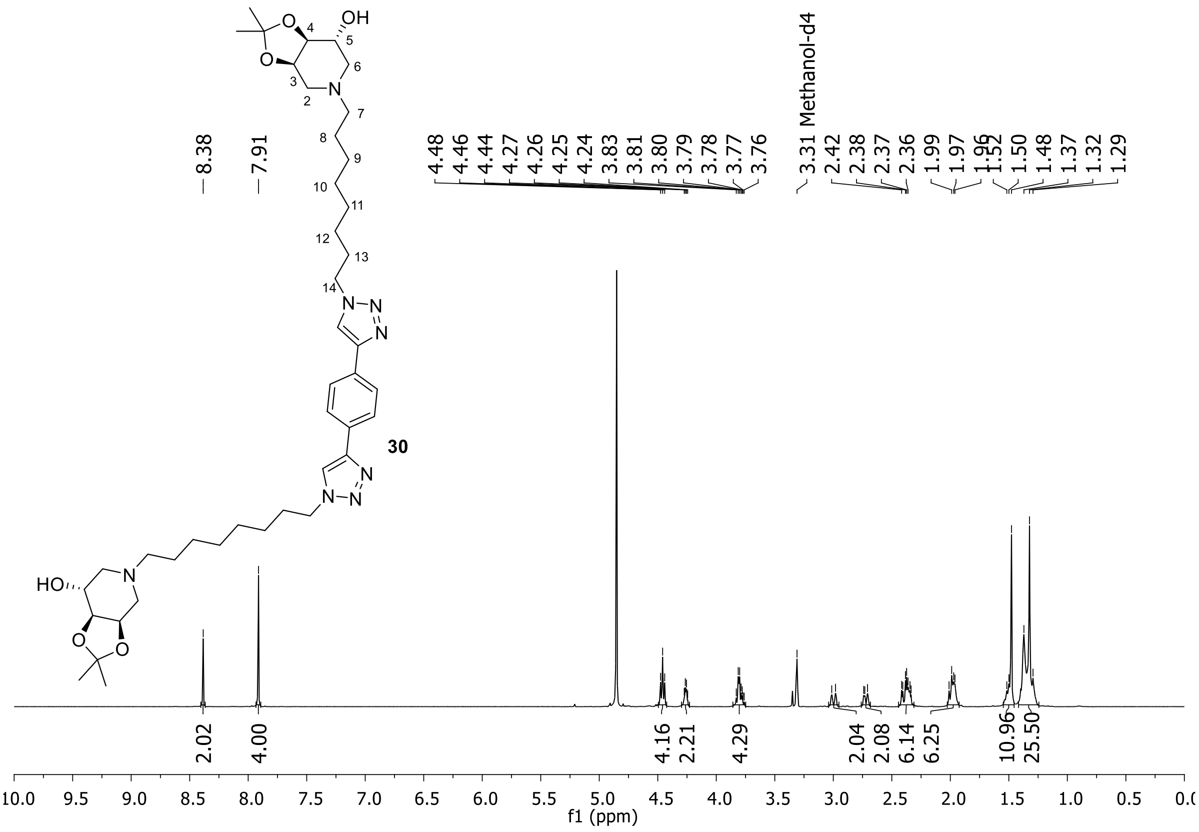

^{1}H-NMR spectrum of compound **30** (400 MHz, CD_3OD).

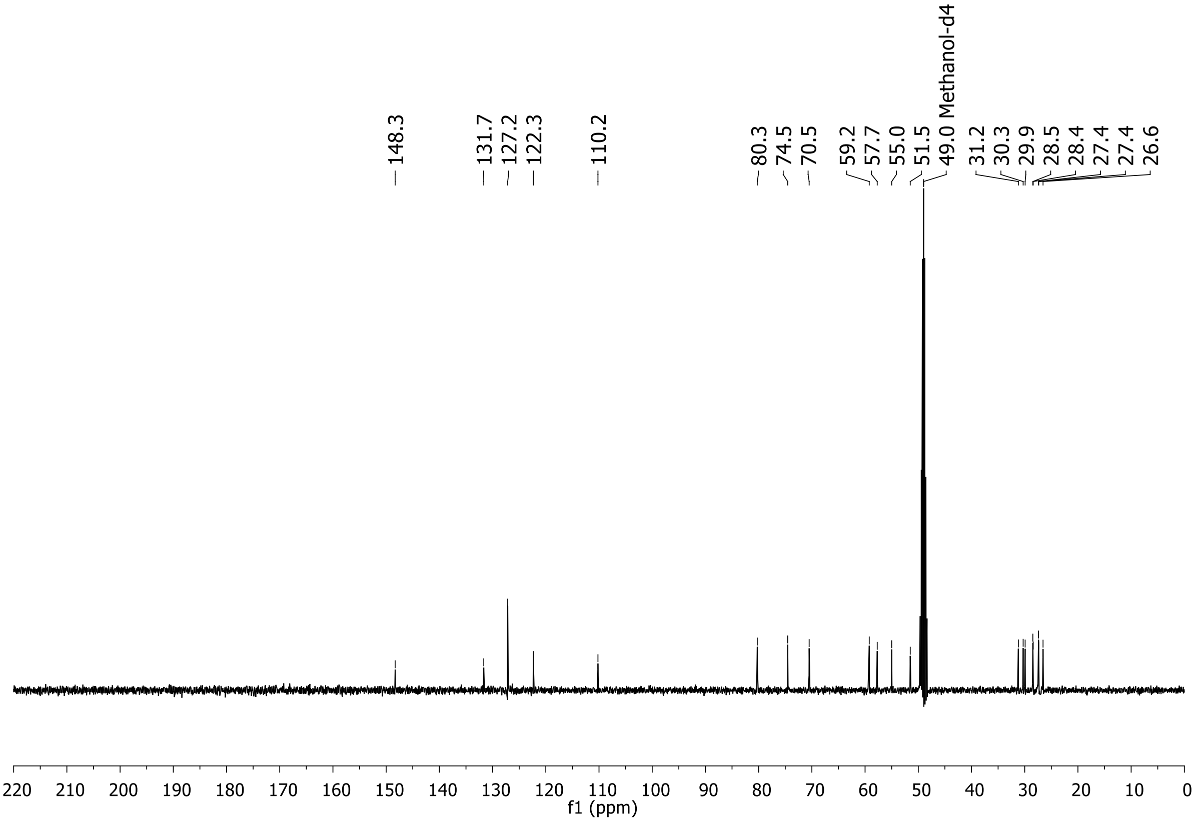

^{13}C-NMR spectrum of compound **30** (100 MHz, CD_3OD).

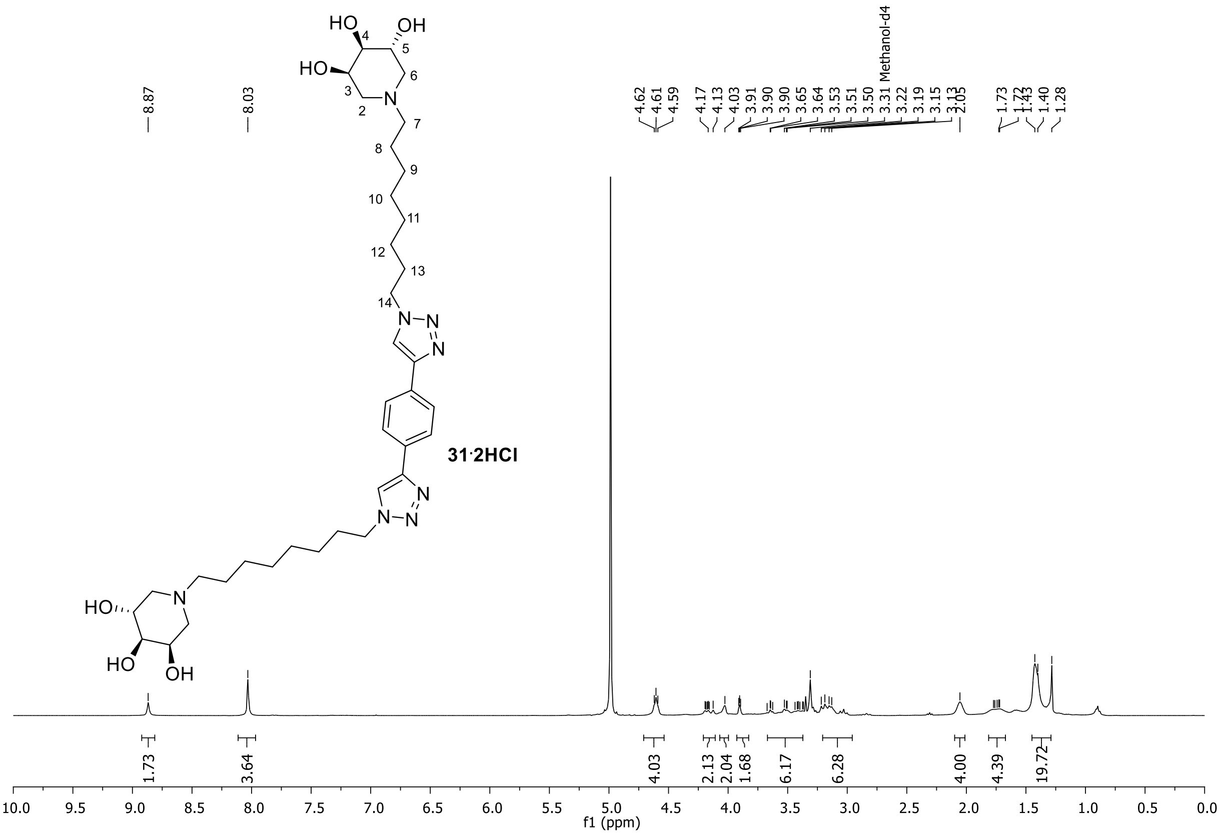

^{1}H NMR spectrum of compound **31·2HCl** (400 MHz, CD_3OD).

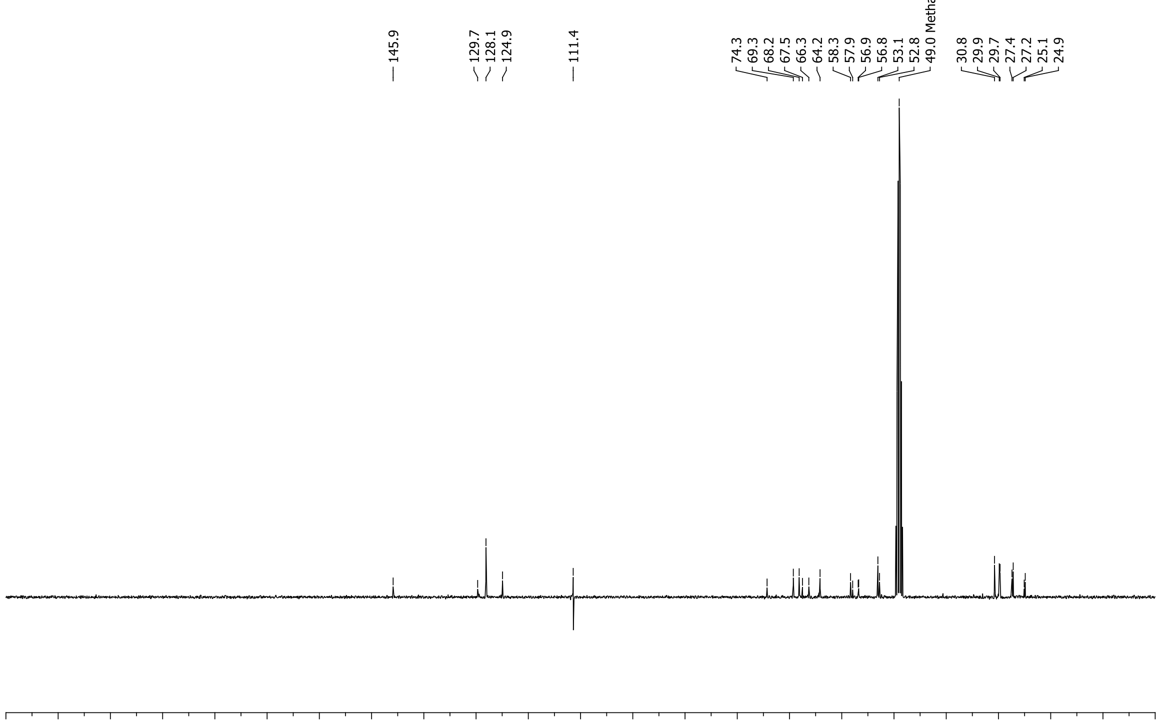

^{13}C NMR spectrum of compound **31·2HCl** (100 MHz, CD_3OD).

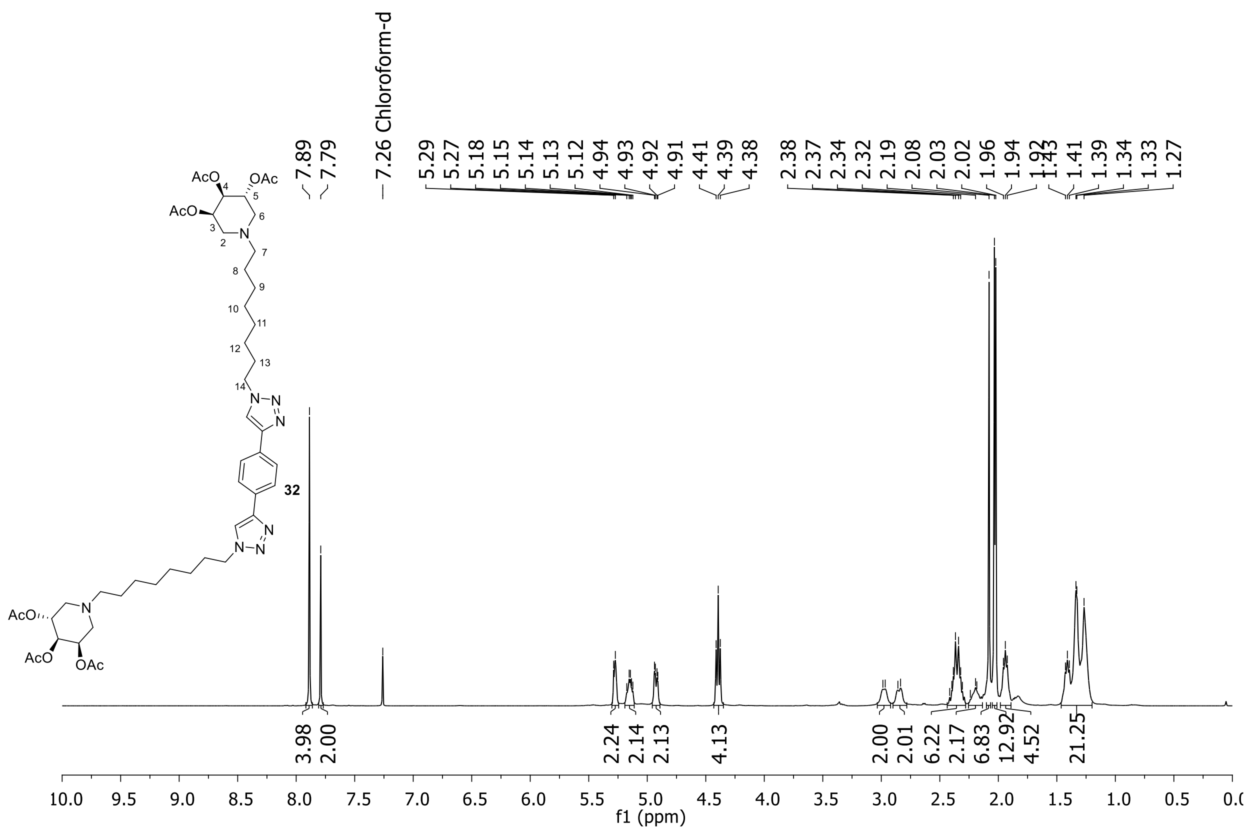

^{1}H-NMR spectrum of compound **32** (400 MHz, $CDCl_3$).

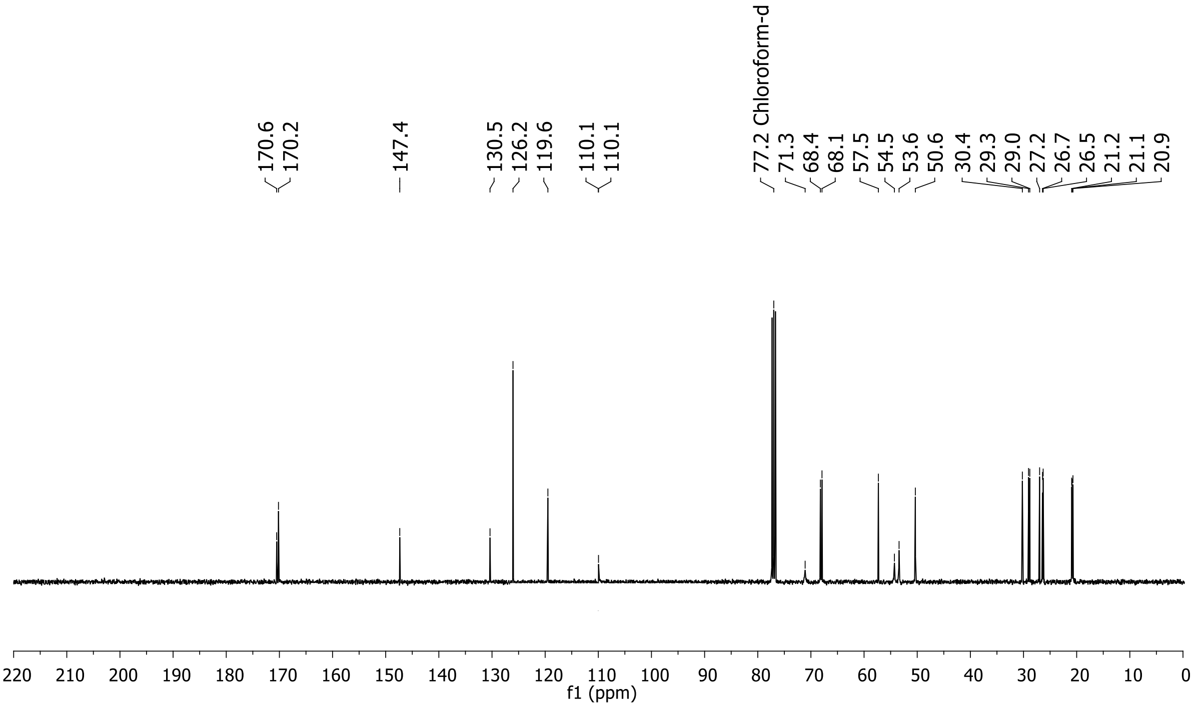

^{13}C-NMR spectrum of compound **32** (100 MHz, $CDCl_3$).

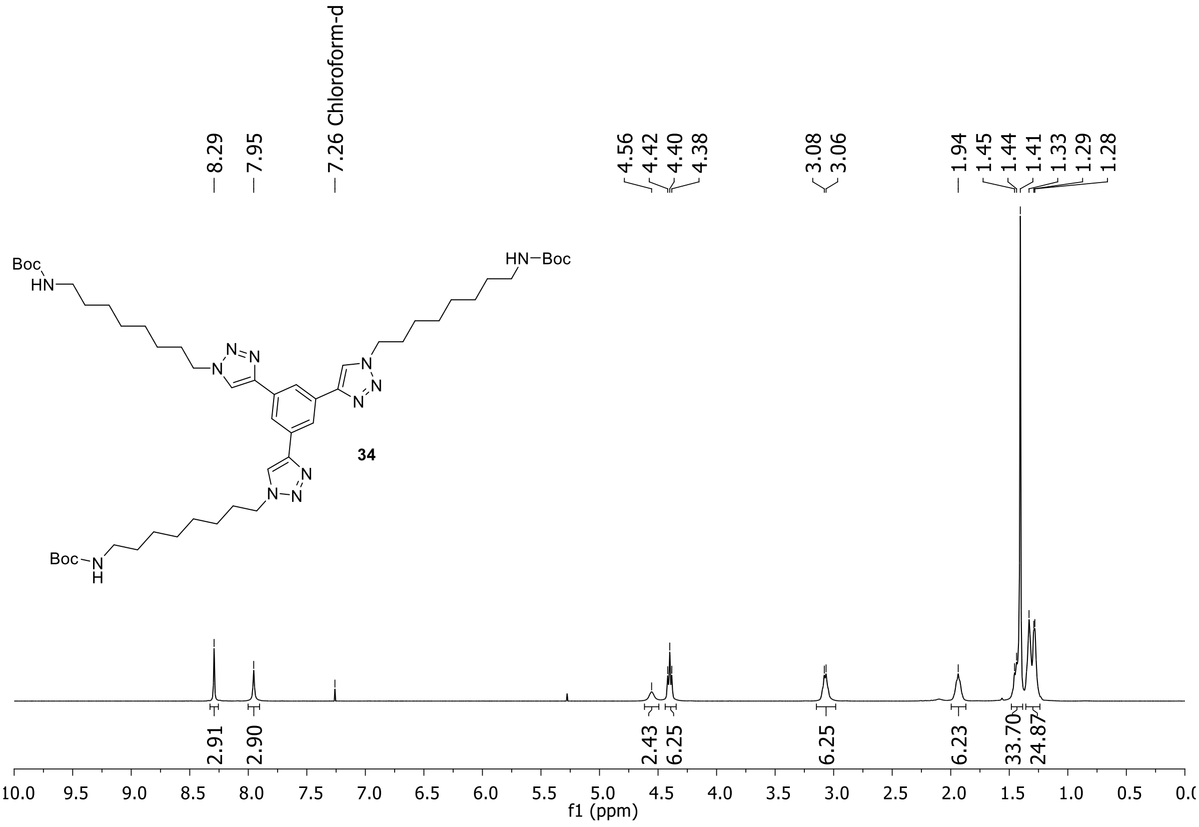

^{1}H-NMR spectrum of compound **34** (400 MHz, $CDCl_3$).

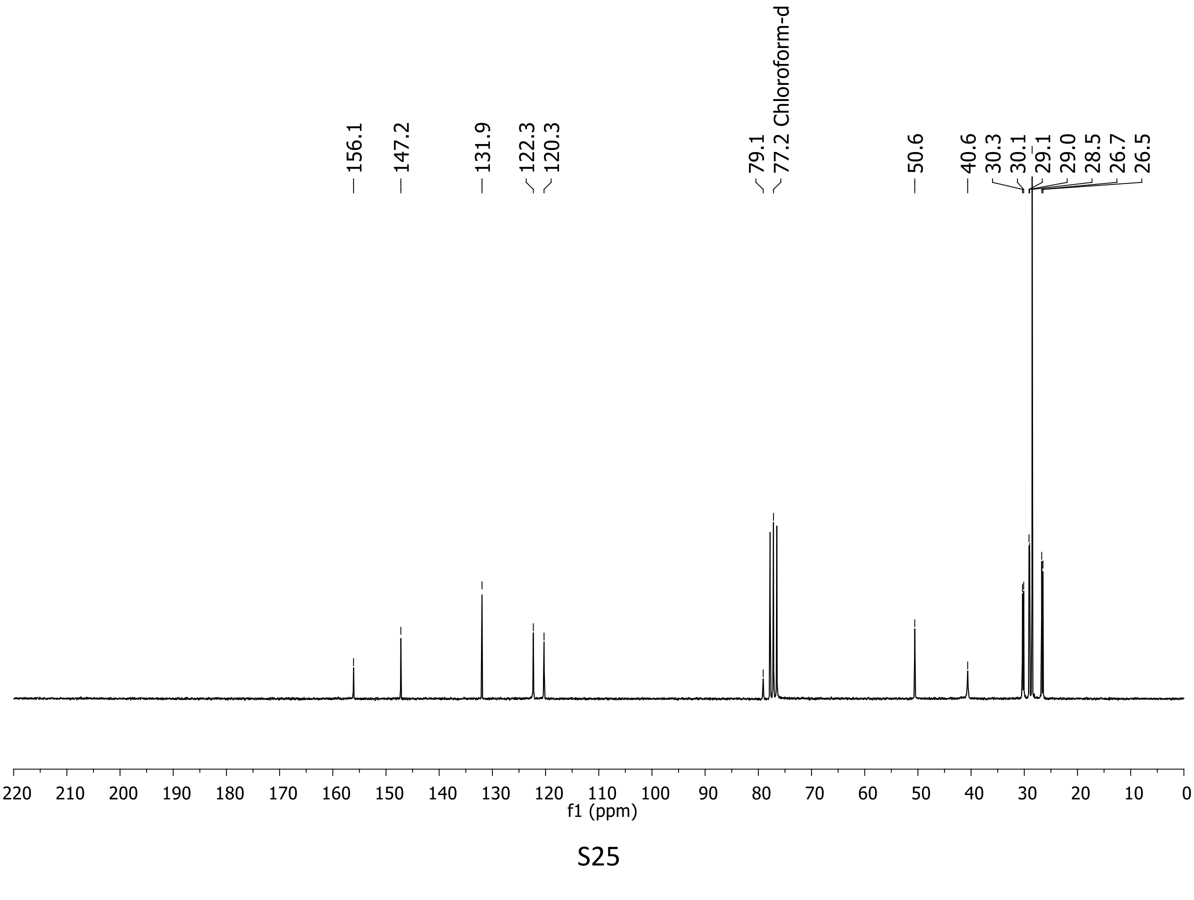

^{13}C-NMR spectrum of compound **34** (50 MHz, $CDCl_3$).

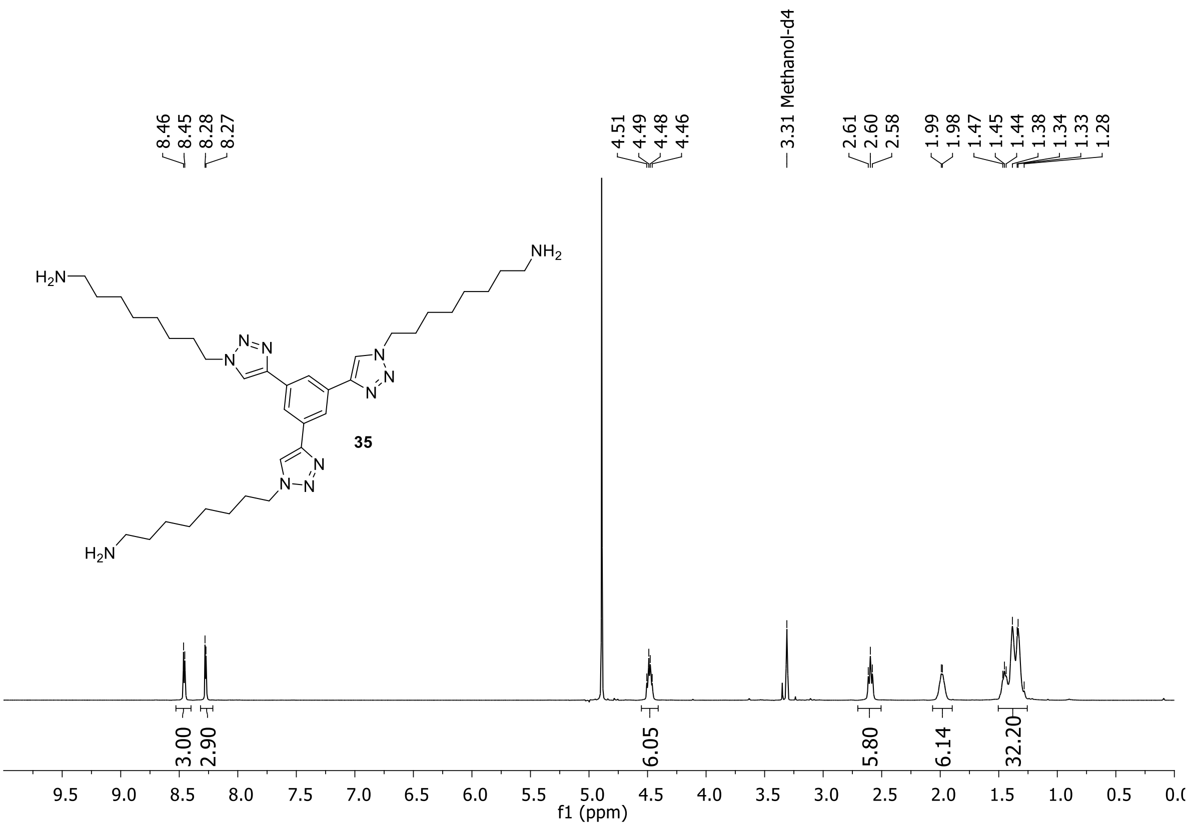

^{1}H-NMR spectrum of compound **35** (400 MHz, CD_3OD).

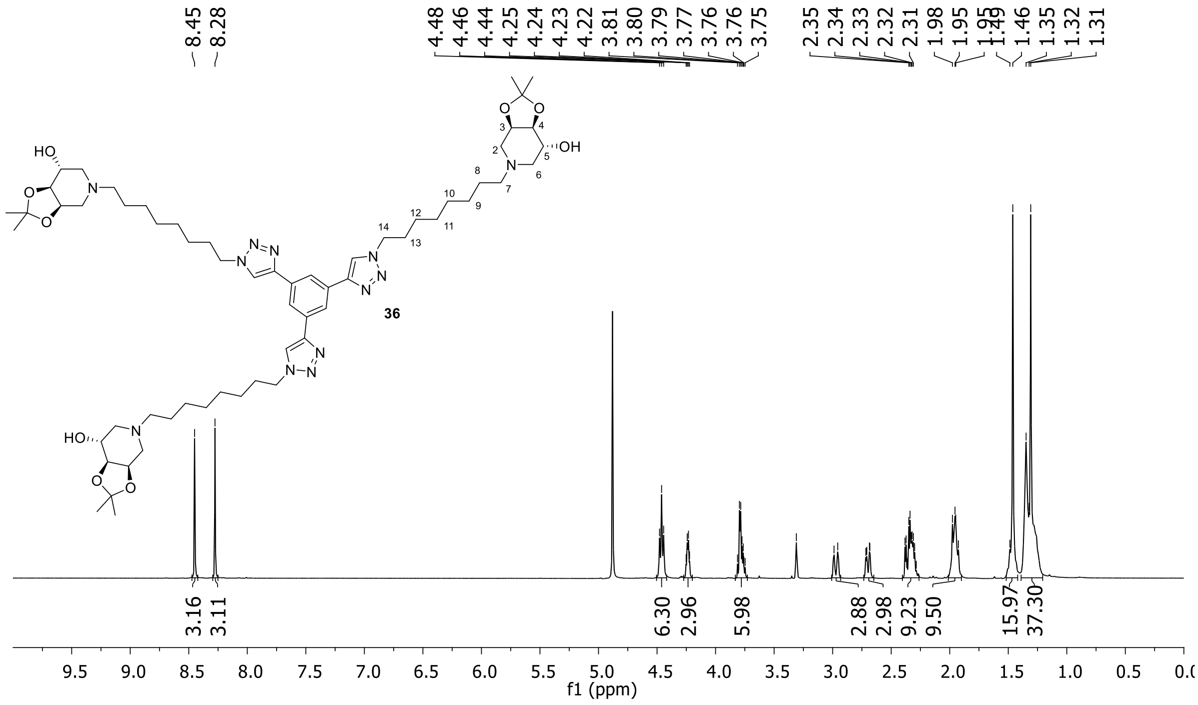

^{1}H-NMR spectrum of compound **36** (400 MHz, CD_3OD).

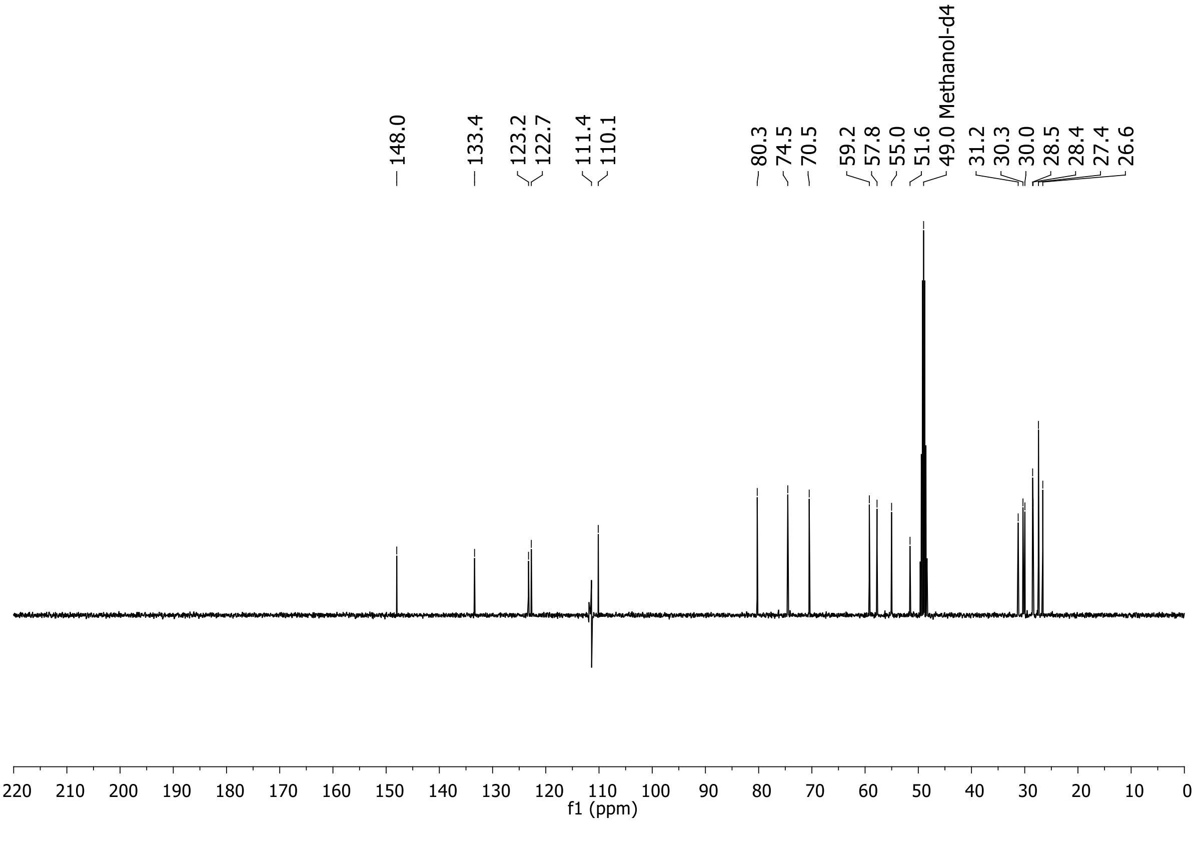

^{13}C-NMR spectrum of compound **36** (100 MHz, CD_3OD).

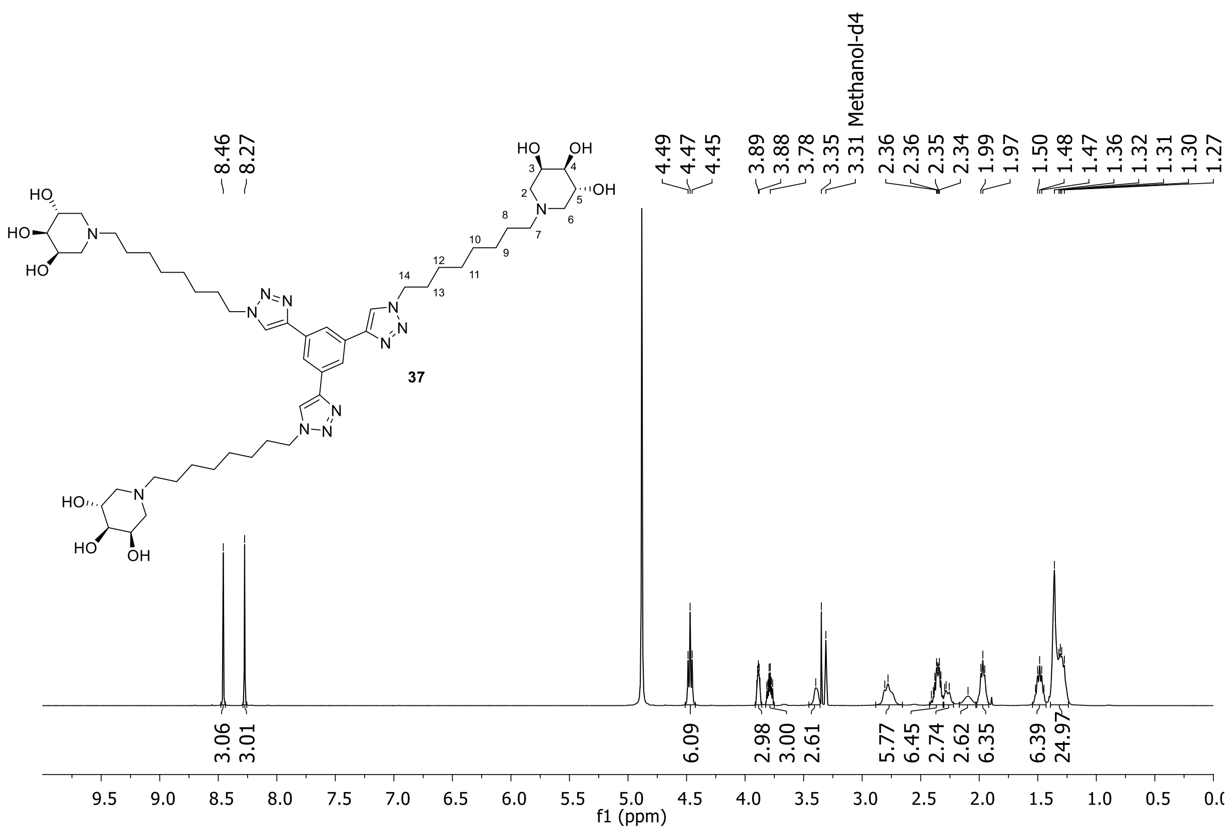

^{1}H-NMR spectrum of compound **37** (400 MHz, CD_3OD).

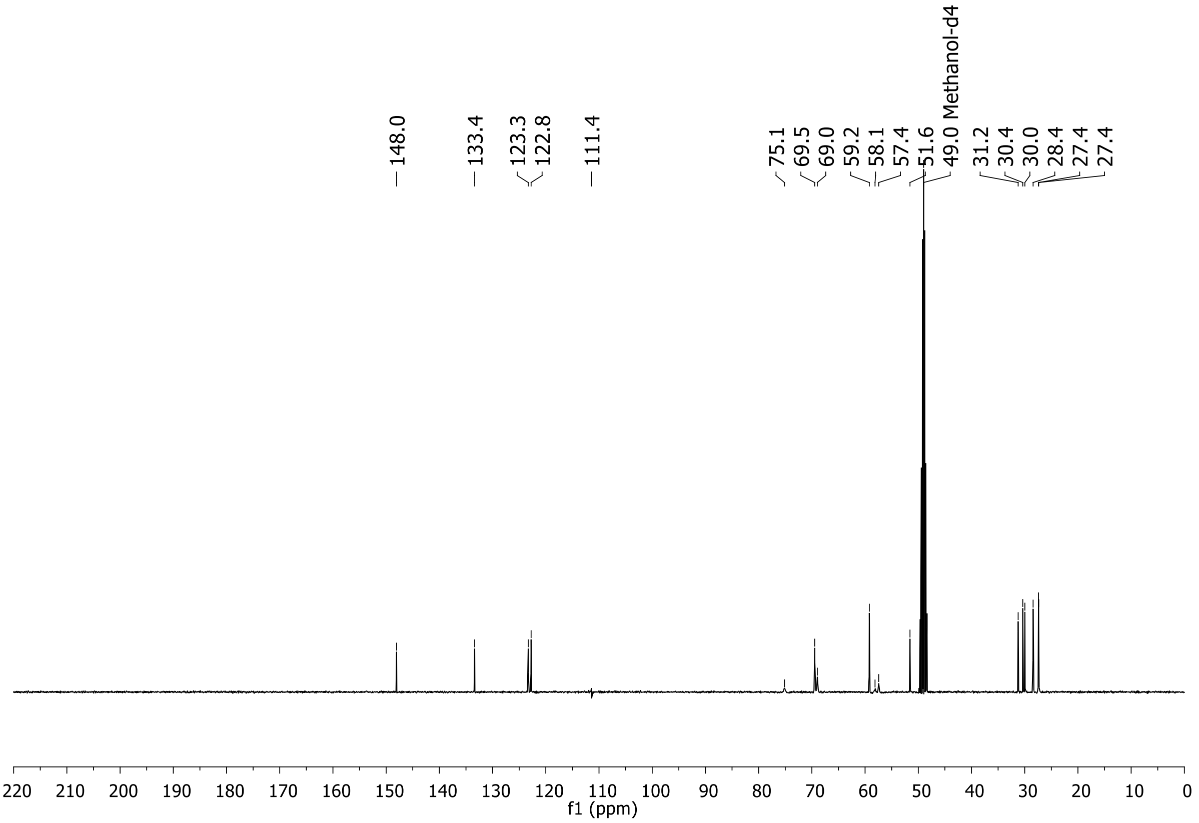

^{13}C-NMR spectrum of compound **37** (100 MHz, CD_3OD).

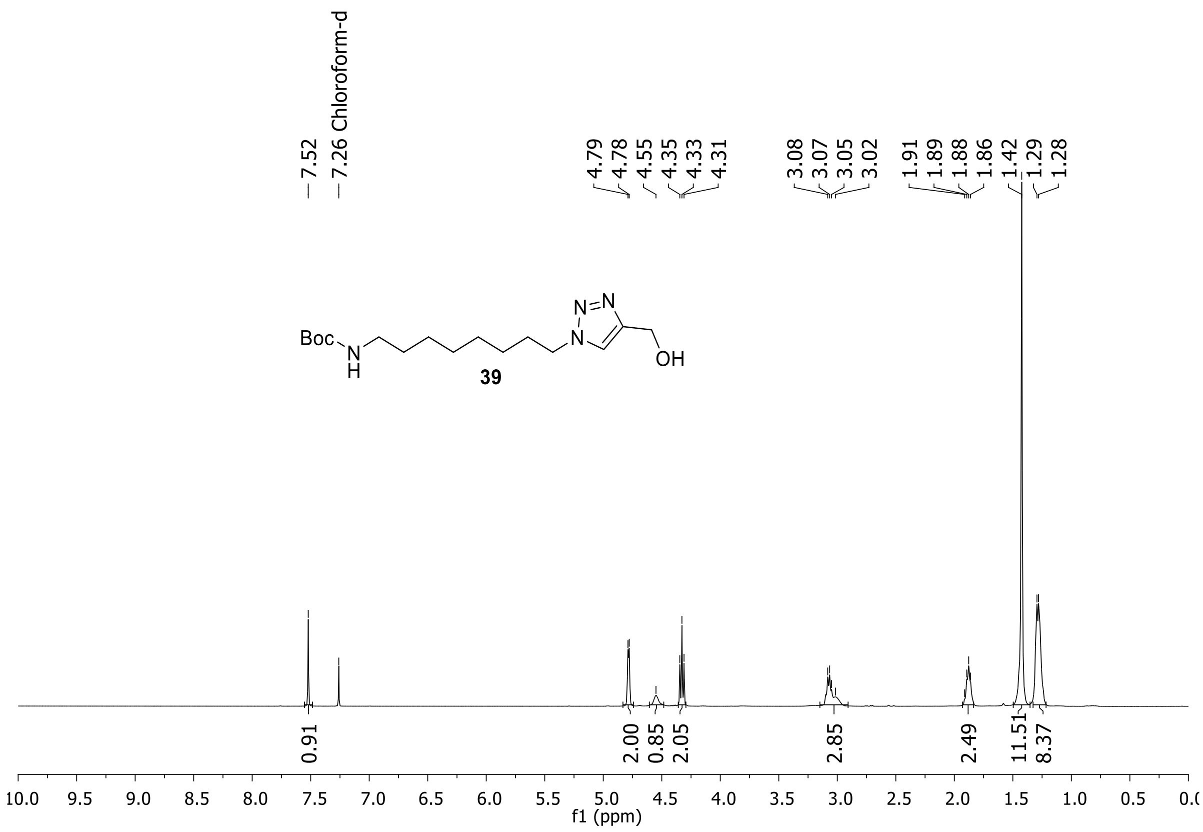

^{1}H-NMR spectrum of compound **39** (400 MHz, $CDCl_3$).

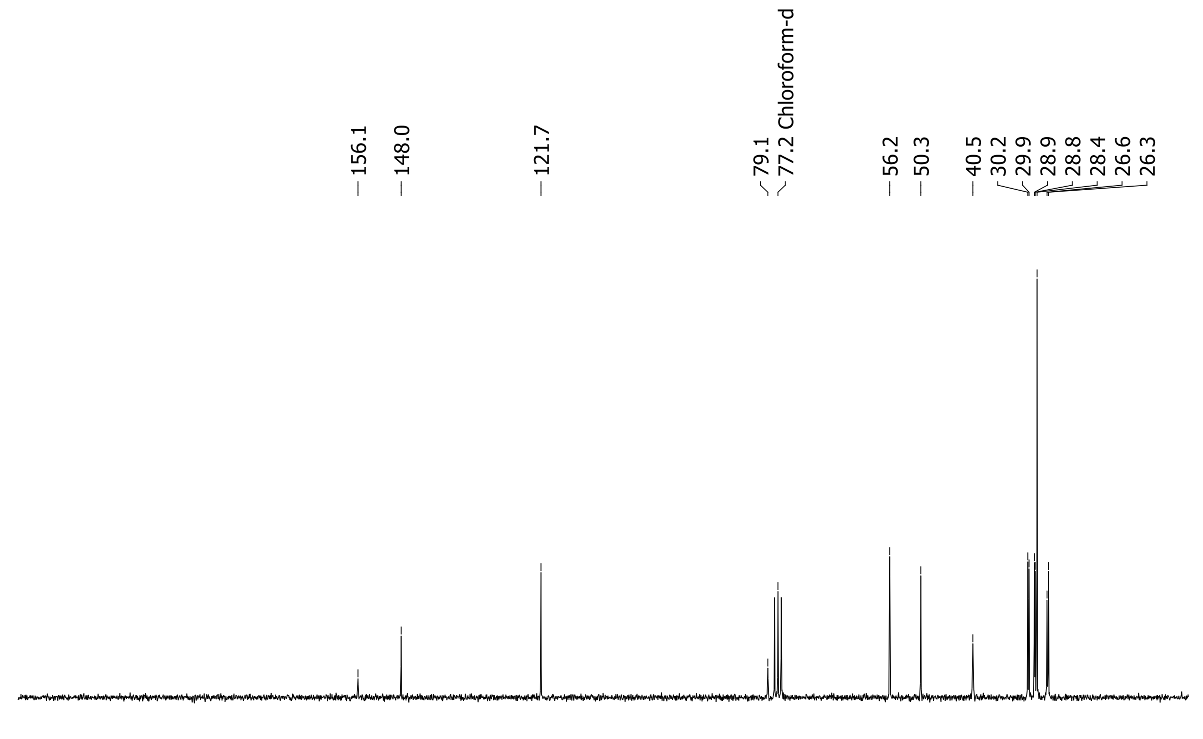

^{13}C-NMR spectrum of compound **39** (50 MHz, $CDCl_3$).

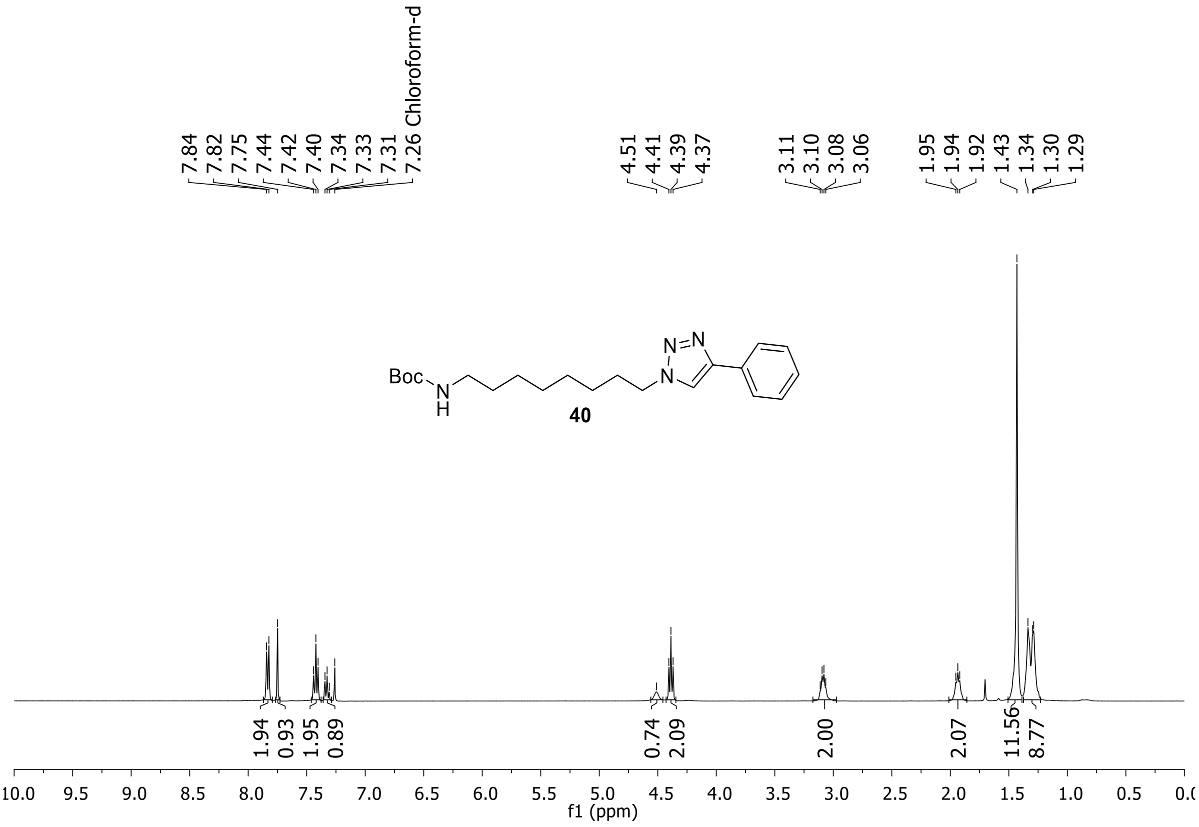

^{1}H-NMR spectrum of compound **40** (400 MHz, $CDCl_3$).

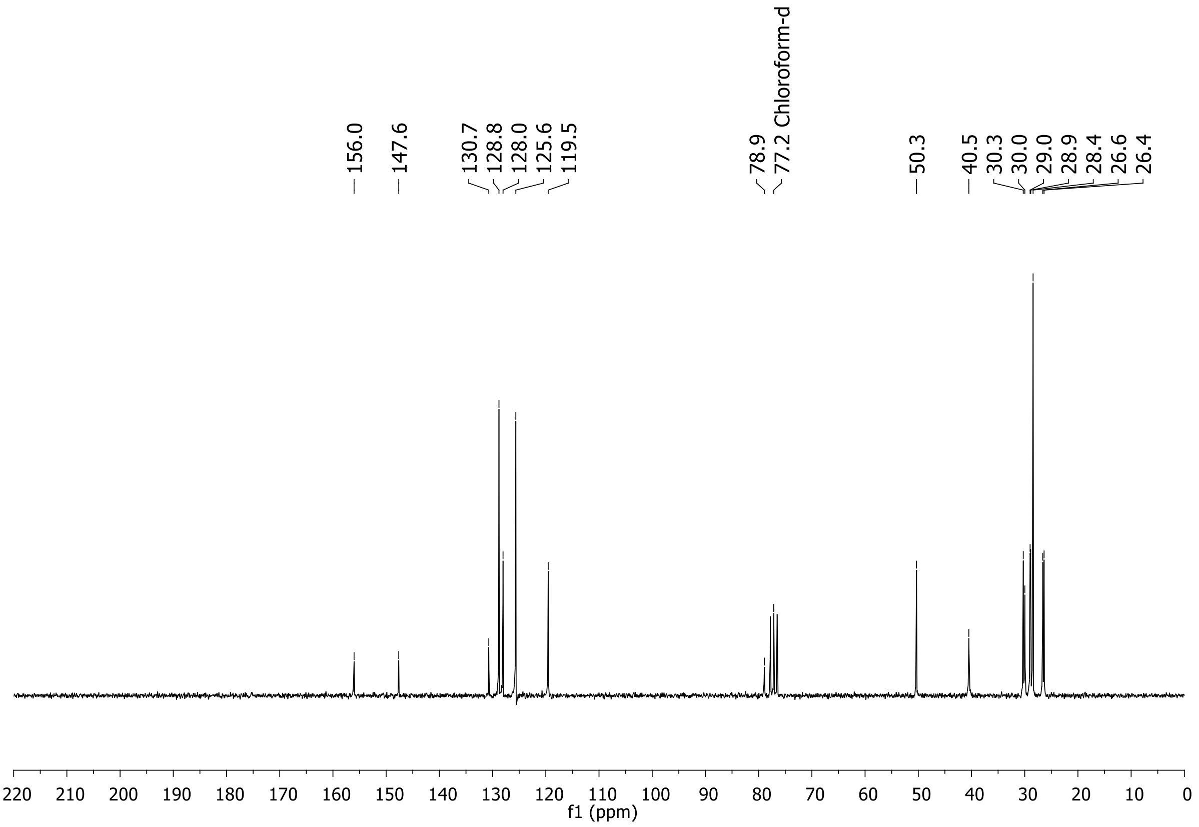

^{13}C-NMR spectrum of compound **40** (50 MHz, $CDCl_3$).

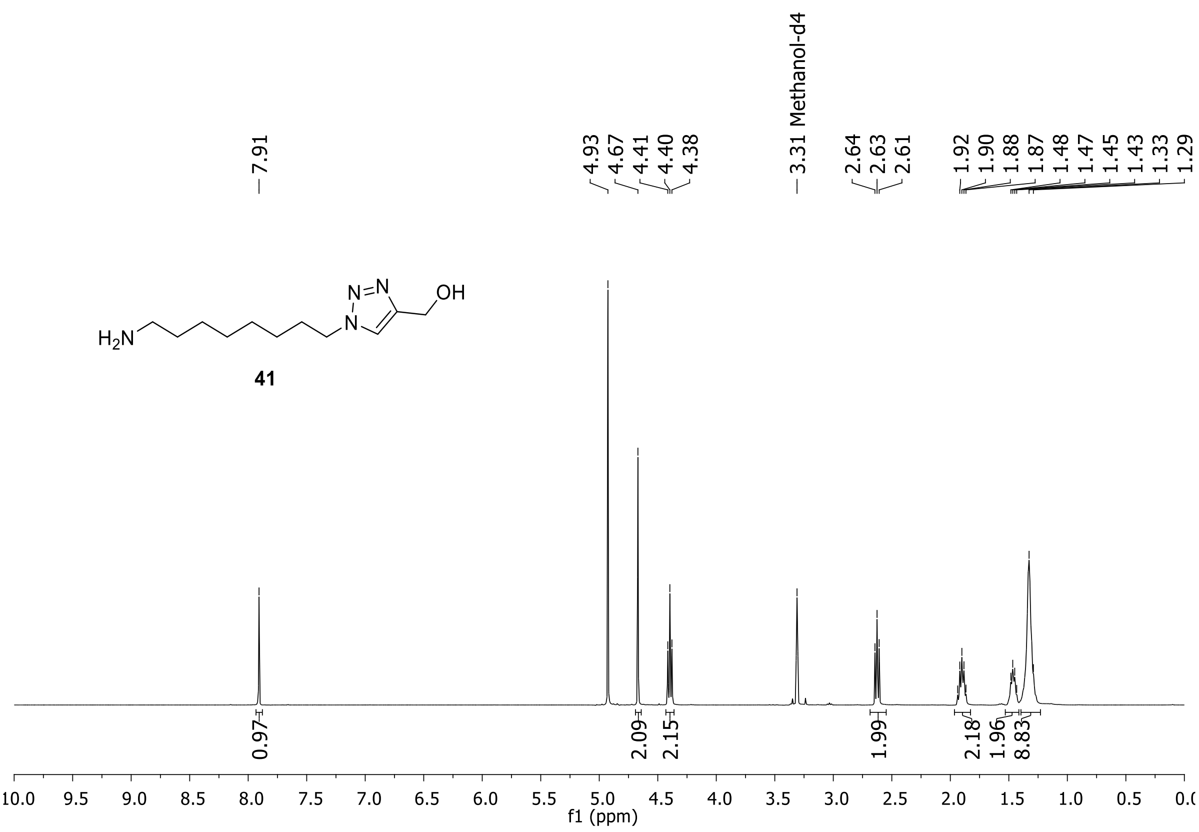

^{1}H-NMR spectrum of compound **41** (400 MHz, CD_3OD).

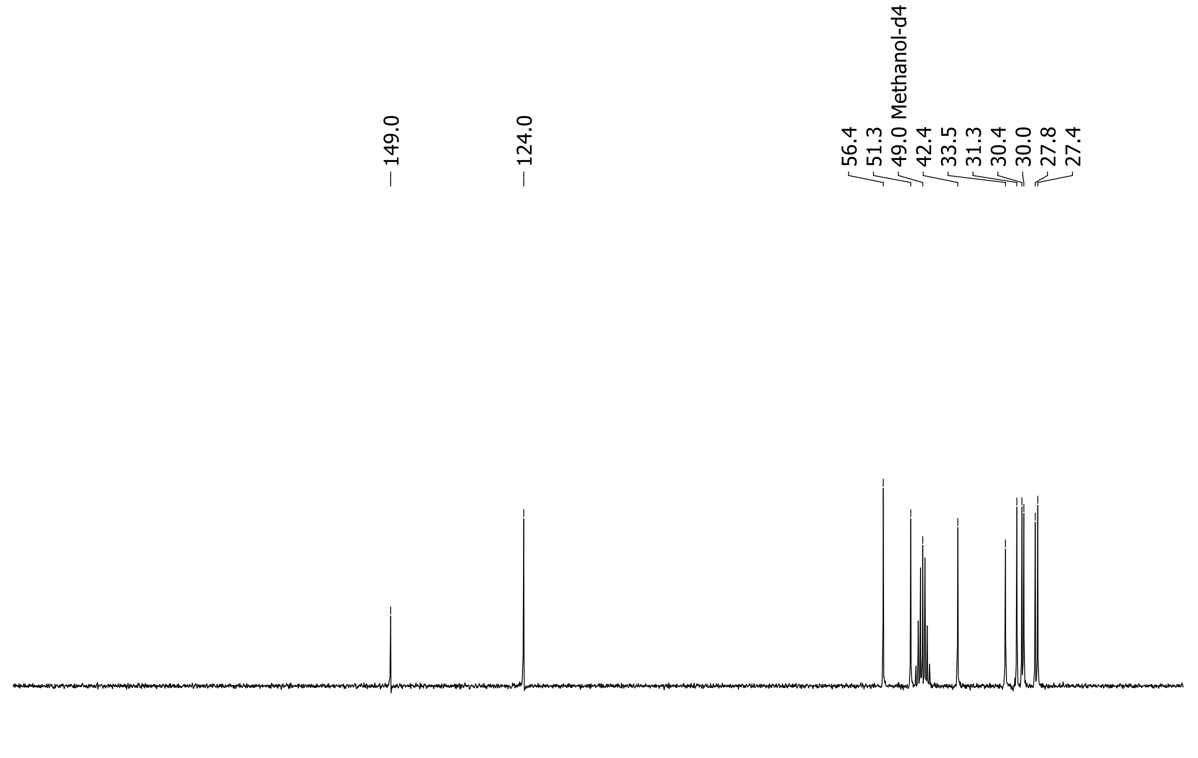

^{13}C-NMR spectrum of compound **41** (50 MHz, CD_3OD).

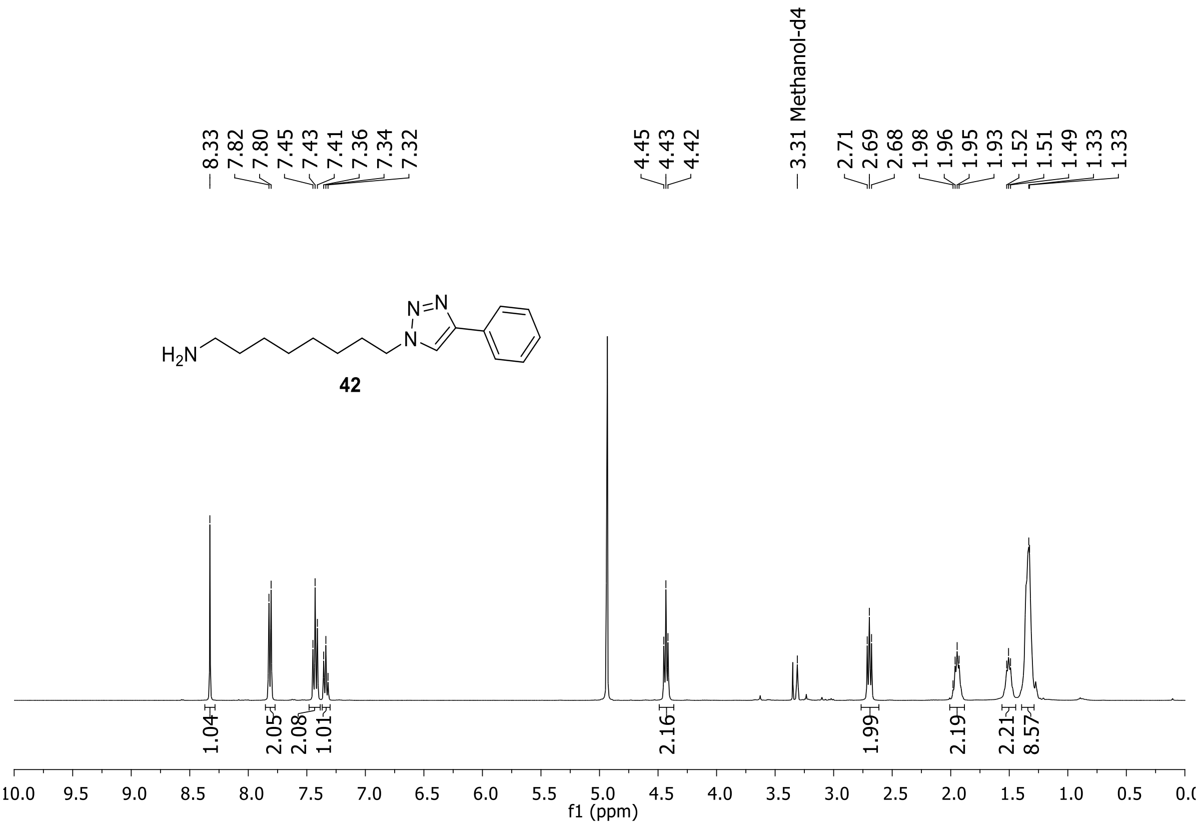

^{1}H-NMR spectrum of compound **42** (400 MHz, CD_3OD).

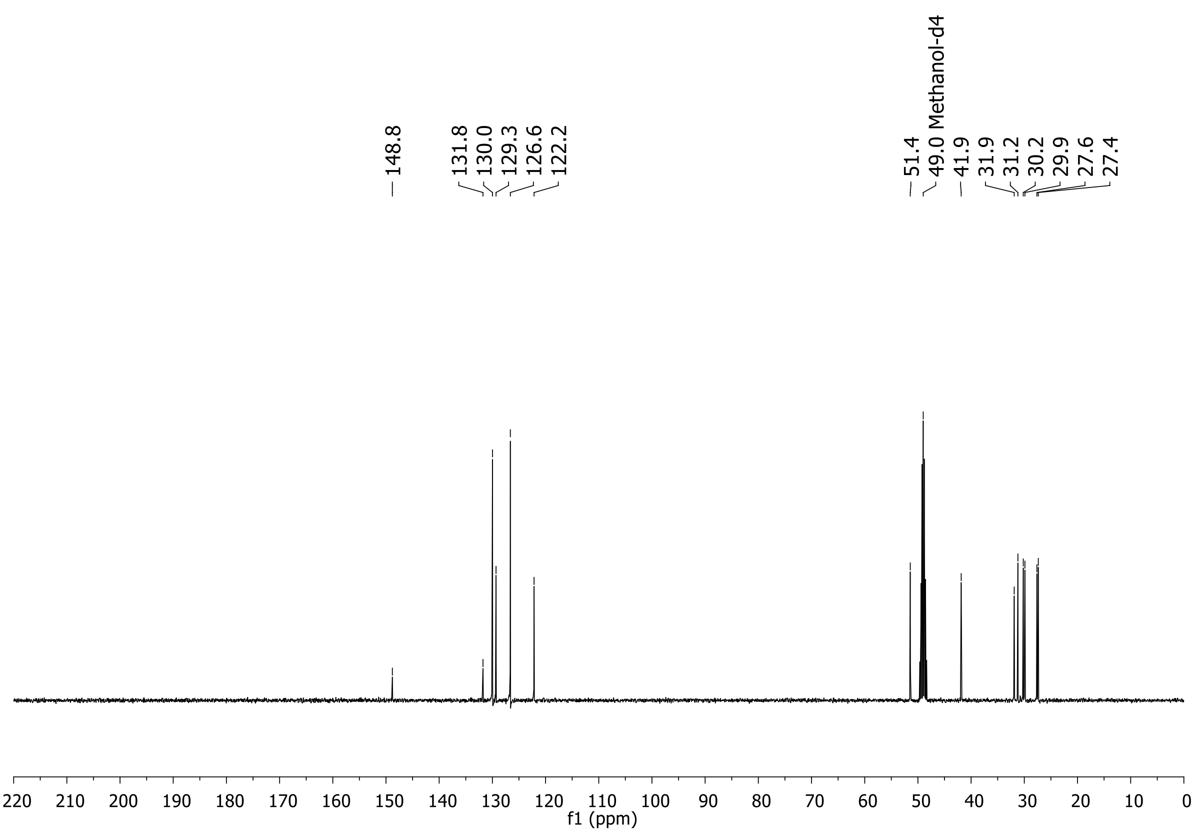

^{13}C-NMR spectrum of compound **42** (50 MHz, CD_3OD).

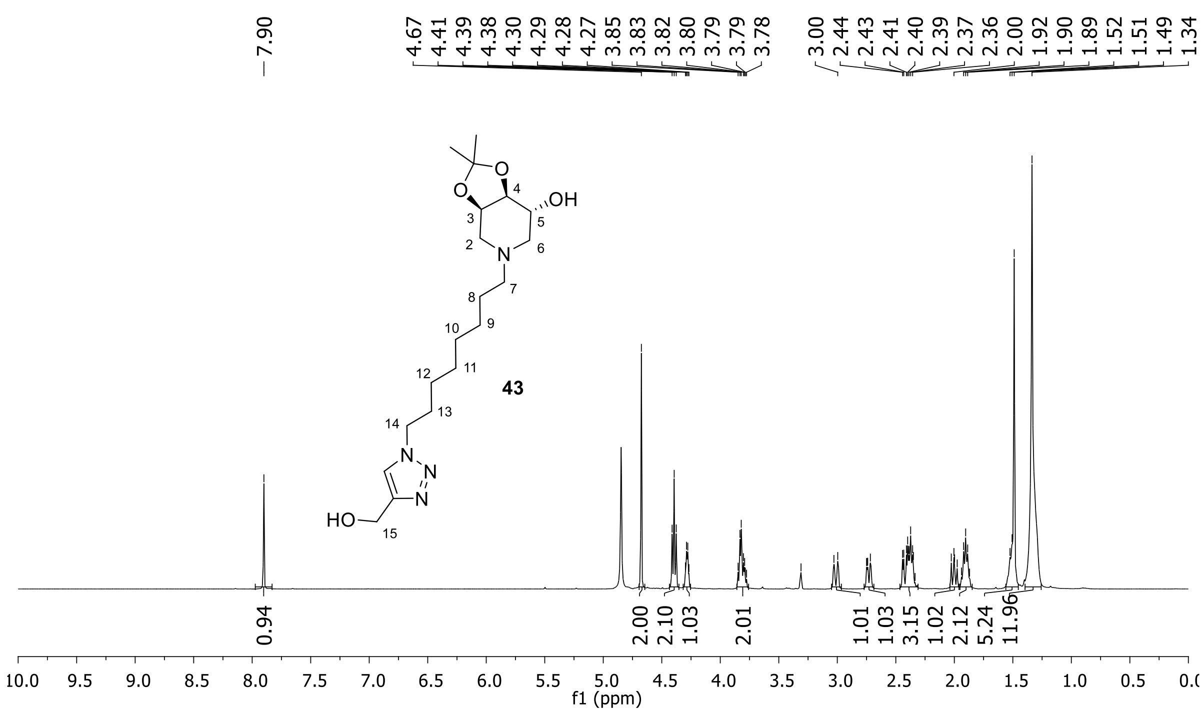

^{1}H-NMR spectrum of compound **43** (400 MHz, CD_3OD).

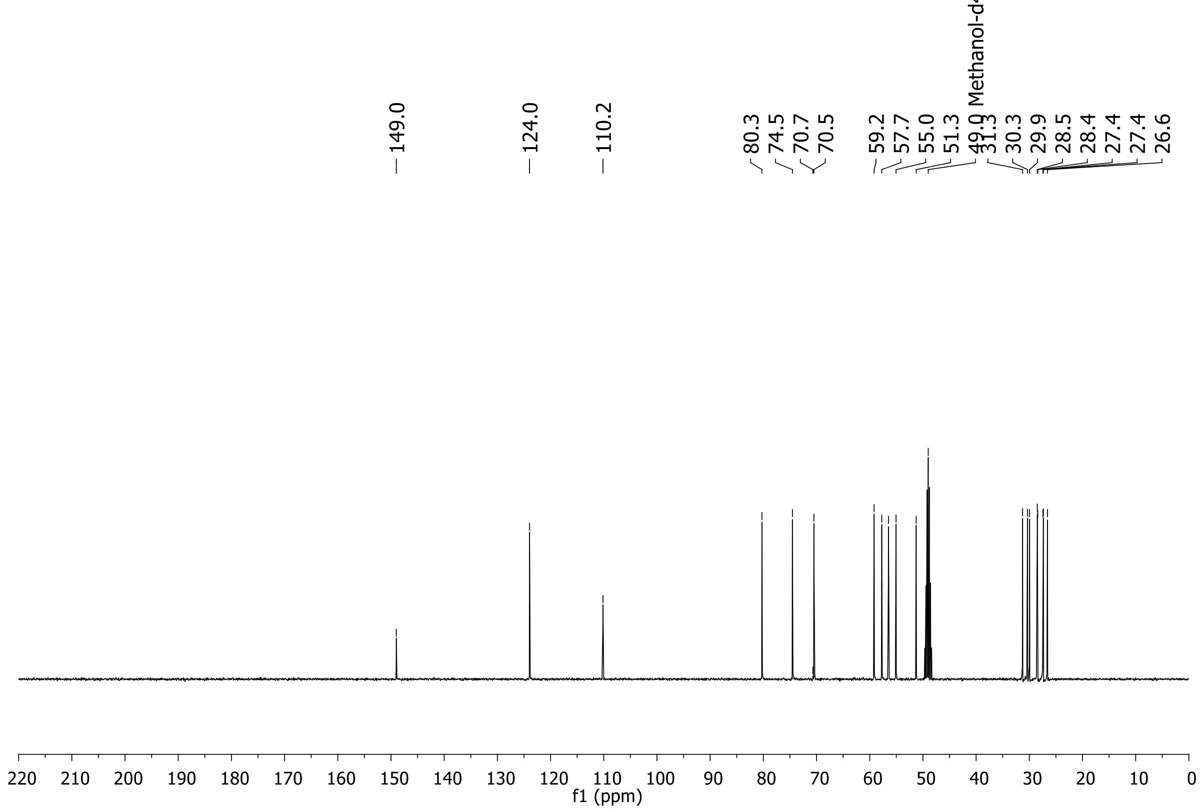

^{13}C-NMR spectrum of compound **43** (100 MHz, CD_3OD).

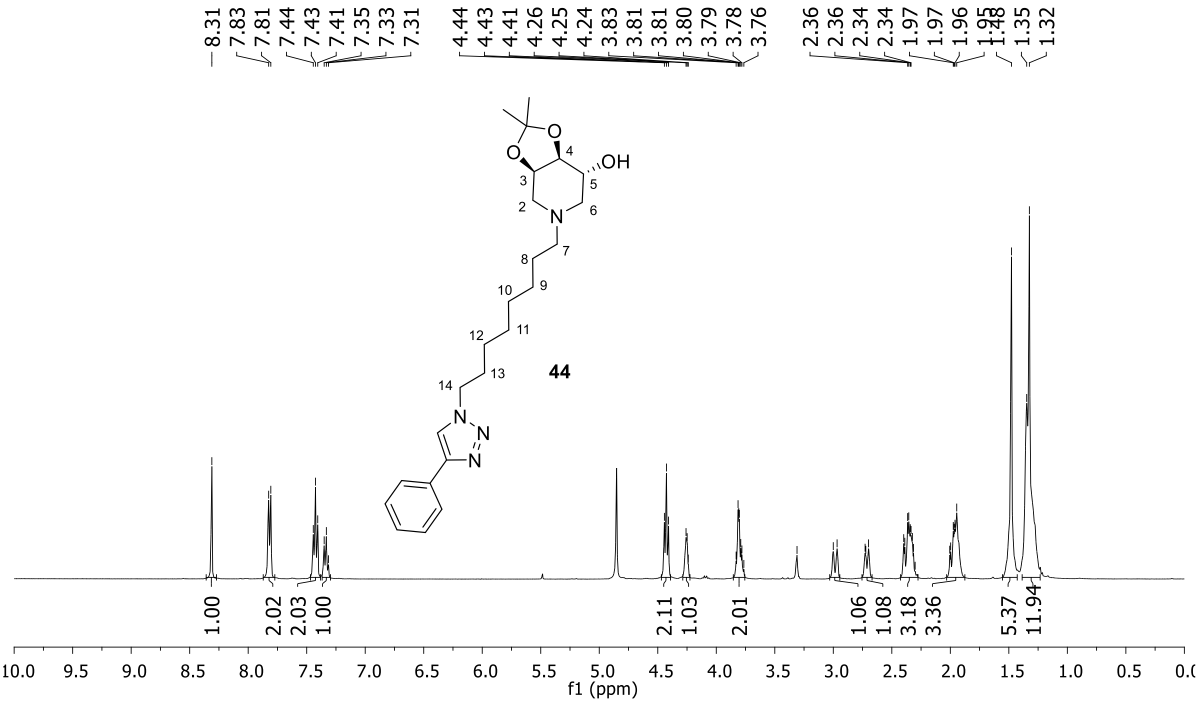

^{1}H-NMR spectrum of compound **44** (400 MHz, CD_3OD).

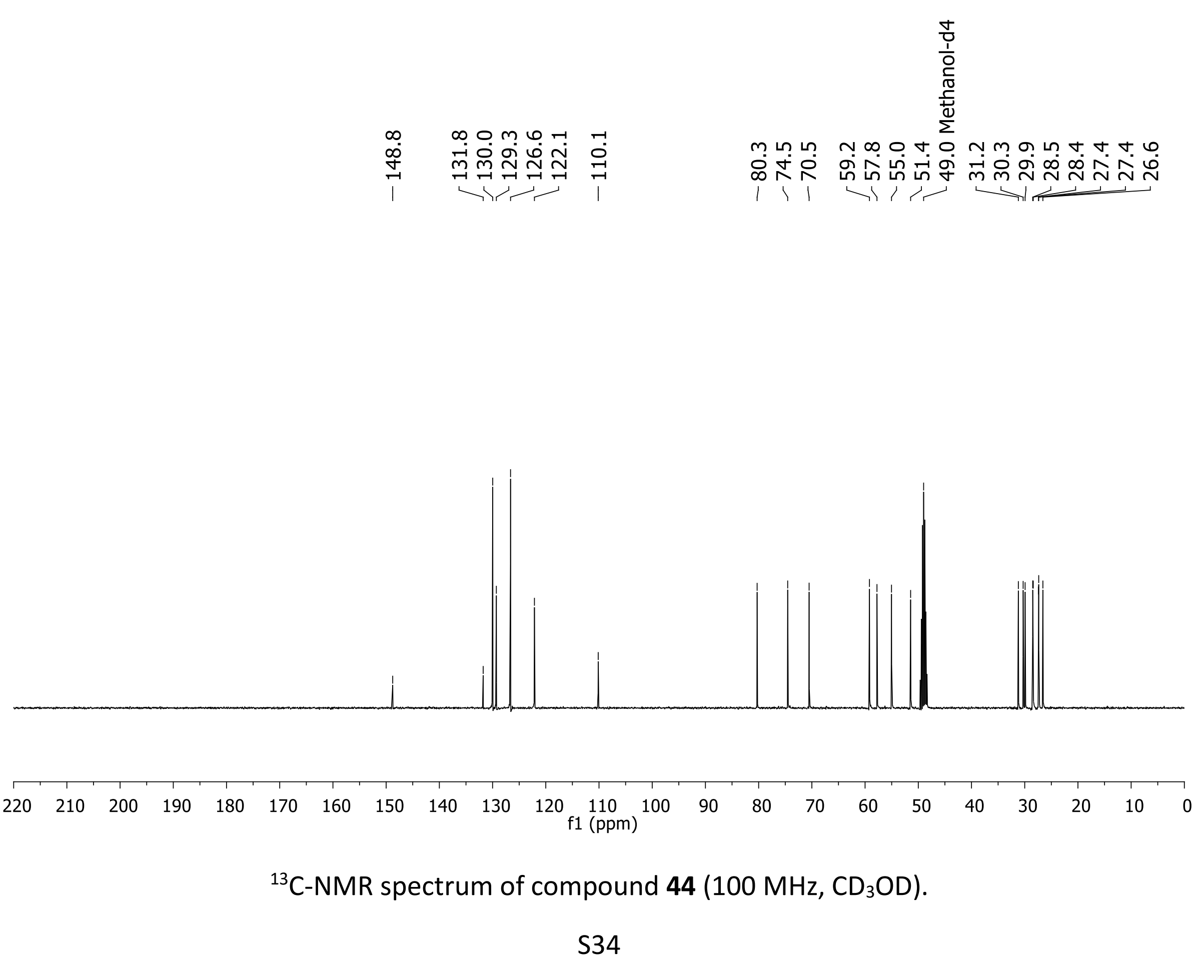

^{13}C-NMR spectrum of compound **44** (100 MHz, CD_3OD).

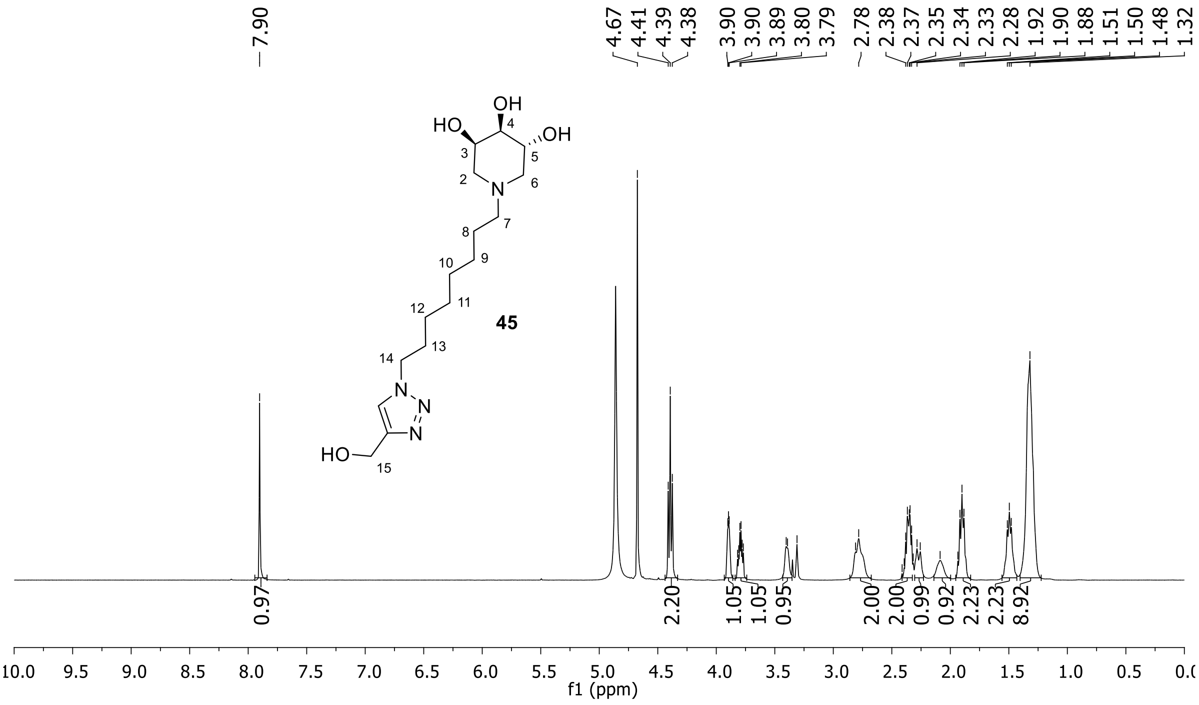

^{1}H-NMR spectrum of compound **45** (400 MHz, CD_3OD).

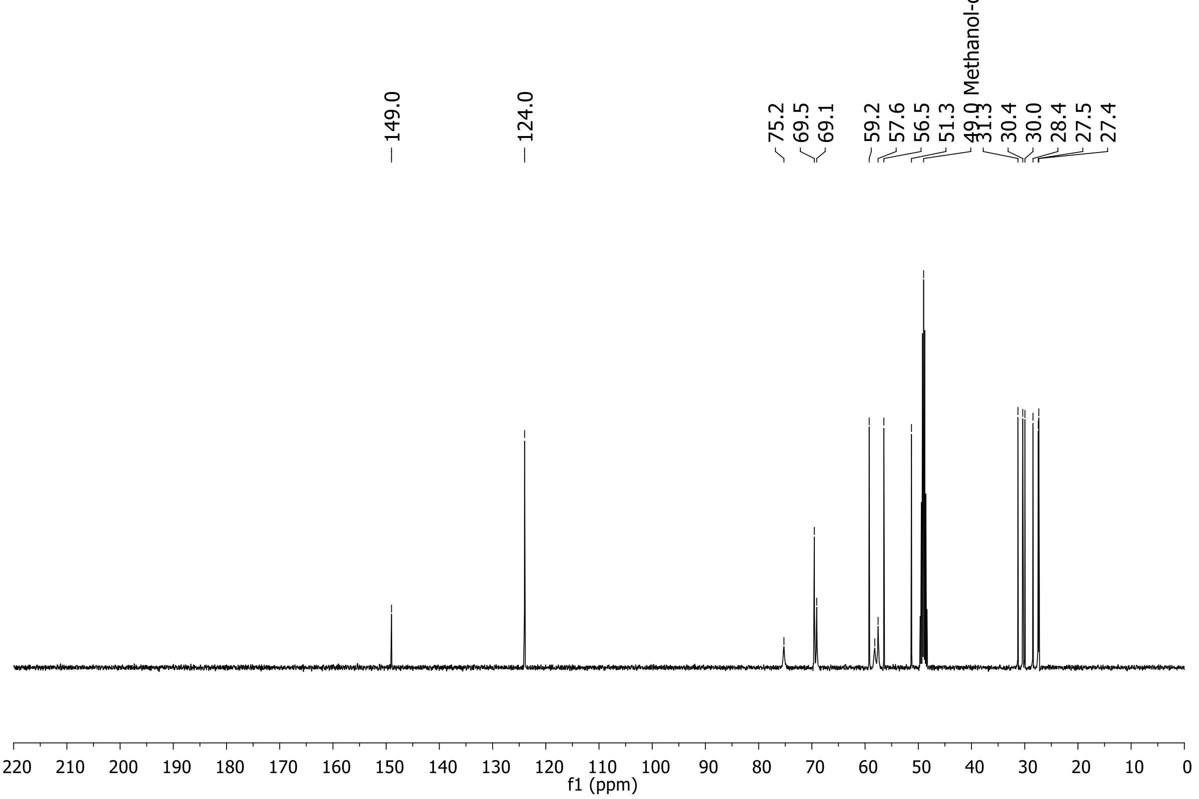

^{13}C-NMR spectrum of compound **45** (100 MHz, CD_3OD).

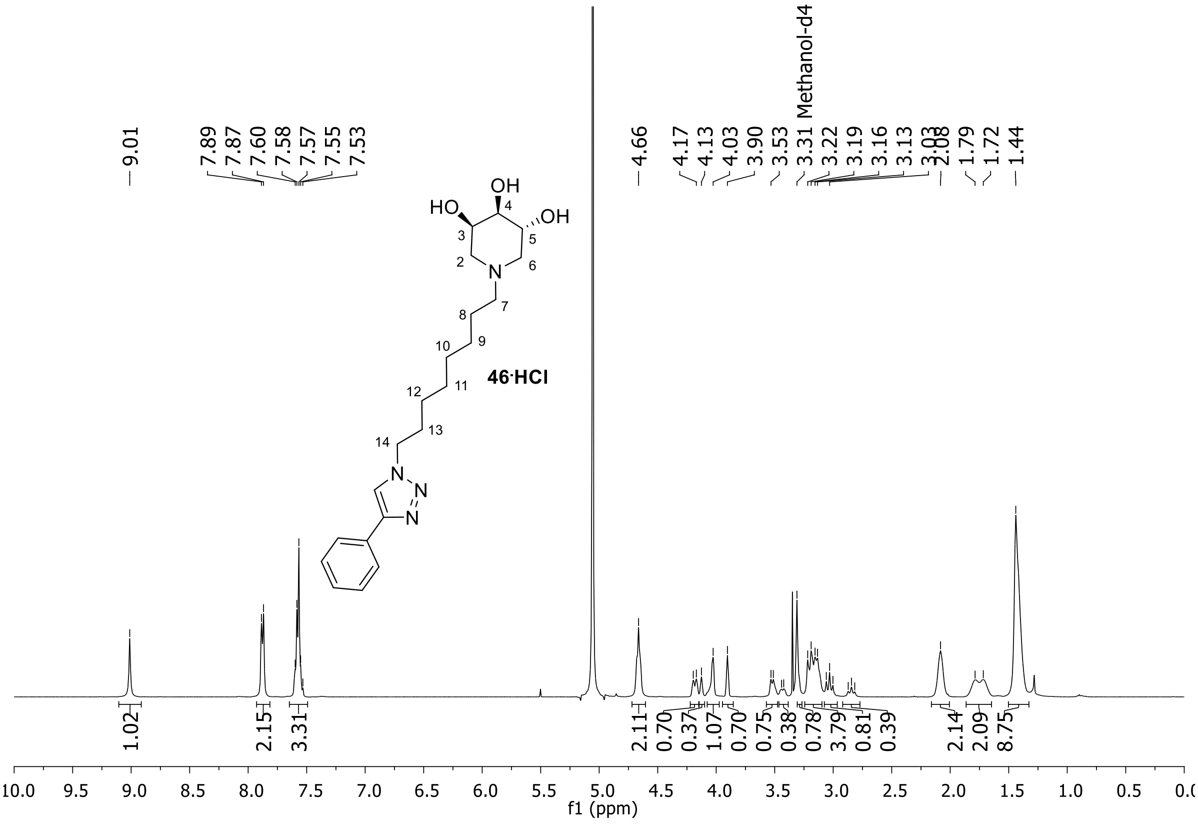

^{1}H-NMR spectrum of compound **46·HCl** (400 MHz, CD_3OD).

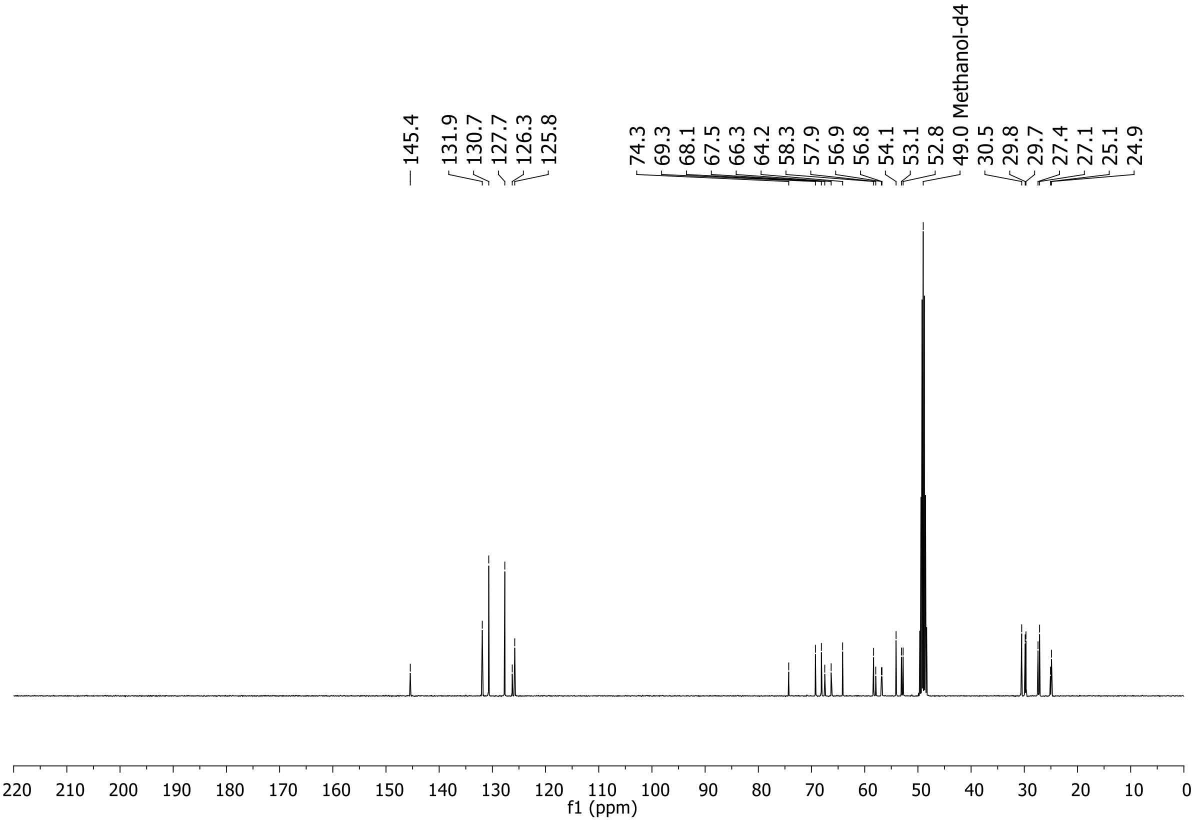

^{13}C-NMR spectrum of compound **46·HCl** (100 MHz, CD_3OD).

Inhibitory activity towards human GCase from leukocyte homogenates.

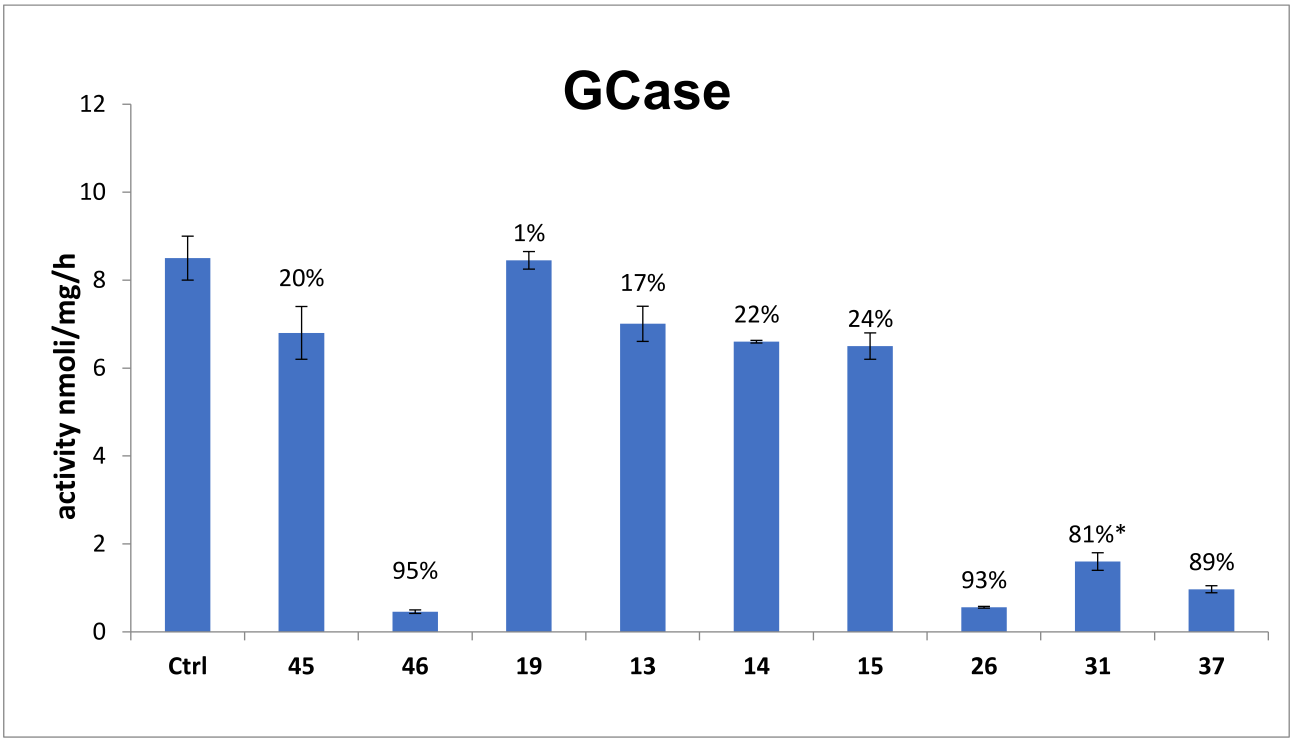

Figure S2: Activity of GCase in the presence of compounds **45**, **46**, **19**, **13-15**, **26** and **37** at 1 Mm and compound **31** at 0.1 mM. The corresponding calculated percentage of inhibition is indicated above each bar.

IC_{50} determination and curves

The IC_{50} values of **26**, **31**, **37**, **45** and **46** against GCase were determined by measuring the initial hydrolysis rate of 4-methylumbelliferyl-β-D-glucoside substrate (3.33 mM) in the presence of increasing inhibitor concentrations. All tests were carried out in triplicate. Data show in the figure represent the mean value ± SD. Data were fitted to the following equation using the Origin Microcal program.

$$\frac{Vi}{Vo} = \frac{Max - Min}{1 + \left(\frac{x}{IC_{50}}\right)^{slope}} + Min$$

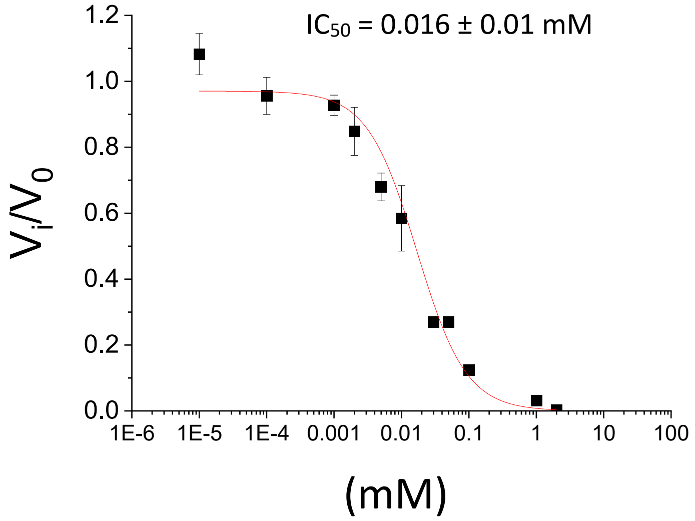

Figure S3: IC_{50} graph of compound **26**.

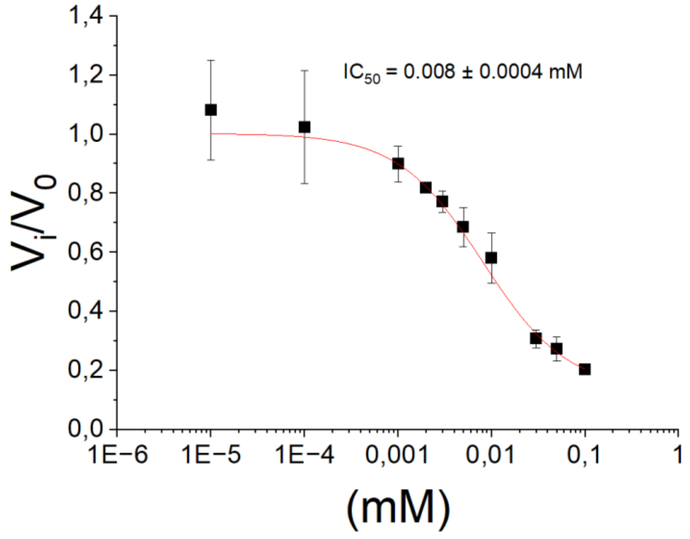

Figure S4: IC_{50} graph of compound **31**.

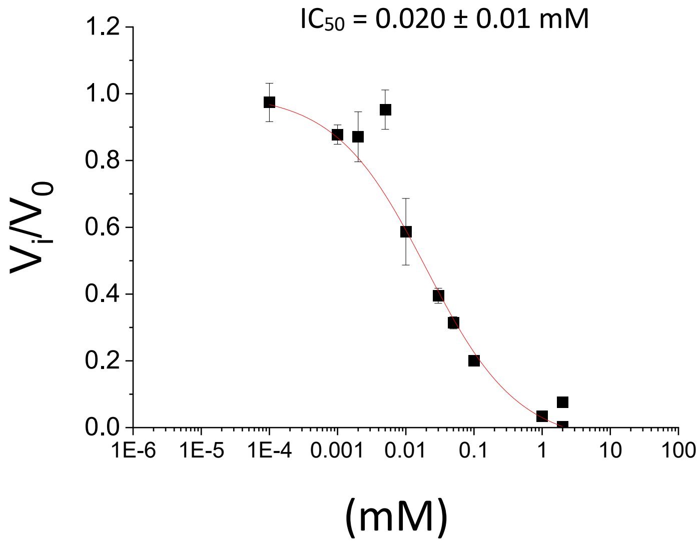

Figure S5: IC_{50} graph of compound **37**.

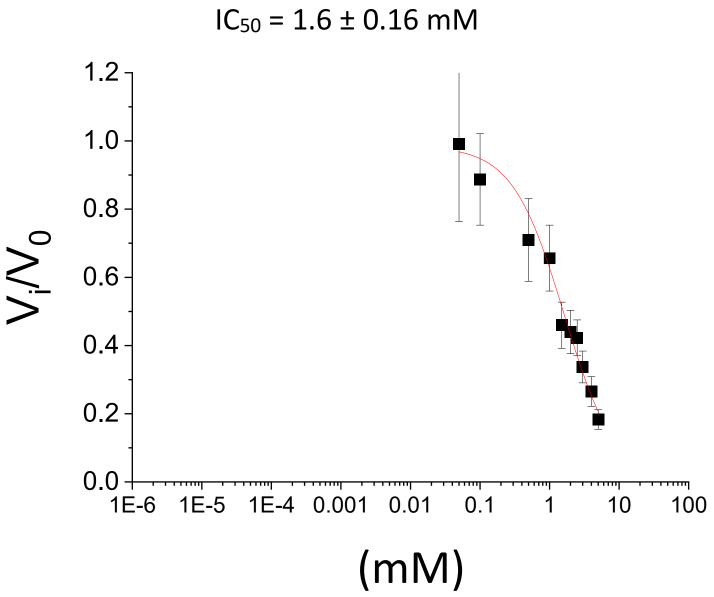

Figure S6: IC_{50} graph of compound **45**.

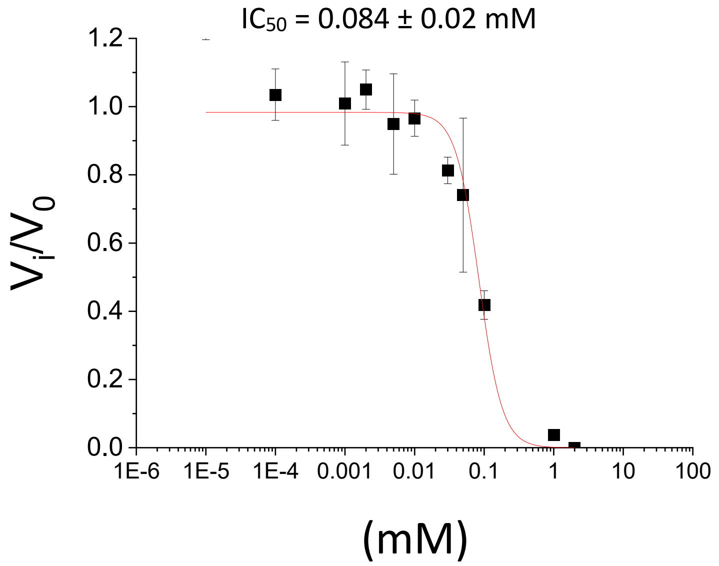

Figure S7: IC_{50} graph of compound **46**.

Kinetic analysis for compounds 26, 31, 37 and 46

Regarding compound **26**, we tested three different concentrations, namely 6, 12 and 18 µM. Results obtained are shown in Figure S8.

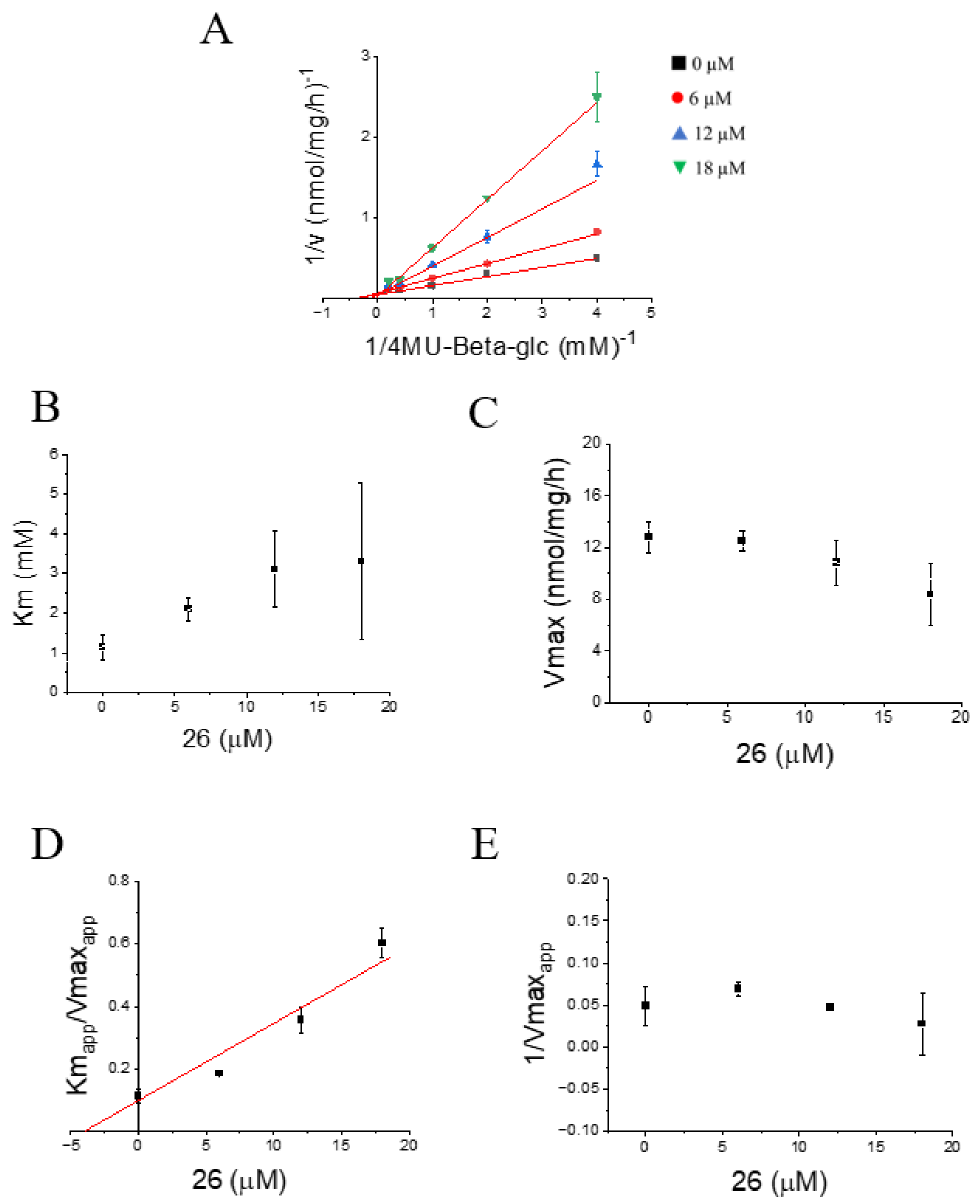

Figure S8: **Kinetic analysis of compound 26.** (A) Double reciprocal plots. 4-Methylumbelliferyl-β-D-glucoside is employed as a substrate. The concentrations of compound **26** are: ■, 0 µM; ●, 6 µM; ▲, 12 µM; ▼, 18 µM. Data reported in the figures represent the mean values ± S.E.M. (n = 3). (B, C) Behaviour of K_m and V_{max} at different concentrations of compound **26**. (D, E) To determine the K*i* value, we plotted the slope ($Km_{app}/Vmax_{app}$) as a function of inhibitor concentration.

The results of the kinetic analyses revealed that compound **26** causes a dose-dependent increase in Km and a slight reduction in Vmax. However, analysis of the secondary plots (Fig. S8 D and E) shows that the $Km_{app}/Vmax_{app}$ ratio increases steadily with increasing concentrations of compound **26**, while the $1/Vmax_{app}$ value remains constant. Together, these findings indicate that compound **26** acts as a pure competitive inhibitor. Therefore, by plotting the slopes of the straight lines against the inhibitor concentration, the K*i* value can be determined, which was found to be 3.30 ± 0.24 µM.

Regarding compound **31**, we tested three different concentrations, namely 3, 6 and 9 µM. Results obtained are shown in Figure S9.

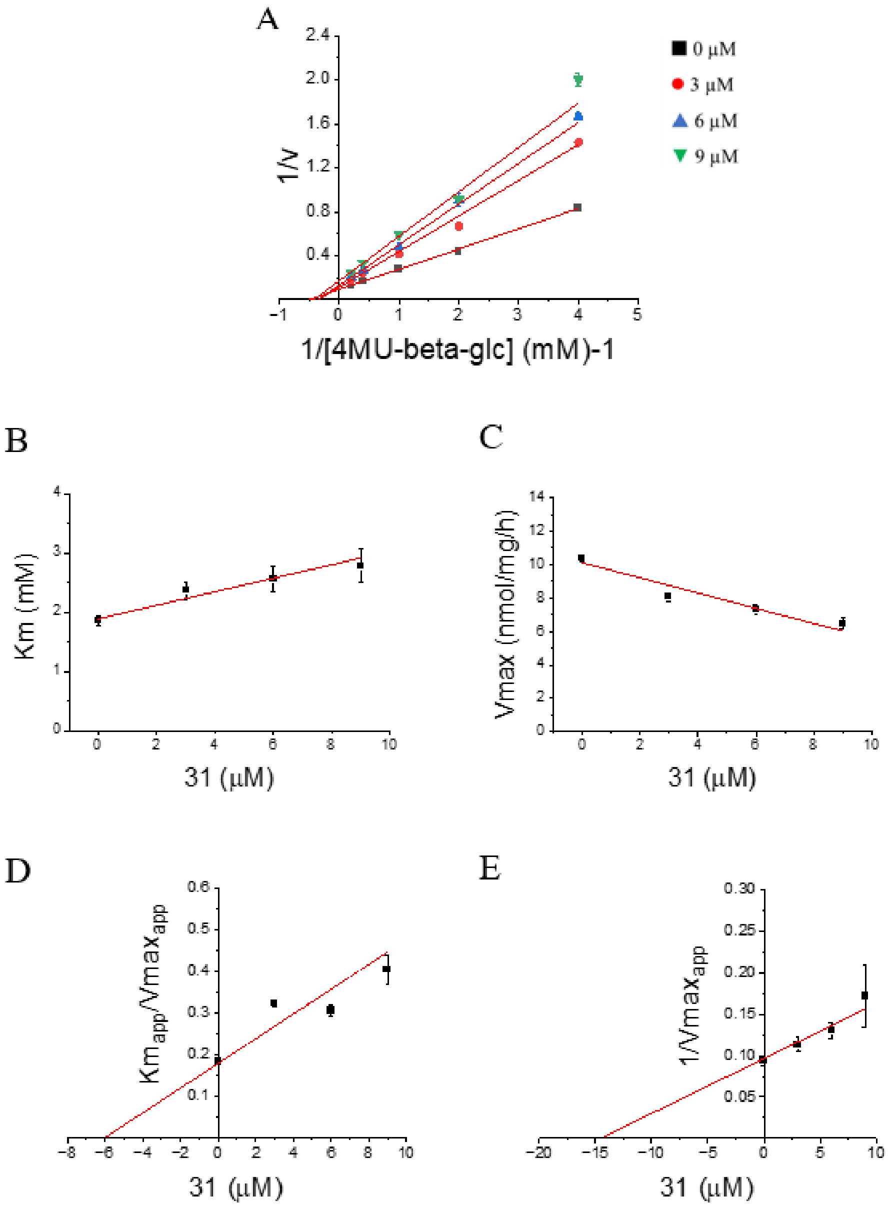

Figure S9: **Kinetic analysis of compound 31.** (A) Double reciprocal plots. 4-Methylumbelliferyl-β-D-glucoside is employed as a substrate. The concentrations of compound **31** used were: ■, 0 µM; ●, 3 µM; ▲, 6 µM; ▼, 9 µM. Data reported in the figures represent the mean values ± S.E.M. (n = 3). (B, C) Behaviour of K_m and V_{max} at different concentrations of compound **31**. (D, E) To determine both the K*i* and K*i'* values, we plotted the slope ($Km_{app}/Vmax_{app}$) and the intercept values ($1/Vmax_{app}$) as a function of the inhibitor concentration.

As compound **31** concerns, the results of the kinetic analysis revealed that it causes a dose-dependent increase in Km (S9B) and a decrease in Vmax (S9C). However, the secondary plots show that not only $Km_{app}/Vmax_{app}$ versus compound **31** concentration increases (S9D), but also $1/Vmax_{app}$ (S9E) increases, demonstrating a real decrease of the Vmax. This finding suggests that compound **31** behaves as a mixed-type inhibitor**.** Therefore, by plotting the slopes ($Km_{app}/Vmax_{app}$) and the intercept ($1/Vmax_{app}$) values of the straight lines against the inhibitor concentration, we calculated both the K*i* (6.0 ± 0.1 µM) and K*i'* (14.5 ± 0.3 µM) values.

Regarding compound **37**, we tested three different concentrations, namely 5, 10 and 20 μM. Results obtained are shown in Figure S10.

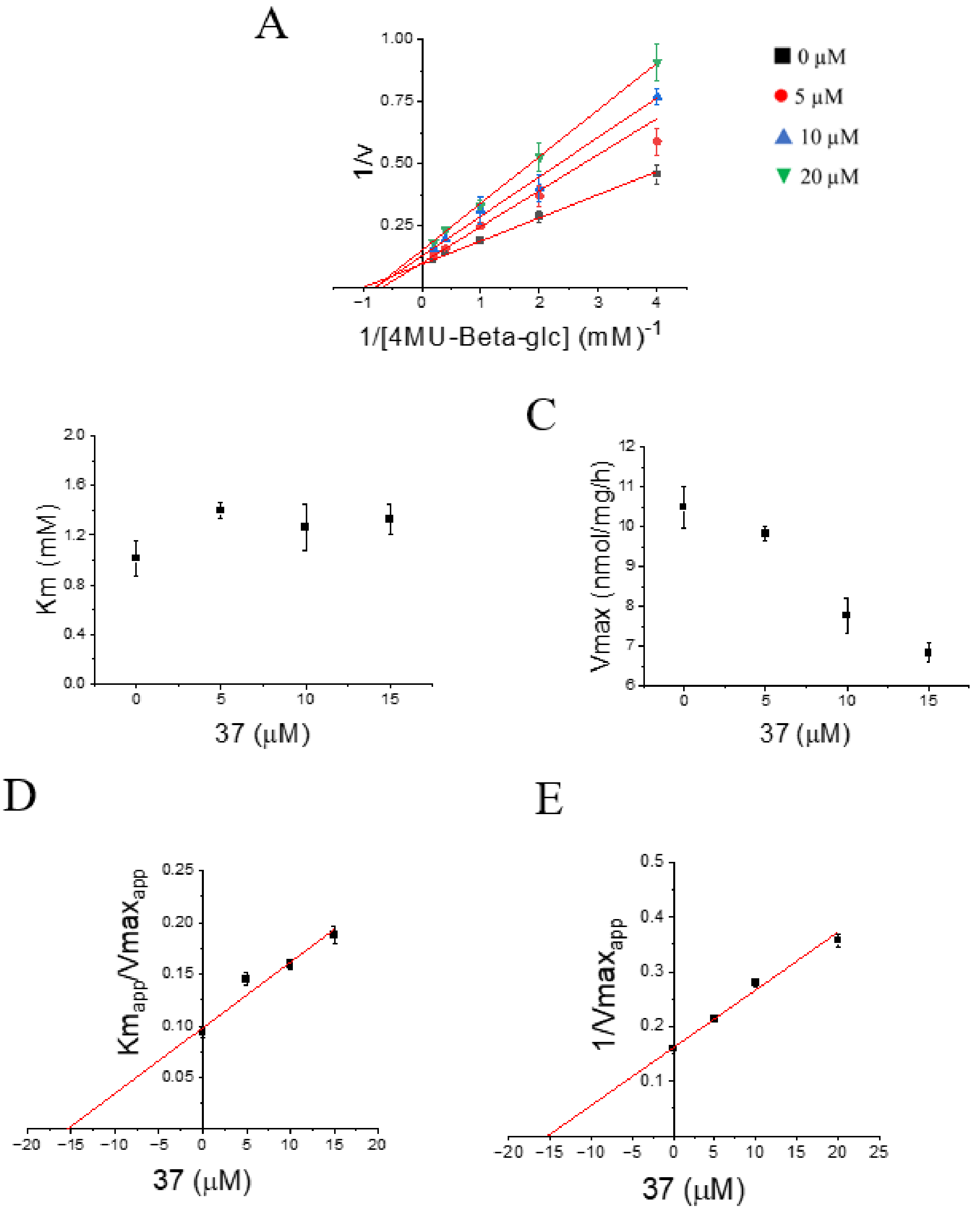

Figure S10: **Kinetic analysis of compound 37.** (A) Double reciprocal plots. 4-Methylumbelliferyl-β-D-glucoside is employed as a substrate. The concentrations of compound **37** used were: ■, 0 μM; ●, 5 μM; ▲, 10 μM; ▼, 15 μM. Data reported in the figures represent the mean values ± S.E.M. (n = 3). (B, C) Behaviour of K_m and V_{max} at different concentrations of compound **37**. (D) To determine the K*i* value, we plotted the slope ($Km_{app}/Vmax_{app}$) as a function of inhibitor concentration.

Data obtained from kinetic analyses carried out with compound **37** showed that it determines an increase of Km value (10B) and a decrease of Vmax (S10C) suggesting that it behaves as a mixed type inhibitor.
In agreement with these results, double reciprocal plot shows that experimental points describe straight lines intersecting one each other in the left quadrant (S10A), confirming the above hypothesis. Therefore, by plotting the slopes ($Km_{app}/Vmax_{app}$, S10D) and the intercept ($1/Vmax_{app}$, S10E) values of the straight lines against the inhibitor concentration, we calculated both the K*i* (9.7 ± 0.5 μM) and K*i'* (15.2 ± 0.4 μM) values.

Regarding compound **46**, we tested three different concentrations, namely 40, 80 and 120 µM. Results obtained were shown in Figure S11.

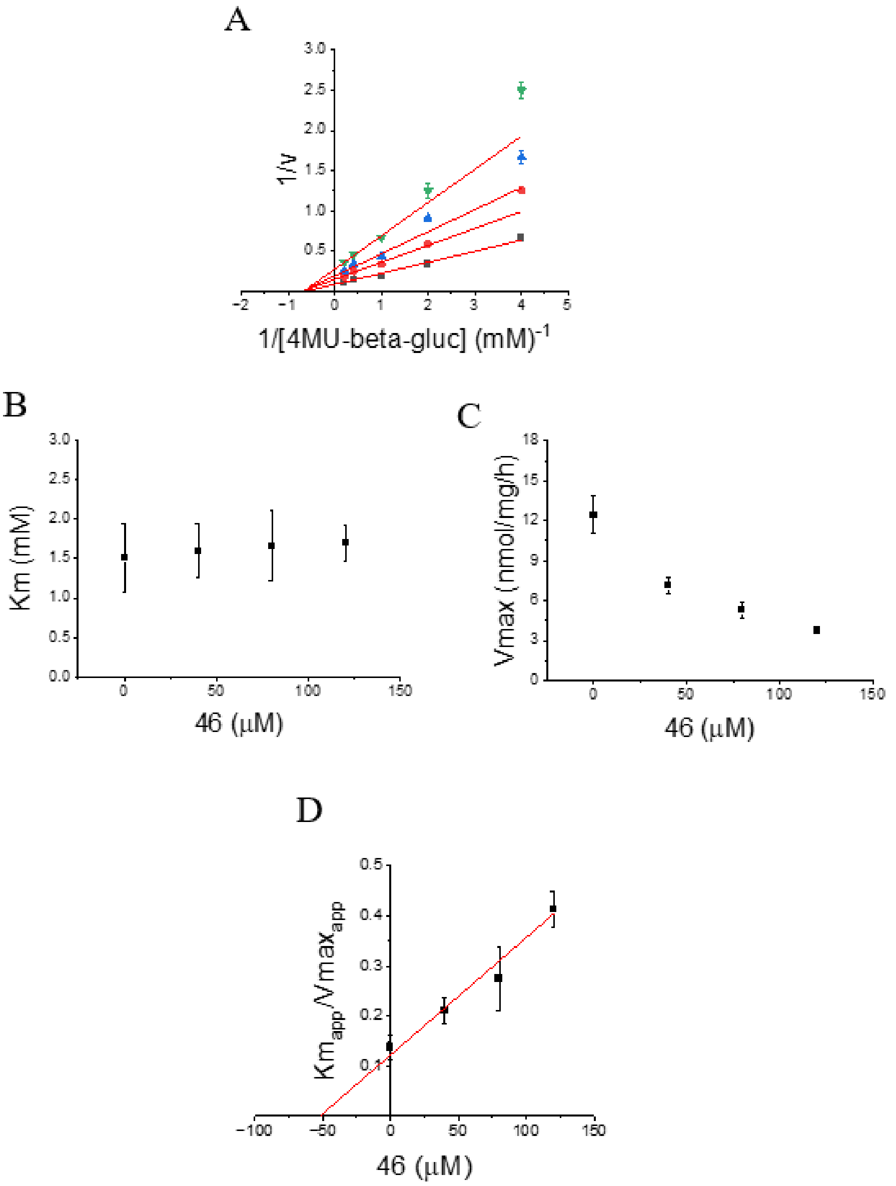

Figure S11: **Kinetic analysis of compound 46.** (A) Double reciprocal plots. 4-Methylumbelliferyl-β-D-glucoside is employed as a substrate. The concentrations of compound 46 used were: ■, 0 µM; ●, 40 µM; ▲, 80 µM; ▼, 120 µM. Data reported in the figures represent the mean values ± S.E.M. (n = 3). (B, C) Behaviour of K_m and V_{max} at different concentrations of compound **46**. (D) To determine the K*i* value, we plotted the slope ($Km_{app}/Vmax_{app}$) as a function of inhibitor concentration.

The Lineweaver-Burk plot shows that the experimental points, obtained by assaying the enzyme activity in the presence of increasing concentrations of compound **46**, form straight lines with different slopes that intersect in the left quadrant. Moreover, we observed that Vmax decreases with increasing concentrations of compound **46**, while the Km value remains unaffected. Together, these findings suggest that compound **46** acts as a **non-competitive inhibitor**. Therefore, using the appropriate equation, the K*i* value was calculated to be **51.0 ± 2.9 µM**.

Computational Studies

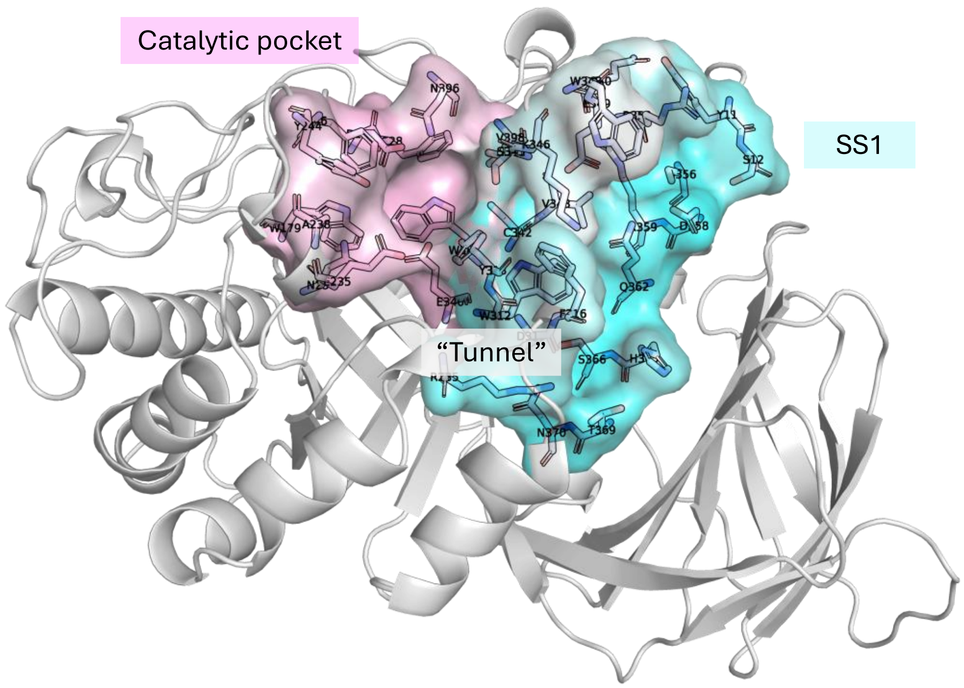

Figure S12: Surface representation of GCase highlighting the two principal binding regions identified through cavity mapping with Cb-Dock2. The catalytic pocket (pink), which accommodates the substrate and competitive inhibitors such as isofagomine, serves as the primary recognition site. A secondary internal cavity, SS1 (cyan), is accessible via a narrow tunnel (grey) formed by loops L314–L317 and K346–E349.
